# Computational and experimental investigation of the effect of cation structure on the solubility of anionic flow battery active-materials†

**DOI:** 10.1039/d1sc04990a

**Published:** 2021-11-26

**Authors:** Benjoe Rey B. Visayas, Shyam K. Pahari, Tugba Ceren Gokoglan, James A. Golen, Ertan Agar, Patrick J. Cappillino, Maricris L. Mayes

**Affiliations:** Department of Chemistry and Biochemistry, University of Massachusetts Dartmouth MA 02747-2300 USA maricris.mayes@umassd.edu pcappillino@umassd.edu; Department of Mechanical Engineering, Energy Engineering Graduate Program, University of Massachusetts Lowell Lowell MA 01854 USA

## Abstract

Recent advances in clean, sustainable energy sources such as wind and solar have enabled significant cost improvements, yet their inherent intermittency remains a considerable challenge for year-round reliability demanding the need for grid-scale energy storage. Nonaqueous redox flow batteries (NRFBs) have the potential to address this need, with attractive attributes such as flexibility to accommodate long- and short-duration storage, separately scalable energy and power ratings, and improved safety profile over integrated systems such as lithium-ion batteries. Currently, the low-solubility of NRFB electrolytes fundamentally limits their energy density. However, synthetically exploring the large chemical and parameter space of NRFB active materials is not only costly but also intractable. Here, we report a computational framework, coupled with experimental validation, designed to predict the solubility trends of electrolytes, incorporating both the lattice and solvation free energies. We reveal that lattice free energy, which has previously been neglected, has a significant role in tuning electrolyte solubility, and that solvation free energies alone is insufficient. The desymmetrization of the alkylammonium cation leading to short-chain, asymmetric cations demonstrated a modest increase in solubility, which can be further explored for NRFB electrolyte development and optimization. The resulting synergistic computational–experimental approach provides a cost-effective strategy in the development of high-solubility active materials for high energy density NRFB systems.

## Abstract 2

Active-material solubility is critical in determining NRFB energy density, yet a predictive model accounting for solid-state cohesion energy has remained elusive. Herein we present such, based on an empirically calibrated computational framework.

## Graphical abstract

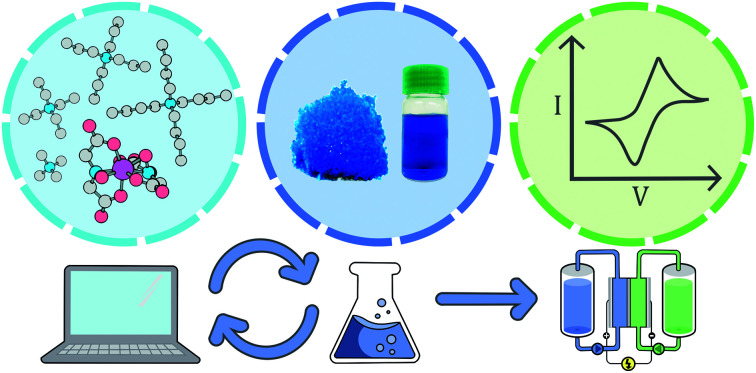

## 1. Introduction

Current technological improvements, growing global concern for CO_2_ emissions, and the rapid increase of energy demand have contributed to a significant lowering of the cost for renewable energy generation, with wind and solar sources being the main contributors.^1–3^ Despite these improvements, the inherent intermittency of these sources still poses a considerable challenge towards the year-round reliability of these renewable energy sources, necessitating grid-scale energy storage.^2,4–7^ Redox flow batteries (RFBs) are considered one of the most promising electrochemical technologies for the large-scale storage of renewable electrical energy.^8,9^ A key advantage of RFB systems is the highly scalable, uncoupled power rating (area of the electrode) and energy capacity (amount of electrolyte), providing excellent flexibility for a range of stationary storage applications. Of particular interest in the field is the development of nonaqueous redox flow battery (NRFB) systems. Polar organic solvents have a wide electrochemical stability window, allowing higher cell voltages and energy densities than water.^8,10–12^

Despite having great potential for grid-scale application, NRFB technology is still in its infancy with technical hurdles that must be overcome, including low solubility of active-materials, which limits energy density, and poor stability, leading to low cyclability.^13–16^ To date, most research has focused on improving the performance of individual components such as high-energy-capacity electrolytes.^17^ Moreover, electrolyte viscosity, which is correlated with conductivity and diffusivity, is another important issue in the development of NRFB systems and is gaining attention in the community.^18–20^ Electrolyte systems with low viscosity, high diffusivity, and high ionic conductivity are ideal.^12,21^ The demand for higher energy density systems, pushing towards higher active material concentration, has resulted in higher viscosity in NRFB electrolytes which hinders practical applications due to pumping losses and decreased conductivity.^19^

**Fig. 1:**
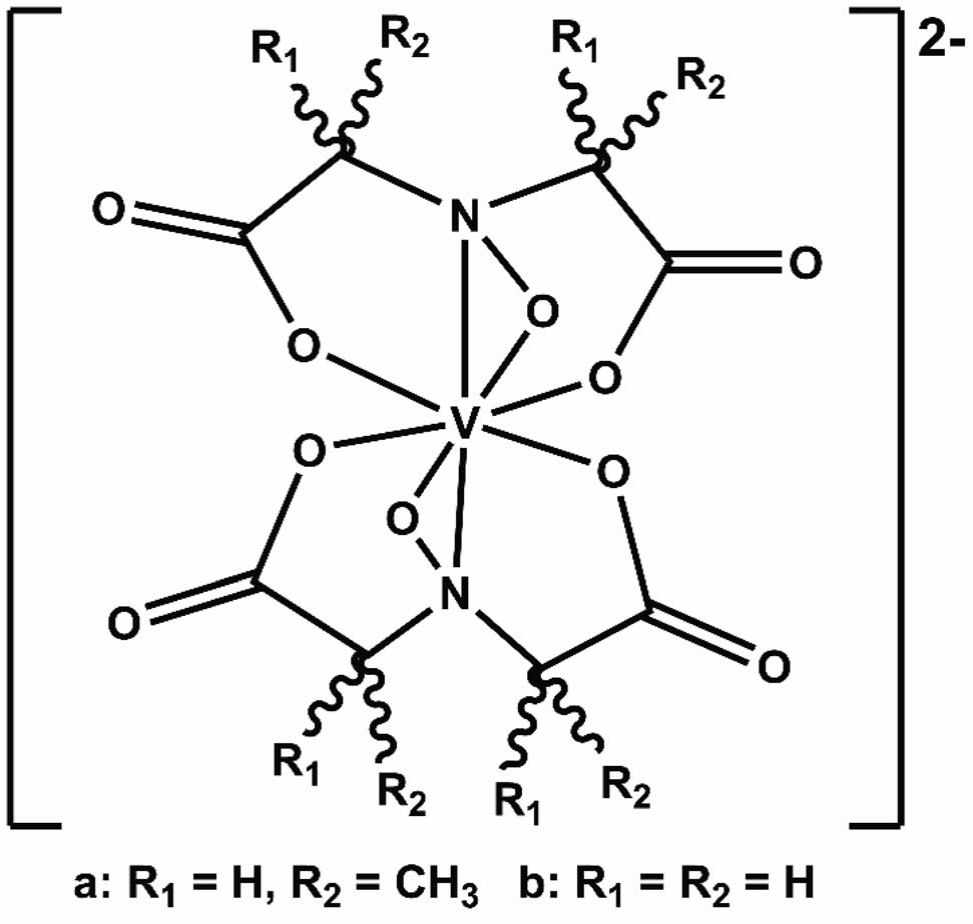
Amavadin (a), a naturally occurring vanadium chelate, isolated from mushrooms in the *Amanita* genus and (b) the related, proteo-analog, [VBH]^2−^.

We recently elaborated on an NRFB active-material, leveraging a bio-inspired redox molecule^5,22,23^ known as Amavadin (Fig. 1a), biosynthesis of which has evolved naturally in mushrooms of the *Amanita* genus under selection pressure for strong and specific vanadium binding. As a result, this compound and its analogs exhibit the highest stability constants ever measured for a V^4+^ ion.^24^ Using inexpensive reagents, vanadium (4+) bis-hydroxyiminodiacetic acid ([VBH]^2−^) (Fig. 1b) has been synthesized at a large scale which exhibits high chemical stability, even under cycling at high current and to deep states-of-charge.^23^ More recently, we demonstrated a synthetic strategy to increase the solubility of VBH-based NRFB materials while still maintaining stability during deep cycling for extended time periods.^5^ This highlights the potential of VBH-based NRFB materials as a molecular scaffold for the development of next-generation flow battery electrolytes.

**Fig. 2:**
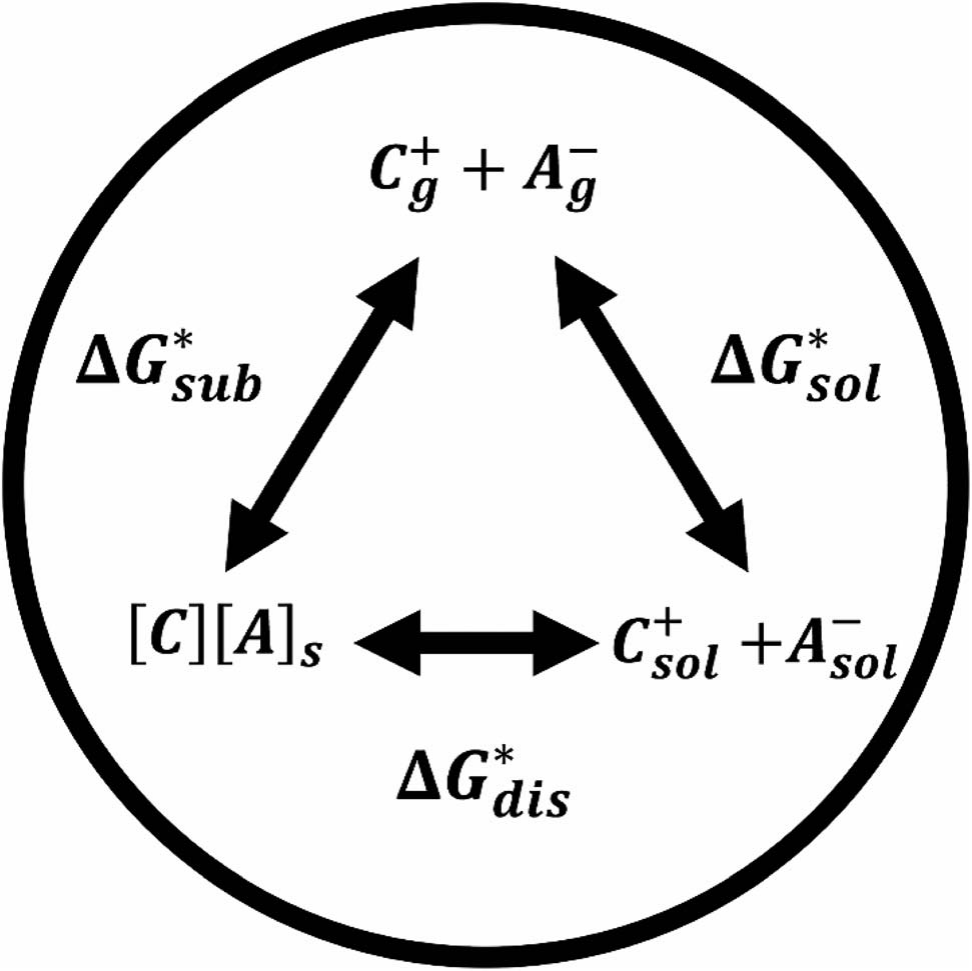
Schematic representation of the thermodynamic cycle for the dissolution of [cation][VBH] complexes. The free-energy of dissolution 
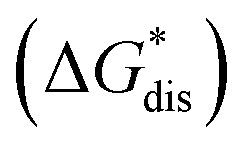
 determines the solubility of the complex and results from the interplay between the free energy of sublimation 
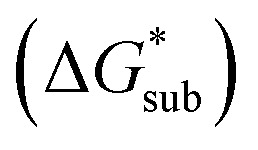
 and that of the stabilizing solute–solvent interactions in solution 
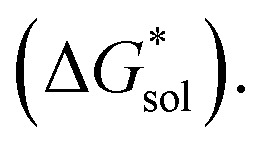

The solubility of active-materials is a critical factor in determining the energy density of NRFBs.^2,25^ However, synthetically exploring a large parameter space to identify soluble species empirically is an inefficient approach. Solubility can be calculated by comparing the stabilization of the gas-phase ions upon solvation 
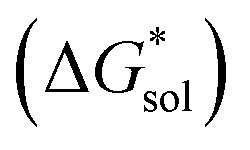
 with that of the organization of the ions into a crystal lattice 
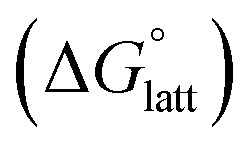
 (see Fig. 2). In the well-known thermochemical Born–Fajans–Haber cycle (Fig. 2),^26^ the latter factor can be expressed in terms of free energy of sublimation 
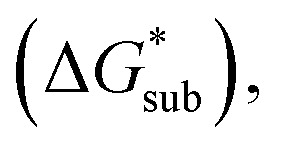
 which describes the energetics of a material's phase change from solid to gas, and is approximately the negative of 
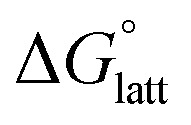
 (see ESI,† eqn (2)). This thermochemical cycle has been utilized in previous solubility studies in pharmaceutics,^27–29^ druglike molecules,^30,31^ and systems like LiO_2_ and Li_2_O_2_.^32^ These works involved neutral molecules and disregarded some thermochemical corrections. With full thermochemical corrections, a similar approach was used by Chen and Bryantsev to predict the melting points of ionic liquids.^33^

Despite the straightforward description of solubility, accurate calculations of 
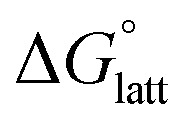
 have proven challenging and computationally expensive.^33^ As such, recent computational approaches to determine RFB active-material solubility have focused only on 
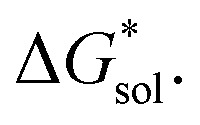
^5,25^ In our previous work,^5^ we demonstrated that solvation energies are insufficient to describe the experimental solubility trend for VBH with long-chain alkylammonium counter cations. This implies a critical role for 
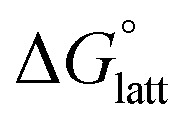
 in determining the solubility of active materials in NRFB electrolytes and highlights the importance of its accurate calculation in any quantitively useful prediction of solubility.

**Fig. 3:**
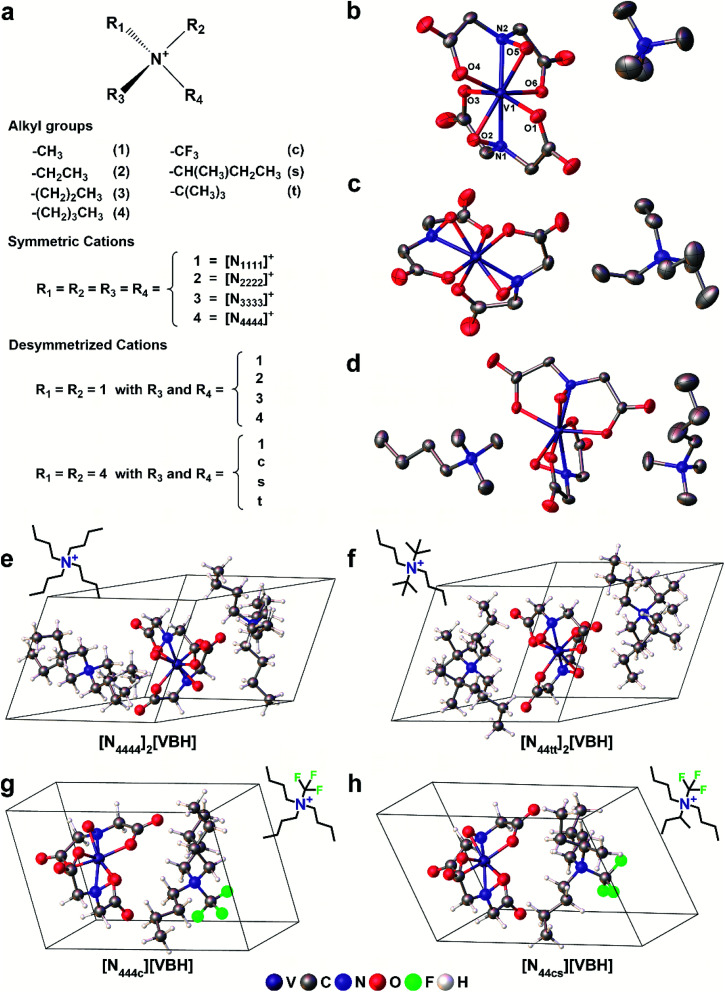
Scheme representing the different (a) alkyl ammonium cations used. Each alkyl group is represented by a single character and is used in the alkyl ammonium notation (*e.g.* [N_1111_]^+^). X-Ray crystallographic thermal ellipsoid plots at 30% probability of (b) [N_1111_][VBH], (c) [N_2222_][VBH], and (d) [N_1114_]_2_[VBH]. Representative structures of the DFT optimized (e) symmetric, (f) symmetric di-substituted, (g) monosubstituted, and (h) asymmetric di-substituted simplified crystals with the corresponding structural formulae of the cations as insets.

In this paper, we report a computational framework designed to predict the solubility trends of electrolytes, tracking both the thermodynamic contributions due to free energy of active-material/solvent interactions and free-energy of the active-material crystal lattice. These calculations make use of extensive information on the solid-state geometries of VBH with various cations, provided by eight crystal structures, for input geometries. These structures, correspond to the reduced and oxidized forms of [VBH] with symmetrical alkylammonium cations, having one to four carbon atoms in each chain. Two of these are newly reported, herein (see Fig. 3b, c and Table S6†), and five of these have been reported previously.^5,22,23^ One of the eight structures, corresponding to [N_3333_]_2_[VBH], was of high enough quality to obtain structural information (unit cell, Cartesian coordinates, and bonding parameters for starting geometries) but not of publication quality. The solubility predictions were validated by preparing and characterizing several [VBH] compounds with symmetric and asymmetric counter-cations in +4 (*e.g.*, Fig. 3d) and +5 (*e.g.*, Fig. 3b) redox states and measuring their solubility in various solvents. This work provides valuable insights into tuning electrolyte solubility. We present a synergistic computational–experimental design approach focusing on short-chain, asymmetric alkylammonium cations that would potentially yield highly soluble VBH active material. Lastly, we briefly describe the viscosities of the electrolyte solutions as well as the role of solvent.

## 2. Results and discussion

### 2.1. Experimental solubilities of symmetric alkylammonium [VBH] compounds

To investigate the solubilities of VBH-based active materials, we synthesized the alkylammonium [VBH] compounds in their reduced (+4) and oxidized (+5) states ([N_*xxxx*_]_2_[V(+4)BH] and [N_*xxxx*_][V(+5)BH], where *x* = 1 (methyl), 2 (ethyl), 3 (*n*-propyl), 4 (*n*-butyl)) and measured their solubilities in tetrahydrofuran (THF), acetonitrile (MeCN), dimethyl sulfoxide (DMSO), and propylene carbonate (PC). We found VBH solubilities up to 1.09 M and that, in general, solubility increases with increasing alkyl chain length, with notable exceptions (Table 1). Solubility is also affected by the oxidation state, with +4 states generally exhibiting greater solubility than their +5 counterparts, with notable variability in various solvents. Because experimentally exploring all possible combinations of alkyl substituents on the supporting cations and their interactions with solvents to identify highly soluble species empirically is an inefficient approach, our results suggest that a predictive theoretical framework could be beneficial to NRFB electrolyte development and optimization. This led us to explicitly explore the interplay between competing trends in solvation energy and lattice enthalpy, which was implied by our previous work,^5^ using a combined computational and synthetic strategy.

**Table 1:** Experimental solubilities of the [N_*xxxx*_]_*y*_[VBH] active materials in the +4 (where *y* = 2) and +5 (where *y* = 1) states in various solvents. Values correspond to the maximum concentration, in mol L^−1^, of VBH in the electrolyte as determined by UV-Vis spectroscopy

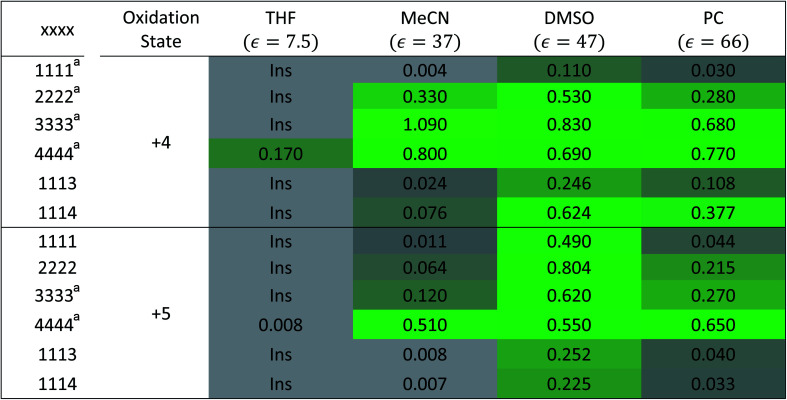

a Solubilities from ref. 5.

### 2.2 Alkyl ammonium cation substitution to explore steric and electrostatic effects on solubility

To explore other possible soluble alkylammonium cations, we hypothesized that short-chain, asymmetric alkylammonium cations would exhibit better solubility due to the destabilization of the crystal structure 
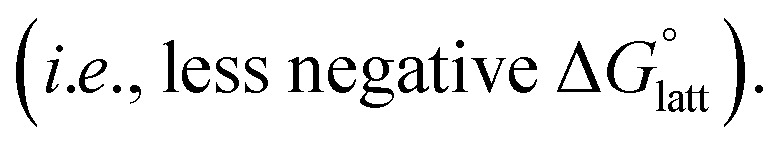
 We employed desymmetrization by substitutions on the smallest ([N_1111_]^+^) and largest ([N_4444_]^+^) cations that were used in experiment (Fig. 3a). At most two substitutions were performed in each set, generating groups of monosubstitutions, symmetric disubstitutions, and asymmetric disubstitutions. To probe the steric effect of alkyl chain length, we used ethyl (2), *n*-propyl (3), and *n*-butyl (4) for [N_1111_]^+^. For the larger cation, [N_4444_]^+^, the steric effects were also probed using a methyl (1) substituent and the relatively bulkier *sec*-butyl (*s*) and *tert*-butyl substituents (*t*). To investigate electrostatic effects, we modified the [N_4444_]^+^ with trifluoromethyl (*c*) substituent. Fig. 3e–h shows representative structures of the symmetric and desymmetrized [N_4444_]^+^.

### 2.3 Simplified crystal models

In order to calculate the solubility by first-principles, we first developed a simplified model for the crystal structures of interest. The unit cells of the crystallized active materials, resolved from X-ray diffraction, ranged from two (*Z* = 2) to 16 (*Z* = 16) formula units corresponding to system sizes of 126 and 736 atoms, respectively, which poses expensive computational cost. Solvent molecules were also observed in most crystal structures which adds complexity to the systems (see Table S1† for parameters). To reduce the complexity and the cost of the calculations, we simplified the systems into single formula unit cells (*Z* = 1) and monitored the response of the corresponding energetics of the system to this modification.

To benchmark our calculations, we compared the calculated molar volumes of the simplified crystals to the molar volumes of the experimentally crystallized active materials for [N_*xxxx*_]_*y*_[VBH] (where *x* = 1, 2, 3, 4 and *y* = 1, 2). Table S1† shows the experimental molar volumes, *V*_m_ (exp), and *V*_m_ (calc) of the simplified crystal structures. Despite the volume that the trapped solvent molecules occupy, the simplified models captured the local arrangement of the ions in the bulk crystal. This is especially true for the [N_2222_][VBH] and [N_3333_][VBH] crystals, which had no solvent impurities in the crystal lattice, where the calculated molar volumes differed only by 2.17% and 0.53%, respectively, from the actual molar volumes. The simplified V^4+^ crystals also performed well in replicating the actual molar volumes with the closest prediction obtained from [N_4444_]_2_[VBH] at 0.41% difference. This suggests that the crystal simplification approximation does capture local regions of the actual crystal structures with considerable accuracy.

We note that bulk morphological variation is neglected in this approach resulting in systematically high lattice energies, especially for crystals with large *Z*. Nevertheless, the simplified approach employed here, which is empirically calibrated by solubility measurements, provides a middle ground for both computational cost and accuracy. It is an excellent tool for exhaustive screening and exploration of materials to a depth that would not be possible by synthetic approaches. Table S7† lists the formula unit Cartesian coordinates for each of the simplified crystals in both +4 and +5 redox states.

### Lattice free energy 
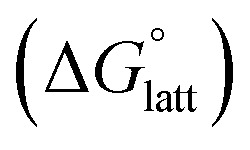
 and free energies of sublimation 
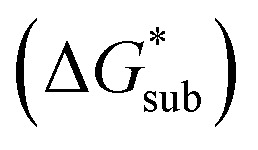


2.4

Sublimation of crystalline materials is an endergonic process under standard conditions 
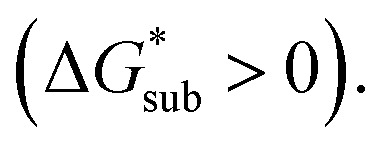
 As the magnitude of 
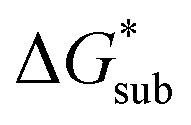
 increases, sublimation becomes less favorable leading to a more stable crystal. Eqn (1) implies that the greater the magnitude of 
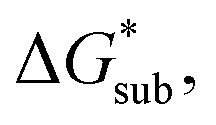
 the lower the solubility. Since 
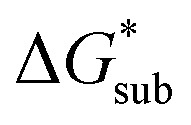
 is approximately the negative of the 
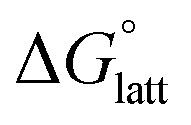
 (ESI eqn (2)†), a material would become more soluble as the 
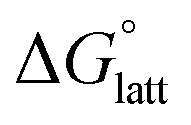
 becomes less negative.

**Fig. 4:**
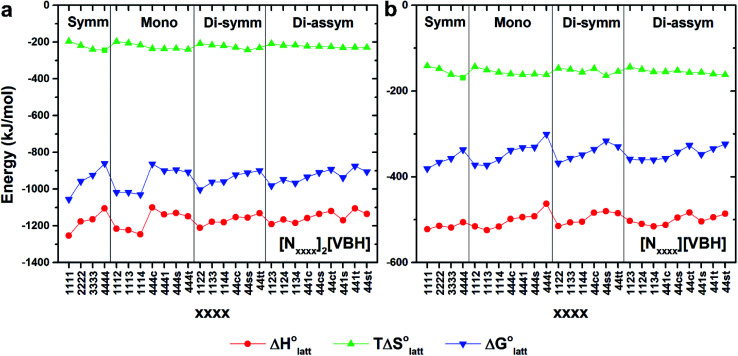
DFT calculated lattice energy profiles of the [N_*xxxx*_]_*y*_[VBH] active materials in the (a) +4 (where *y* = 2) and (b) +5 (where *y* = 1) states. Crystals are grouped into unsubstituted (Symm), mono-substituted (Mono), symmetric disubstituted (Di-symm), and asymmetric disubstituted (Di-asymm) cations and a group is arranged by increasing cation size.

Fig. 4a and b illustrate the performance of the models in predicting the trends of 
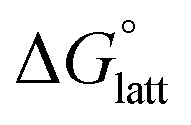
 (data shown in Table S3†). The first four points, corresponding to the four symmetric [N_*xxxx*_]_*y*_[VBH], show that as the cation size increases, 
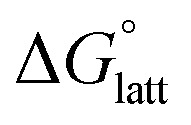
 becomes less negative. This result correlates with experimental solubilities (Table 1), where increasing the size of the cation destabilizes the crystal lattice and increases solubility. This experimental trend was not captured by 
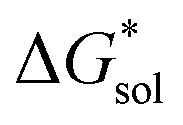
 (described in Section 2.5) demonstrating that the solubility of the VBH crystals is dictated more by the changes in the 
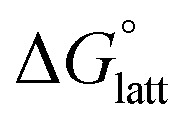
 than changes in the 
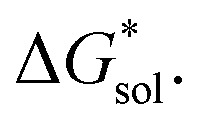


Within the +4 and +5 group, [N_1111_]_2_[VBH] and [N_1111_][VBH] crystals have the most negative 
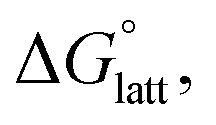
 with about 99–196 kJ mol^−1^ and 15–45 kJ mol^−1^ lower than the other symmetric compounds, respectively (Table S3†). This difference between the scale of the relative 
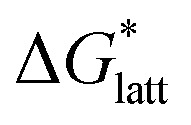
 as a response to the change of cation can be attributed mainly to the electrostatic potential of the V^4+^ and V^5+^ states. Increasing cation size destabilizes the coulombic interactions in the lattice by increasing ion separation and interactions with neighboring ions, which has a greater effect in [VBH]^2−^.

In general, both the alkyl chain length and bulkiness promoted lattice destabilization. However, because of the greater electrostatic potential of the dianionic [VBH]^2−^, the cation modifications on the V^4+^ group resulted in lower 
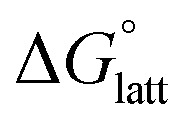
 than [N_4444_]_2_[VBH]. The closest were the monosubstituted [N_444c_]_2_[VBH] and the asymmetric disubstituted [N_441t_]_2_[VBH], which are only 2.9 and 15.0 kJ mol^−1^ more stable than [N_4444_]_2_[VBH], respectively (Table S3†). This can be attributed to the charge screening by the bulky *tert*-butyl and the electronegative trifluoromethyl groups. On the other hand, in the lower electrostatic potential of the [VBH]^−^ anion, there are more pronounced effects of charge screening and ion separation and several of the desymmetrized cations resulted in V^5+^ crystals with higher 
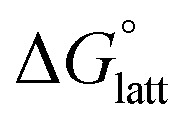
 than [N_4444_][VBH] (Table S3†) such as the monosubstituted [N_444t_]^+^, the symmetric disubstituted [N_44ss_]^+^, and the asymmetric disubstituted [N_44st_]^+^ and [N_44ct_]^+^.

### Free energies of solvation, 
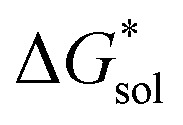


2.5

**Fig. 5:**
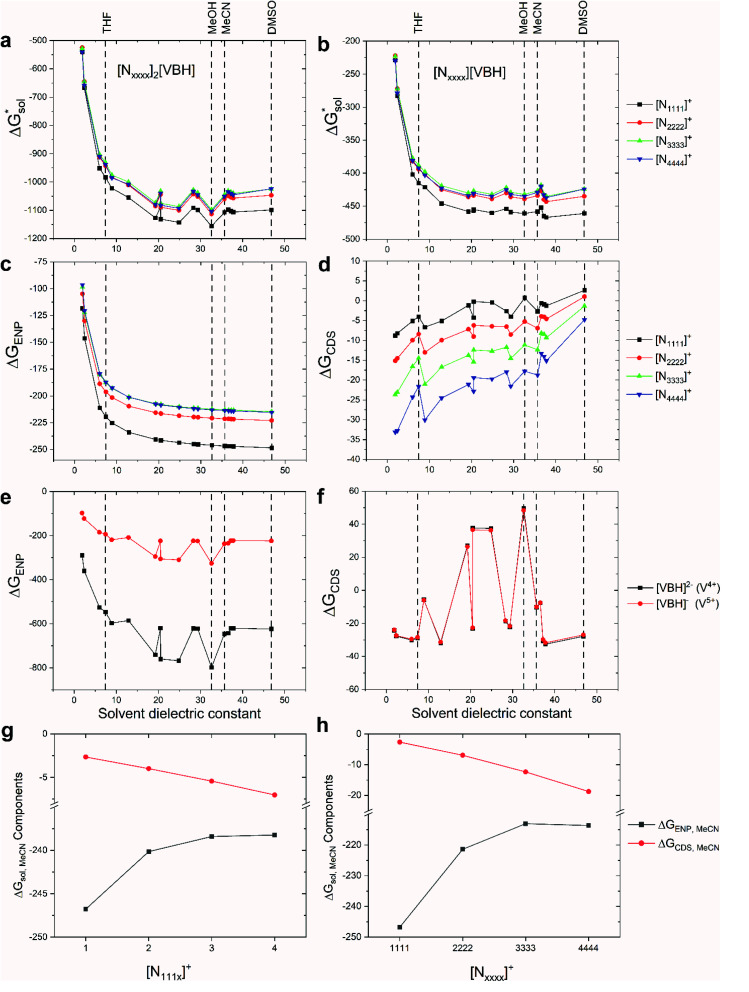
Solvation free energies, 
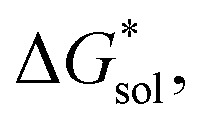
 of VBH with the symmetric cations in its (a) reduced and (b) oxidized states. Also shown are the comprising bulk-electrostatic, Δ*G*_ENP_, and non-electrostatic, Δ*G*_CDS_, components of the solvation energies for each ion: panels (c) and (d) for the Δ*G*_ENP_ and Δ*G*_CDS_ of symmetric cations, respectively, while panels (e) and (f) for the VBH anion in the +4 and +5 oxidation states. Panels (g) and (h) show the changes in the Δ*G*_ENP_ and Δ*G*_CDS_ in MeCN as the alkyl chain lengths are increased for [N_111*x*_]^+^ and [N_*xxxx*_]^+^ respectively. All energy values are in kJ mol^−1^ units.

To determine and quantify the role of 
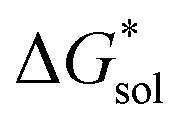
 on the solubility prediction and compare with the magnitude of 
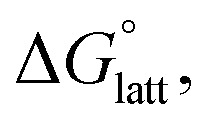
 we calculated the 
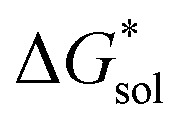
 of the symmetric and asymmetric VBH active materials in several solvents (Fig. 5 and Table 2, full list of solvents in Table S2,† and heat maps of V^4+^ and V^5+^ compounds in Tables S4 and S5†). Of the three solvents (THF, MeCN, and DMSO) that were used in the experimental solubilities, our calculations predicted that the solvent with the most favorable 
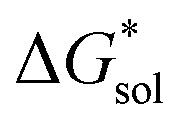
 for V^4+^ compounds is MeCN, while for V^5+^ compounds, it is DMSO. These are consistent with the general experimental solubility trends (*i.e.*, the solubilities for V^4+^ are highest in MeCN, while V^5+^ solubilities are highest in DMSO, Table 1). However, 
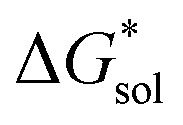
 alone could not explain the solubility trends with respect to the size of the alkylammonium chain. For example, for the four symmetric V^4+^ compounds in MeCN with increasing chain length ([N_1111_]_2_[VBH] to [N_4444_]_2_[VBH]), 
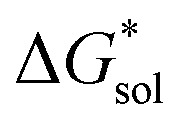
 were determined to be −1107, −1060, −1045, −1050 kJ mol^−1^ (Table 2), contrary to experimental solubilities of 0.004, 0.330, 1.090, and 0.800 (Table 1), respectively.

**Table 2:** PBE-D3BJ/def2-TZVP calculated solvation free energies 
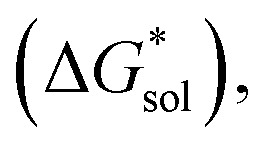
 in kJ mol^−1^, of the [N_*xxxx*_]_*y*_[VBH] active materials, in the +4 (where *y* = 2) and +5 (where *y* = 1) states, in tetrahydrofuran (THF), acetonitrile (MeCN), and dimethylsulfoxide (DMSO) SMD solvents

	Vanadium (+4) species			Vanadium (+5) species		
	THF	MeCN	DMSO	THF	MeCN	DMSO
**Symmetric**						
1111	−984.5	−1107.1	−1099.3	−414.9	−457.9	−461.1
2222	−943.3	−1060.0	−1046.6	−394.3	−434.4	−434.7
3333	−931.3	−1044.8	−1025.1	−388.3	−426.8	−424.0
4444	−938.0	−1050.0	−1024.5	−391.7	−429.4	−423.7

**Monosubstituted**						
1112	−973.3	−1094.3	−1085.1	−409.3	−451.5	−454.0
1113	−969.2	−1089.5	−1078.7	−407.2	−449.1	−450.8
1114	−969.4	−1089.2	−1077.0	−407.3	−449.0	−449.9

**Symmetric disubstituted**						
1122	−963.0	−1082.6	−1071.9	−404.2	−445.7	−447.4
1133	−956.1	−1074.2	−1060.4	−400.7	−441.5	−441.6
1144	−957.1	−1074.3	−1057.6	−401.2	−441.5	−440.2

**Asymmetric disubstituted**						
1123	−959.5	−1078.3	−1066.1	−402.4	−443.5	−444.5
1124	−959.8	−1078.2	−1064.5	−402.6	−443.5	−443.7
1134	−956.6	−1074.2	−1059.0	−400.9	−441.5	−440.9

**Monosubstituted**						
444c	−931.1	−1045.2	−1023.7	−388.2	−427.0	−423.3
4441	−946.1	−1060.6	−1039.5	−395.7	−434.7	−431.2
444s	−929.6	−1041.4	−1016.2	−387.5	−425.0	−419.6
444t	−926.8	−1038.3	−1014.2	−386.0	−423.5	−418.5

**Symmetric disubstituted**						
44cc	−914.4	−1029.2	−1011.4	−379.9	−419.0	−417.1
44ss	−921.1	−1032.4	−1008.1	−383.2	−420.6	−415.5
44tt	−914.5	−1025.6	−1002.6	−379.9	−417.2	−412.7

**Asymmetric disubstituted**						
441c	−945.6	−1063.2	−1046.1	−395.4	−436.0	−434.5
44cs	−921.5	−1035.1	−1013.8	−383.4	−421.9	−418.3
44ct	−919.8	−1033.2	−1012.8	−382.5	−421.0	−417.8
441s	−938.3	−1052.5	−1031.8	−391.8	−430.6	−427.3
441t	−935.9	−1050.1	−1030.1	−390.6	−429.4	−426.5
44st	−918.8	−1030.2	−1006.5	−382.1	−419.5	−414.7

To understand this better, within the implicit solvation model based on density (SMD) approach, we can decompose the 
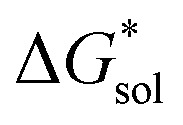
 further into two main contributions: (1) the Δ*G*_ENP_ and (2) Δ*G*_CDS_. The Δ*G*_ENP_ takes into account the bulk-electrostatics from the solute electronic kinetic and electronic-nuclear coulombic energies in the presence of the solvent (EN) and the solution polarization (*P*) free energy.^34,35^ The Δ*G*_CDS_ accounts for the non-electrostatic effects of cavitation (C, energy required to make room in the solvent for the solute), dispersion (D, change in dispersion energy upon dissolution), and changes in the solvent structure (S, energetic and entropic effects from structural changes in the solvent).^34,35^ By evaluating Δ*G*_ENP_ and Δ*G*_CDS_ separately, we can gain insights into the effects of the structural modifications of the cations to the 
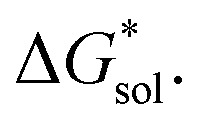


As mentioned earlier, in all solvents and for both +4 and +5 states considered, 
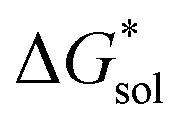
 increases (more positive) with cationic size (Table 2, Fig. 5a and b), indicating that solvation becomes less favorable. This could be attributed mainly to the reduced bulk-electrostatic interactions (Δ*G*_ENP_) as the cation size is increased (Fig. 5c). The smaller cations have more favorable bulk-electrostatic interactions (Fig. 5c). Electrostatic potential maps from natural population analysis charges reveal that the unit charge of the cations is distributed over the hydrogens of the carbon atoms adjacent to the nitrogen atom (Fig. S2†). In general, as the alkyl chain length increases, the solvent access towards these hydrogen atoms becomes more restricted, thus reducing bulk-electrostatic interactions. Interestingly, the [N_3333_]^+^ and [N_4444_]^+^ cations have similar Δ*G*_ENP_ (Fig. 5c) across all solvents, suggesting that the bulk-electrostatic penalty diminishes beyond three carbon atoms (*e.g.*, for [N_111*x*_]^+^ and [N_*xxxx*_]^+^ in MeCN, see Fig. 5g and h).

An inverse trend is observed for the Δ*G*_CDS_ of the symmetric cations (Fig. 5d) where increasing the cation size improves stabilization from non-electrostatic interactions. However, unlike the Δ*G*_ENP_, there is no apparent diminishing effect for the Δ*G*_CDS_ after three carbon atoms (see Fig. 5g and h for [N_111*x*_]^+^ and [N_*xxxx*_]^+^ in MeCN), and it progressively became more favorable as less polar solvents were used. Furthermore, there is a clear difference between the Δ*G*_CDS_ of [N_3333_]^+^ and [N_4444_]^+^ as the solvent polarity is decreased. Although the Δ*G*_CDS_ contribution is significantly smaller than Δ*G*_ENP_, in cations with roughly the same Δ*G*_ENP_, the Δ*G*_CDS_ can be the determining factor for a better 
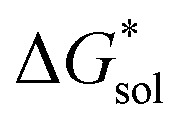
 such as in the case of [N_3333_]^+^ and [N_4444_]^+^ which leads to a more negative 
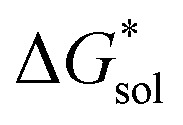
 for [N_4444_]_2_[VBH] and [N_4444_][VBH] compared to [N_3333_]_2_[VBH] and [N_3333_][VBH], respectively, in THF and MeCN. This also provides a rationale for the experimentally observed enhanced solubility of [N_4444_]_*y*=1,2_[VBH] in THF.

The same observation can be made for the modified [N_11*xx*_]^+^ (Fig. S1a and b†) as the symmetric ones with a similar diminishing Δ*G*_ENP_ penalty beyond three carbon atoms. For the [N_44*xx*_]^+^ modifications (Fig. S1c and d†), the use of the bulkier *sec*- and *tert*-butyl appears to restrict solvent access further, leading to less favorable 
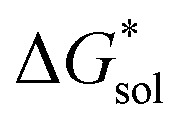
 when compared to the symmetric [N_4444_]^+^. In contrast, the smaller methyl substituent allowed for better solvent access, which resulted in the greatest improvement, followed by the –CF_3_, of 
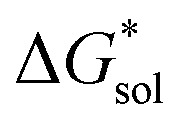
 in the [N_44*xx*_]^+^ group.

The effect of hydrogen bonding interactions between the VBH anions and protic solvents significantly improved the Δ*G*_ENP_ (Fig. 5e). As expected, the response of Δ*G*_ENP_ towards changing the solvent is similar for both [VBH]^2−^ and [VBH]^−^, which differed only in the scale due to the charge difference. Also, preference towards MeCN over DMSO is observed for the VBH anions and is due to the slight acidity of the hydrogen atoms of MeCN (Abraham's hydrogen bond acidity, *α*_MeCN_ = 0.07) despite being classified as aprotic. Almost identical values of Δ*G*_CDS_ (Fig. 5f) were observed for both oxidation states of the VBH anion. While solvents with acidic protons should be considered for NRFB applications only with caution, since they would be expected to affect the electrochemical stability windows negatively, this is nevertheless an interesting observation and an important consideration in optimizing the solvent to be used in an electrolyte formulation.

### 2.6 Calculated solubilities

**Fig. 6:**
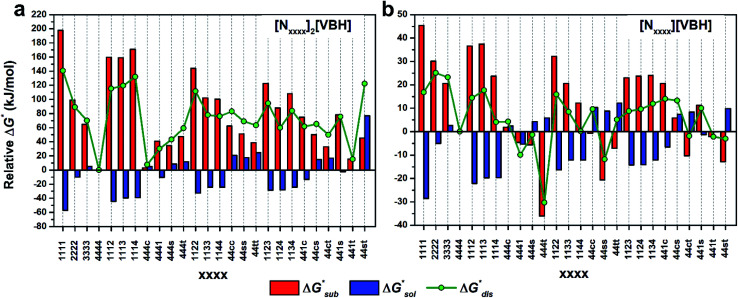
Free energies of sublimation 
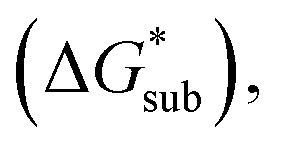
 solvation 
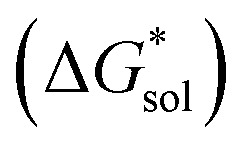
 in MeCN, and dissolution 
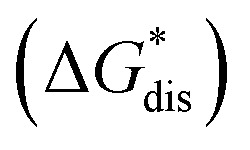
 in MeCN of the [N_*xxxx*_]_*y*_[VBH] active materials in the (a) +4 (where *y* = 2) and (b) +5 (where *y* = 1) states relative to the free energies of [N_4444_]_2_[VBH] and [N_4444_][VBH], respectively.

To compare relative solubilities, we calculated the free energies of dissolution 
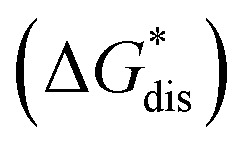
 in MeCN of each crystal relative to the most soluble in both oxidation states, [N_4444_]_2_[VBH] and [N_4444_][VBH]. This approach eliminated the numerical inconsistencies from the different computational methods and approximations used to treat the bulk crystalline and solvated systems, outlined in the Methods and ESI sections,† highlighting the differences attributed only to the cation composition and solvent used. The resulting solubility values can then be interpreted as either greater than or less than the solubility of [N_4444_]_*x*=1,2_[VBH] in MeCN. Fig. 6 shows the relative 
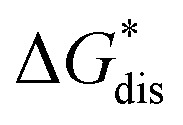
 in MeCN for the V^4+^ and V^5+^ crystals along with the components it comprises, 
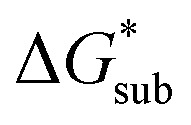
 and 
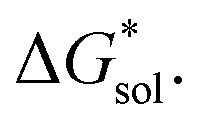
 Our results show that 
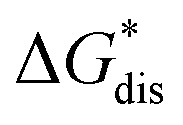
 is predominantly governed by the 
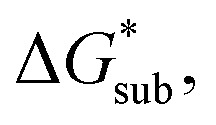
 in agreement with our experimental observation where [N_1111_]_*y*=1,2_[VBH] exhibited the least solubility despite having the most favorable 
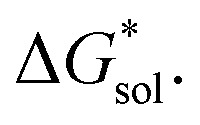


These results support that the models and methodology employed can reliably capture solubility trends and can be an efficient tool in tuning the thermodynamics useful for solubility improvements. The observed modest increase in solubility for the desymmetrized cations, [N_1113_]^+^ and [N_1114_]^+^ (Table 1) was also captured by the simplified models and can be explained by a modest reduction of the corresponding 
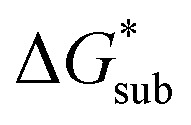
 compared to [N_1111_]^+^. We note that in the [N_1111_][VBH] crystal, the actual packing involved 16 formula units (*Z* = 16) and that stability from this quasi-amorphous packing is not captured by the simplified model, hence the underestimation of the calculated 
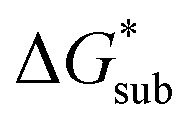
 for [N_1111_][VBH] and possibly for [N_1113_][VBH] and [N_1114_][VBH]. Nevertheless, these simplified models can provide qualitatively accurate 
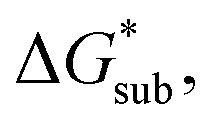
 albeit systematically higher, approximations for crystals with smaller *Z*.

**Fig. 7:**
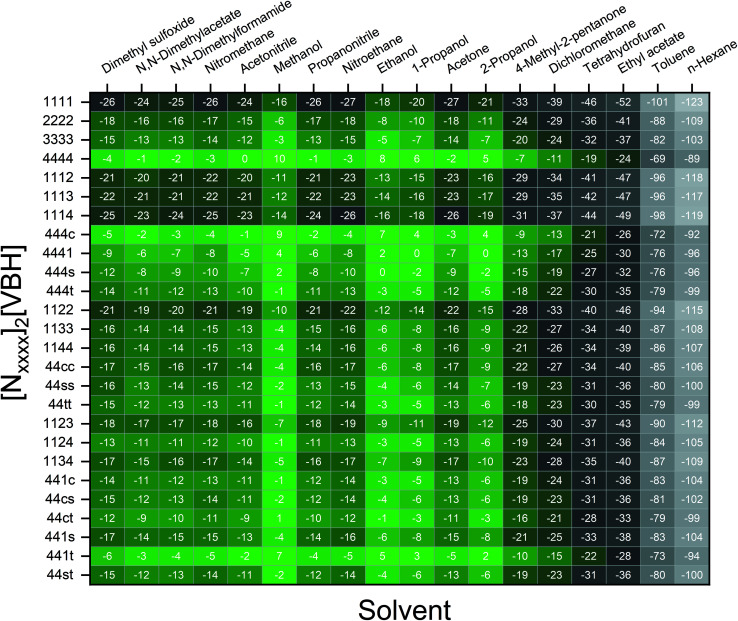
Heat map of log(*S*_0_) for [N_*xxxx*_]_2_[VBH] in different solvents. Solubilities were taken relative to the solubility of [N_4444_]_2_[VBH] in MeCN. Green cells indicate better or comparable solubilities with respect to [N_4444_]_2_[VBH] in MeCN.

**Fig. 8:**
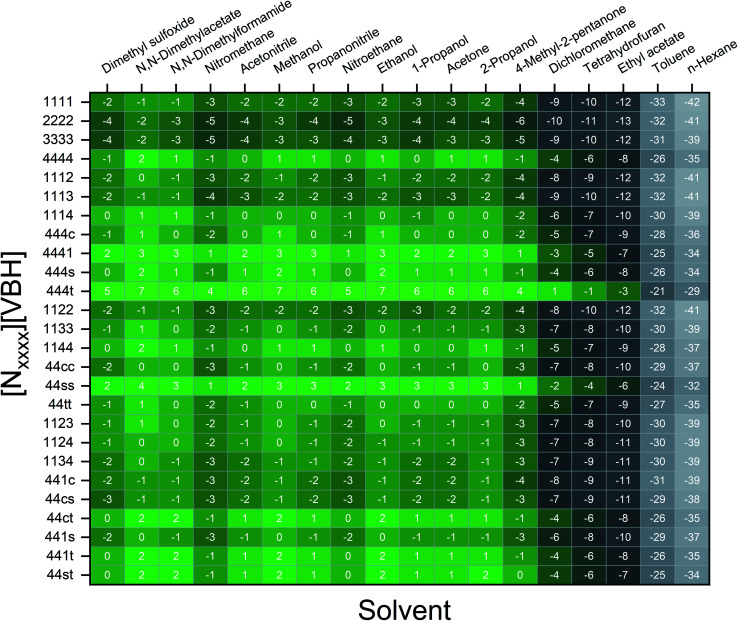
Heat map of log(*S*_0_) for [N_*xxxx*_][VBH] in different solvents. Solubilities were taken relative to the solubility of [N_4444_][VBH] in MeCN. Green cells indicate better or comparable solubilities with respect to [N_4444_][VBH] in MeCN.

Fig. 7 and 8 show heat maps of the calculated solubilities, in the logarithmic scale, of the crystals in the V^4+^ and V^5+^ states, respectively, using different solvents. In these figures, the greener a cell is, the more soluble that particular crystal is in the corresponding solvent relative to the solubility of [N_4444_]_2_[VBH] or [N_4444_][VBH] in MeCN. Among the branched and straight-chain, symmetric and asymmetric alkylammonium VBH compounds we investigated, in the V^4+^ state, the [N_4444_]^+^ cation is the most soluble, followed closely by [N_444c_]^+^ and [N_441t_]^+^. In the V^5+^ state, the use of the cations [N_444t_]^+^, [N_44ss_]^+^, [N_44st_]^+^, or [N_44ct_]^+^ showed better solubilities than [N_4444_][VBH] and will be the focus of synthetic efforts.

### 2.7 Solvent proticity and viscosity considerations

The choice of solvent is a crucial aspect of NRFBs as it affects not only its chemical but also its operational and physical properties. Our 
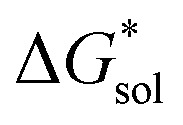
 results suggest that alcohols could improve the solubilities of both V^4+^ and V^5+^ crystals. The more polar solvents *N*,*N*-dimethylacetate and *N*,*N*-dimethylformamide, and the less polar propanonitrile, are also promising solvents for the V^5+^ crystals (Fig. 8). Although alcohols appear to be promising solvents based on solubility, their protic nature could pose unwanted degradation reactions in prolonged cycling. Their affinity towards absorbing atmospheric moisture could also prove to be problematic in longer operations.^36^

**Fig. 9:**
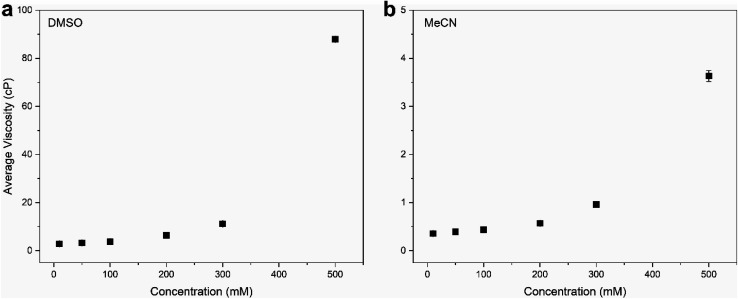
Viscosity measurements of [N_4444_]_2_[VBH] in (a) DMSO and (b) MeCN at 0.01 M, 0.05 M, 0.1 M, 0.2 M, and 0.5 M concentrations. All experiments were performed using a viscometer at room temperature (25 °C).

To demonstrate the viscosity behavior of VBH electrolytes, we measured the viscosity of [N_4444_]_2_[VBH] in DMSO and MeCN at 0.01 M, 0.05 M, 0.1 M, 0.2 M, and 0.5 M concentrations (Fig. 9). Our results show that viscosity generally increases with solution concentration. Moreover, the viscosity of DMSO electrolyte depends more steeply on concentration than the MeCN electrolyte, which could considerably impede the flow performance at the concentrations that we wanted to achieve with increased pumping power requirements and slower reaction kinetics. Our findings demonstrate the vital role of the viscosity of the electrolyte solution in choosing the appropriate solvent. A rough approximation of the resulting viscosities can be obtained from the kinematic viscosities (Table S2†) of all the solvents used in the calculations. Although we predicted that *N*,*N*-dimethylacetate, *N*,*N*-dimethylformamide, and propanonitrile are favorable solvents from the perspective of active-material solubility, these solvents' viscosities are significantly higher than that of MeCN, which will likely hinder their practical applications.

## 3. Conclusion

We demonstrated a first-ever, first-principles prediction of solubilities of nonaqueous flow-battery active-materials, incorporating both the lattice and solvation energies. The computational predictions were compared and validated with experimental data. We found that the solvation energy alone is insufficient and can even be misleading as a tuning parameter to improve solubility, especially in ionic systems. Lattice energy has a more significant effect on solubility. The desymmetrization of the alkylammonium cation leading to short-chain, asymmetric cations demonstrated a modest increase in solubility, which can be further explored for NRFB electrolyte development and optimization. Based on the framework developed herein, investigations on structural modification of the active-material, with the goal of synergistic improvements to solubility, are on-going. While changes in the reduction potential arising from varying the counter ion are expected to be minimal, based on previous investigations,^37^ we believe that tuning the structure of the active-material will allow simultaneous improvements to solubility and redox properties. Our findings demonstrated the critical role of the viscosity of the electrolyte solution in choosing the appropriate solvent.

Design strategies should take into account the solid-state energetics. In our efforts to find VBH compounds with high solubility, we found that, while desymmetrization of the cation can be important, it does not always lead to a more soluble active material. One design strategy for improving VBH solubility using alkyl ammonium cations is by increasing the alkyl chain lengths, which is favorable with respect to lattice free energy, but unfavorable with respect to solvation free energy because of the steric hindrance imparted by the larger cations. In this, and in previous work,^5^ we demonstrate that the former effect outweighs the latter, giving rise to the overall improvement to solubility observed for longer alkyl-chain cations. With the computational investigations reported herein, we elaborate that the electrostatic interaction between the cations and the solvent becomes less effective as the alkyl chain length is increased up until 3-carbon atoms, then plateaus. This implies that, as a design strategy for improving solubility, the use of alkyl ammonium cations with chain-lengths of 4-carbon atoms, or more, should exhibit a reduced lattice free energy with a decreasingly significant penalty to solvation free energy. Another design principle for improving VBH solubility is the use of bulky substituents. This effect is particularly pronounced in the V^5+^ crystals where lattice electrostatic interactions are lower than in the V^4+^ crystals. Compared to their straight-chain counterparts, *sec*- and *tert*-butyl substituents showed appreciable reduction of 
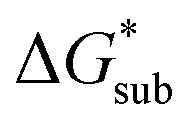
 and slight increase in 
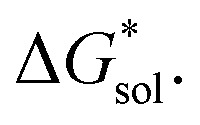
 Nevertheless, these responses from bulky substituents, together with alkyl chain length, are promising, and will be explored in our future efforts.

This work is an in-road toward VBH active materials with very high solubility and potentially those that are liquid at operating temperature. If viscosity concerns could be mitigated, the production of such liquid active materials would significantly increase energy density and could eliminate the need for supporting electrolytes. Furthermore, the establishment of this theoretical framework, coupled with experimental verifications, opens avenues for machine learning models and other computational screens of solution properties (*e.g.*, diffusion rates, viscosities, and ionic conductivities), both of which are parts of our in-road strategy for designing high energy density NRFB active materials. Because the methods involved in this computational protocol are available in most computational chemistry programs, this strategy can be applied to any RFB chemistry including organic aqueous systems, with the cost and complexity of such calculations largely depending on the composition and size of the system.

## 4. Methodology

### 4.1 Computational

The intrinsic solubility (*S*_0_) of a substance is directly related to the difference in its stabilities in both the solid and solvated states. This relationship is demonstrated from the well-known Born–Fajans–Haber thermochemical correlation^26^ (Fig. 2), which translates to1
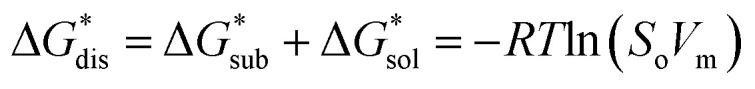
where 
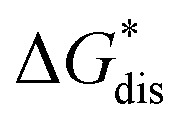
 is the free energy of dissolution and is the sum of the free energies of sublimation 
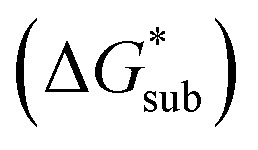
 and solvation 
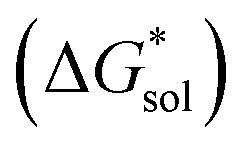
 while *R*, *T*, and *V*_m_ are the gas constant, temperature, and the solid's molar volume, respectively. In this cycle, a substance first sublimes into the gas phase, then gets solvated into the solution phase and the free energies associated with each process determine its dissolution. The free energy of sublimation is roughly the negative of the lattice free energy 
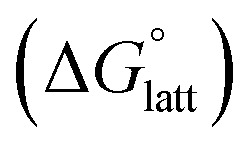
. The details of the theory and corresponding equations are given in ESI†.^30–32,38–40^

Starting geometries for single formula unit crystals of the systems with symmetric cations ([N_1111_]^+^, [N_2222_]^+^, [N_3333_]^+^, and [N_4444_]^+^) were extracted from the X-ray crystal structures for both reduced and oxidized states. Five of these have been reported previously^5,22,23^ and two are newly reported herein (Fig. 3 and Table S6†). These crystals were then equilibrated using the method discussed below. The resulting optimized crystal structures of the [N_1111_]^+^ and [N_4444_]^+^ systems were then subjected to cation modification and re-optimization.

Plane-wave-based periodic density functional theory (DFT) calculations for the sublimation free energies were performed using Quantum Espresso v6.5 (QE).^41,42^ The valence electronic states were expanded based on plane waves, and the core–valence interaction was described using the ultrasoft pseudopotential approach. The Perdew–Burke–Ernzerhof (PBE)^43^ generalized gradient approximation functional with a 1225 eV and 9796 eV basis set cutoffs for the plane wave kinetic energy and the electron density, respectively, were used. Spin-polarization was used for the open-shell systems (V^4+^ crystals; d^1^), while non-polarized calculations were done for the closed-shell systems (V^5+^ crystals; d^0^). For the bulk calculations, the Brillouin zone was sampled using a Monkhorst–Pack k-point mesh of 2 × 2 × 2 with grid offsets, resulting in less than 0.0001 eV change in the total energy compared to 3 × 3 × 3. The two-body dispersion interactions in the crystals were included using Grimme's DFT-D2 (ref. 44 and 45) method implemented in QE. The atomic coordinates and cell parameters were fully optimized to a 1 × 10^−5^ eV and a 1 × 10^−5^ eV Å^−1^ force threshold. Given the size of the unit cells (containing 46 to 135 atoms), the phonon frequencies were only calculated at the Gamma point.^33,46^

The free energy of solvation 
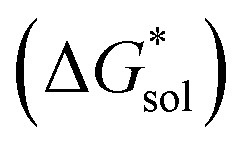
^47,48^ was obtained using the implicit solvation model based on density (SMD) method^34^ as implemented in Orca v4.2.1.^49^ Each ion was first optimized in the gas phase using the PBE functional and Grimme's DFT-D3 (ref. 50) dispersion correction with the Becke–Johnson damping (D3BJ)^51^ with a def2-TZVP^52^ basis set. SMD was then employed on the gas-phase structures to obtain the standard free energy of solvation.^47,53,54^ A list of battery-relevant solvents used in this study is listed in Table S2.†

### 4.2 Experimental

#### Physical methods

UV-Vis spectra were collected using the Evolution 220 UV-visible spectrophotometer with a quartz cuvette of 1 cm path length. The molar extinction coefficients for oxidized and reduced species in all solvents except for DMSO were used as determined in our previous study.^5^ Molar extinction coefficients for oxidized species in DMSO were determined from a calibration curve. Infrared spectra were collected on a Thermo Scientific Nicolet iS5 equipped with iD7ATR module and a diamond crystal. ^1^H- and ^13^C-NMRs were performed on Bruker AVANCE III HD 400 MHz High-Performance Digital NMR spectrometer operating at 400 MHz for ^1^H NMR and 101 MHz for ^13^C NMR. Data acquisition was performed on IconNMR 5.0.3, and spectra were processed in TopSpin 3.5 and Mnova. High-Resolution Mass Spectrometry (HRMS) was performed on a Waters ACQUITY UPLC Xevo QTOF high resolution mass spectrometer using electrospray ionization. X-ray crystallographic experiments were performed on a Bruker D8 Venture X-instrument, using Mo Kα radiation at 200 K. Data were corrected for absorption using SADABS. The structures were solved by direct methods. All non-hydrogen atoms were refined anisotropically by full matrix least-squares on *F*^2^ and all hydrogen atoms except those on water were placed in calculated positions with appropriate riding parameters. Further refinement and molecular graphics were obtained using Bruker Suite of structural programs,^55^ OLEX2,^56^ and Mercury.^57^

#### General

Hydroxylamine hydrochloride (Alfa Aesar), sodium hydroxide (Acros organics), chloroacetic acid (BTC), zinc acetate dihydrate (Acros Organics), calcium chloride (VWR), tetramethylammonium fluoride tetrahydrate (Matrix Scientific), tetraethylammonium fluoride (BTC), triethylamine (Acros Organics), *n*-butylbromide (Alfa Aesar), *n*-propylbromide (BTC), ferrocenium hexafluorophosphate (Sigma Aldrich), and vanadyl (iv) acetylacetonate (BTC) were purchased from commercial sources and used as received. Information on the characterization of new materials and those that were reported previously, and were used for solubility studies herein, are provided below. Solvent used in solubility measurement were of anhydrous grade and purchased from Sigma Aldrich.

New compounds, [N_1111_][VBH], [N_2222_][VBH], [N_1113_]_2_[VBH], [N_1113_][VBH], [N_1114_]_2_[VBH], and [N_1114_][VBH] were verified by comparison of infrared and UV-vis spectra with closely related compounds that have been published previously.^5,22,23^ Furthermore, the molecular ions in positive and negative polarity were verified to match calculated values. In addition, the newly reported V^5+^ compounds were verified with ^1^H NMR, by integrating the methylene and methyl protons of alkylammonium cations and comparing with the methylene protons of [VBH], in manner similar to that reported earlier.^5^ Finally crystal structures of the new compounds [N_1111_][VBH], [N_2222_][VBH], and [N_1114_]_2_[VBH], are presented herein.

#### Synthetic methods

##### Zinc hydroxyiminodiacetate (ZnHIDA) ligand synthesis

Zinc hydroxyiminodiacetate (ZnHIDA) was synthesized utilizing a method we previously reported.^5^ Hydroxylamine hydrochloride (34.7 g, 0.500 mol) was neutralized with 5 M NaOH (100 mL, 0.500 mol) in an ice bath maintaining the temperature of the reaction mixture between 0–4 °C. In another flask, chloroacetic acid (94.5 g, 1.00 mol) was neutralized by dropwise addition of 5 M NaOH (200 mL, 1.00 mol) in an ice bath. The neutralized mixture of chloroacetic acid was then added dropwise to hydroxylamine solution. At the end, additional 200 mL 5 M NaOH was added dropwise to the reaction mixture and the solution was stirred for 72 h in an ice bath. Then, the pH of the mixture was brought to 4.01 by acidifying with 6 M HCl to which zinc acetate dihydrate (110 g, 0.500 mol) was added to the solution while stirring. Upon addition, the ligand precipitated immediately as a zinc salt. A white precipitate of ZnHIDA was then filtered, washed with cold water several times, and dried *in vacuo*. (yield: 89.0 g, 0.420 mol, 84%). IR (*ν*/cm^−1^): 3228 (w), 2930 (C–H, w), 1648 (m), 1576 (C=O, s).

##### Calcium(ii)vanadium(iv)-bis-hydroxyiminodiacetate (CaVBH)

Calcium(ii)vanadium(iv)-bis-hydroiminodiacetate (CaVBH) was synthesized using a method previously reported.^5^ A suspension of ZnHIDA (89.0 g, 0.420 mol) in 500 mL deionized water was prepared, to which vanadyl(iv)acetylacetonate (55.7 g, 0.210 mol) was added and mixed well using a magnetic stir bar. Hydrochloric acid (80 mL, 6.0 M) was added dropwise to the stirring mixture. Calcium chloride dihydrate (30.8 g, 0.500 mol) was added to the solution and dissolved. 2-Propanol (2000 mL) was added to the resulting blue solution and stirred vigorously to facilitate the precipitation of CaVBH. The product was filtered and washed with 20 mL isopropanol followed by acetone and dried *in vacuo* for 24 h at room temperature (Yield: 90.0 g, 0.190 mol, 90%). UV-Vis (DMSO): *λ*_ma*x*_ = 578 nm (*ε* = 27.5 mol^−1^ cm^−1^). IR (*ν*/cm^−1^): 3541 (w), 3321 (w), 2988 (w), 1589 (C=O, s).

##### Tetramethylammonium vanadium(iv)-bis-hydroxyiminodiacetate [N_1111_]_2_VBH (1)

CaVBH (7.15 g, 0.0151 mol) was added to a stirring mixture of tetramethylammonium fluoride tetrahydrate (5.00 g, 0.0300 mol) in 40 mL ethanol. After 3 h of stirring, the solution was filtered to remove calcium fluoride and unreacted CaVBH. Tetrahydrofuran (15 mL) was added to the filtrate and cooled in the refrigerator. Crystals were obtained after 24 h in the refrigerator, which was then isolated *via* vacuum filtration and subsequently dried *in vacuo* for 24 h at room temperature (yield: 6.80 g, 88%). UV-Vis (DMSO): *λ*_ma*x*_ = 579 nm (*ε* = 30.2 mol^−1^ cm^−1^). IR (*ν*/cm^−1^): 3345 (w), 3031 (w), 2962 (C–H, w), 1619 (C=O, s), 1492 (C–N, w). HR-MS (ESI, positive ion mode) *m*/*z* calculated for C_4_H_12_N^+^ [M]^+^: 74.0964, found: 74.0954. MS (ESI, negative ion mode) *m*/*z* calculated for VBH^−^ [M]^−^: 342.9624, found: 343.0712.

##### Tetraethylammonium vanadium(iv)-bis-hydroxyiminodiacetate [N_2222_]_2_VBH (2)

CaVBH (4.00 g, 0.00840 mol) was added to a stirring mixture of tetraethylammonium fluoride tetrahydrate (3.05 g, 0.0185 mol) in 35 mL ethanol. After 2 h of stirring, the solution was centrifuged at 2000 rpm for 25 min. The liquid layer was isolated and concentrated using a rotatory evaporator at 40 °C. Crystals were obtained after 24 h in the refrigerator, which was then isolated *via* vacuum filtration and subsequently dried *in vacuo* for 24 h at room temperature (yield: 3.37 g, 0.00601 mol, 66%). UV-Vis (DMSO): *λ*_max_ = 578 nm (*ε* = 28.2 mol^−1^ cm^−1^). IR (*ν*/cm^−1^): 3506 (w), 2994 (w), 2955 (w), 2909 (w), 1602 (s). HR-MS (ESI, positive ion mode) *m*/*z* calculated for C_8_H_20_N^+^ [M]^+^: 130.1590, found: 130.1596. MS (ESI, negative ion mode) *m*/*z* calculated for VBH^−^ [M]^−^: 342.9624, found: 343.0635.

##### General procedure for preparation of asymmetric alkylammonium VBH

**Scheme 1:**
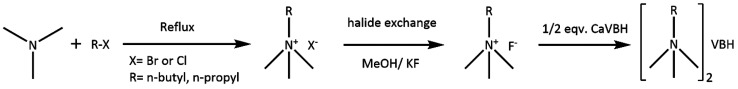
Synthesis of asymmetric quaternary ammonium VBH.

First, asymmetric quaternary ammonium bromides were prepared using Menshutkin reaction^58^ (Scheme 1). Thus prepared quat-bromides were converted to fluoride salts using halogen exchange reaction as described in the literature.^59^ Fluoride salts prepared *in situ* were directly reacted with CaVBH to afford corresponding quaternary ammonium VBH, as shown in reaction Scheme 1.

##### Trimethylpropylammonium bromide [N_1113_]Br

In a round bottom flask containing 50 mL ethanol, trimethylamine (59.1 g, 50 wt% aqueous solution, 0.500 mol) and 1-bromopropane (30.8 g, 0.250 mol) were added. The mixture was refluxed at 40 °C for 12 h under N_2_ atmosphere. The solvent was removed using a rotatory evaporator. The resulting solid was repeatedly washed with ethyl acetate and diethyl ether and dried in Schlenk line (yield: 31.0 g, 0.170 mol, 68%). *δ*_H_ (400 MHz, D_2_O) 3.26 (2H, t, *J* = 8.5 Hz), 3.09 (9H, s), 1.77 (2H, h, *J* = 7.4 Hz), 0.94 (3H, t, *J* = 7.3 Hz). *δ*_C_ (101 MHz, D_2_O) 68.12, 52.99, 16.12, 9.94.

##### Trimethylpropylammonium vanadium(iv)-bis-hydroxyiminodiacetate [N_1113_]_2_VBH (3)

Trimethylpropylammonium bromide (8.66 g, 0.047 mol) was dissolved in 25 mL dry methanol. Potassium fluoride (3.43 g, 1.3 equiv., 0.0590 mol) and deionized water (0.135 mL, 4 wt% of KF) was added to the solution. The mixture was vigorously stirred for 1 h and filtered to remove the solid. Potassium fluoride was added, stirred, and filtered two more times. Finally, the filtrate was concentrated, and any solid that appeared was filtered again. The yellowish oil (trimethylpropylammonium fluoride) was dissolved in 10 mL acetonitrile to which CaVBH (6.80 g, 0.0144 mol) was added and stirred for 30 min. The mixture was centrifuged, and the blue solution layer was concentrated under reduced pressure. Addition of diethyl ether afforded blue crystals of the title compound (yield: 7.00 g, 0.013 mol, 93%). UV-Vis (MeCN): *λ*_ma*x*_ = 579 nm (*ε* = 27.6 mol^−1^ cm^−1^). IR (*ν*/cm^−1^): 3448 (b), 3032 (w), 2970 (w), 1606 (s), 1494 (m), 1374 (m), 1326 (w), 1250 (w), 1124 (m), 966 (w), 916 (m), 945 (w), 889 (w), 722 (w), 613 (m), 536 (m). HR-MS (ESI, positive ion mode) *m*/*z* calculated for C_6_H_16_N^+^ [M]^+^: 102.1277, found: 102.1289. MS (ESI, negative ion mode) *m*/*z* calculated for VBH^−^ [M]^−^: 342.9624, found: 343.0721.

##### Butyltrimethylammonium bromide [N_1114_]Br

In a round bottom flask containing a mixture of 100 mL acetonitrile and 1-bromobutane (124 g, 0.906 mol), trimethylamine (50 wt% aqueous, 85.8 g, 0.725 mol) was added. The mixture was refluxed at 70 °C for 16 h under N_2_ atmosphere. The solvent was removed using a rotatory evaporator. The resulting solid was repeatedly washed with ethyl acetate and diethyl ether and dried in Schlenk line (yield: 87.2 g, 61%). *δ*_H_ (400 MHz, CDCl_3_) 3.62 (2H, t, *J* = 8.4 Hz), 3.47 (9H, s), 1.76 (2H, p, *J* = 7.7 Hz), 1.45 (2H, h, *J* = 7.4 Hz), 1.01 (1H, t, *J* = 7 Hz). *δ*_C_ (101 MHz, CDCl_3_) 66.49, 53.34, 24.92, 19.44, 13.66.

##### Trimethylbutylammonium vanadium(iv)-bis-hydroxyiminodiacetate [N_1114_]_2_VBH (4)

Butyltrimethylammonium bromide (7.16 g, 0.0365 mol) was dissolved in 20 mL methanol. To the solution, potassium fluoride (2.65 g, 0.0511 mol) and 0.105 mL deionized water (4 wt% of KF) was added, stirred for an hour, and filtered to separate the solid. The same amount of potassium fluoride was added to the filtrate and stirred for 1 h before filtering out the solid. The process was repeated one more time. Finally, the filtrate was concentrated to 10 mL using a stream of nitrogen gas. The solution was filtered again to remove any solid precipitated. The brownish-yellow oil ([N_1114_]F·*x*H2O) was dissolved in 10 mL acetonitrile to which CaVBH (4.75 g, 0.0100 mol) was added and stirred for 2 h. The mixture was centrifuged, and the liquid layer was concentrated under reduced pressure. Diethyl ether was added to aid precipitation. Blue solid was filtered and dried *in vacuo*. (Yield 5.20 g, 0.00904 mol, 90%) single crystal suitable for X-ray diffraction was prepared by layering diethyl ether into a solution of compound in propylene carbonate. UV-Vis (propylene carbonate): *λ*_ma*x*_ = 579 nm (*ε* = 27.5 mol^−1^ cm^−1^). IR (*ν*/cm^−1^): 3444 (w), 3029 (w), 2961 (w), 1606 (s), 1493 (w), 1378 (m), 1246 (w), 1126 (m), 917 (m), 613 (m), 592 (w) 526 (m). HR-MS (ESI, positive ion mode) *m*/*z* calculated for C_7_H_18_N^+^ [M]^+^: 116.1434, found: 116.1429. MS (ESI, negative ion mode) *m*/*z* calculated for VBH^−^ [M]^−^: 342.9624, found: 343.0546.

##### Synthesis of active material in their V(v) state *via* chemical oxidation

**Scheme 2:**
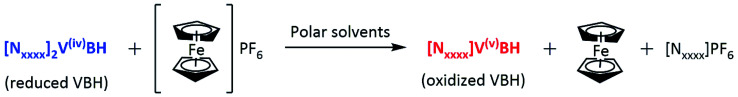
Synthesis of active materials *via* chemical oxidation.

All the active materials in V(v) state except [N_1114_]VBH were synthesized by general procedure of chemical oxidation method using one electron mild oxidizing agent, ferrocenium hexafluorophosphate (FcPF_6_). In general, the active material in V(iv) state was reacted with FcPF_6_ in 1 : 1 molar ratio in a polar solvent such as methanol, acetonitrile, or DMSO, as shown in Scheme 2. Then, the volume was doubled by adding diethyl ether to aid the precipitation of the product. The red crystalline powder obtained was repeatedly washed with diethyl ether to remove traces of ferrocene. The product obtained was characterized using ^1^H NMR, ^13^C NMR, FTIR, UV-VIS spectroscopy, and HR-MS. Removal of ferrocene and tetrabutylammonium hexafluorophosphate was confirmed by ^1^HNMR. Details of each preparation are described below.

##### Tetramethylammonium vanadium(v)-bis-hydroxyiminodiacetate [N_1111_]VBH (5)

Compound 1 (0.537 g, 0.00106 mol) was dissolved in 1 : 1 methanol/dmso to make 5 mL solution. FcPF_6_ (0.349 g, 0.00106 mol) was added to the solution and stirred for 10 min after which 20 mL diethyl ether was added to facilitate precipitation. Red powder was filtered and washed with diethyl ether until trace of ferrocene was removed. The product was dried *in vacuo* (yield: 0.39 g, 88%). UV-Vis (MeCN): *λ*_max_ = 495 nm (*ε* = 242 mol^−1^ cm^−1^). IR (*ν*/cm^−1^): 2978 (w), 2934 (w), 1663 (s), 1486 (m), 1360 (m), 1330 (w), 1257 (m), 1242 (m), 1153 (m), 1010 (w), 951 (m), 915 (s), 890 (m), 722 (w), 626 (s), 595 (m), 539 (w). *δ*_H_ (400 MHz, CD_3_CN) 4.95 (2H, d, *J* 16.1), 4.68 (2H, d, *J* 16.1), 4.59 (2H, d, *J* 16.8), 4.42 (2H, d, *J* 16.1), 3.07 (12H, s). *δ*_C_ (101 MHz, DMSO) 170.78, 170.54, 65.42, 64.55, 54.93. HR-MS (ESI, positive ion mode) *m*/*z* calculated for C_4_H_12_N^+^ [M]^+^: 74.0964, found: 74.0949. MS (ESI, negative ion mode) *m*/*z* calculated for VBH^−^ [M]^−^: 342.9624, found: 343.0705.

##### Tetraethylammonium vanadium(v)-bis-hydroxyiminodiacetate [N_2222_]VBH (6)

Compound 2 (1.3 g, 0.00216 mol) was dissolved in 13 mL MeCN. FcPF_6_ (0.727 g, 0.00219 mol) was added to stirring solution. The mixture was stirred for 10 min and centrifuged. The precipitate was formed upon addition of 10 mL diethyl ether which was then collected by filtration. Red solid was washed repeatedly with diethyl ether and dried *in vacuo*. Product was recrystallized in acetonitrile. Crystal suitable for X-ray crystallography was obtained by diffusing diethyl ether into solution of product in propylene carbonate (yield: 0.997 g, 96%). UV-Vis (MeCN): *λ*_max_ = 495 nm (*ε* = 241 mol^−1^ cm^−1^). IR (*ν*/cm^−1^): 2982 (w), 2936 (w), 1648 (s), 1461 (w), 1426 (w), 1405 (w), 1358 (m), 1292 (m), 1253 (s), 1214 (w), 1184 (m), 1154 (m), 1080 (w), 1005 (w), 9147 (s), 892 (m), 794 (m), 763 (w), 721 (w), 627 (s), 588 (m), 562 (w), 541 (w). *δ*_H_ (400 MHz, CD_3_CN) 4.95 (2H, d, *J* = 16.1 Hz), 4.68 (2H, d, *J* = 16.1 Hz), 4.59 (2H, d, *J* = 16.1 Hz), 4.42 (2H, d, *J* = 16.1 Hz), 3.16 (8H, q, *J* = 7.2 Hz), 1.21 (12H, t, *J* = 7.3 Hz). *δ*_C_ (101 MHz, CD_3_CN) 170.78, 170.33, 65.48, 64.72, 52.62, 7.26. HR-MS (ESI, positive ion mode) *m*/*z* calculated for C_8_H_20_N^+^ [M]^+^: 130.1590, found: 130.1596. MS (ESI, negative ion mode) *m*/*z* calculated for VBH^−^ [M]^−^: 342.9624, found: 343.0897.

##### Trimethylpropylammonium vanadium(v)-bis-hydroxyiminodiacetate [N_1113_]VBH (7)

Compound 3 (2.19 g, 0.00401 mol) was dissolved in 3 : 1 MeCN/EtOH to make 20 mL solution. FcPF_6_ (1.33 g, 0.00401 mol) was added to the solution and stirred for 20 min. 20 mL diethyl ether was added to aid precipitation of the complex and the precipitate was filtered which was then washed with 10 mL portion of diethyl ether five times until trace of ferrocene was removed. Finally, red crystalline powder was dried *in vacuo* (yield 1.53 g, 87%). UV-Vis (MeCN): *λ*_max_ = 495 nm (*ε* = 241.9 mol^−1^ cm^−1^). IR (*ν*/cm^−1^): 2951 (w), 1650 (s), 1478 (w), 1402 (w), 1351 (m), 1297 (m), 1245 (m), 1213 (w), 1154 (m), 967 (w), 915 (m), 895 (m), 763 (w), 721 (w), 624 (s), 589 (m), 564 (w), 542 (w). *δ*_H_ (400 MHz, CD_3_CN) 4.94 (2H, d, *J* = 16.1 Hz), 4.68 (2H, d, *J* = 16.1 Hz), 4.59 (2H, d, *J* = 16.0 Hz), 4.42 (2H, d, *J* = 16.0 Hz), 3.16 (2H, t, *J* = 8.4 Hz), 3.00 (9H, s), 1.74 (2H, h, *J* = 7.4 Hz), 0.95 (3H, t, *J* = 7.4 Hz). *δ*_C_ (101 MHz, DMSO) 170.33, 170.04, 66.77, 64.80, 64.16, 52.17, 15.68, 10.48. HR-MS (ESI, positive ion mode) *m*/*z* calculated for C_6_H_16_N^+^ [M]^+^: 102.1277, found: 102.1289. MS (ESI, negative ion mode) *m*/*z* calculated for VBH^−^ [M]^−^: 342.9624, found: 343.0724.

##### Trimethylbutylammonium vanadium(v)-bis-hydroxyiminodiacetate [N_1114_]VBH (8)

Compound 4 (1.43 g, 0.00249 mol) was dissolved in 10 mL ethanol followed by addition of FcPF_6_ (0.823 g, 0.00249 mol). The mixture was stirred for 20 min before adding 10 mL of diethyl ether. Upon addition of ether, red solid precipitated out which was then collected by filtration and washed with diethyl ether to remove trace of ferrocene. The product was dried *in vacuo* (yield 0.900 g, 79%). UV-Vis (MeCN): *λ*_max_ = 495 nm (*ε* = 242.7 mol^−1^ cm^−1^) IR (*ν*/cm^−1^): 2935 (w), 1650 (s), 1482 (w), 1400 (w), 1353 (m), 1298 (m), 1244 (m), 1215 (w), 1155 (m), 915 (m), 624 (s), 589 (m), 564 (w), 541 (w). *δ*_H_ (400 MHz, DMSO) 5.13 (2H, d, *J* = 16.4 Hz), 4.81 (2H, d, *J* = 16.3 Hz), 4.65 (2H, d, *J* = 16.5 Hz), 4.45 (2H, d, *J* = 16.4 Hz), 3.30–3.22 (2H, m), 3.03 (9H, s), 1.65 (2H, p, *J* = 7.4 Hz), 1.30 (2H, h, *J* = 7.4 Hz), 0.93 (3H, t, *J* = 7.4 Hz). *δ*_C_ (101 MHz, DMSO) 170.13, 169.84, 64.92, 64.64, 63.97, 51.98, 51.94, 51.90, 23.81, 18.95, 13.27. HR-MS (ESI, positive ion mode) *m*/*z* calculated for C_7_H_18_N^+^ [M]^+^: 116.1434, found: 116.1429. MS (ESI, negative ion mode) *m*/*z* calculated for VBH^−^ [M]^−^: 342.9624, found: 343.0967.

#### Viscosity measurement

The viscosity measurements were collected using the Cannon Instruments Semi-Micro Viscometer. Concentrations of 0.01 M– 0.55 M were used when collecting the viscosity data at ambient temperature (25 °C). The U-tube style viscometer allowed for the collection of kinematic viscosity in centiStokes (cSt) which was then converted to dynamic viscosity in centiPoise (cP) using the electrolyte density.

## Data availability

Data associated with this research can be found in the ESI† of this article.

## Author contributions

Conceptualization, funding acquisition: MLM (computational chemistry), PJC (synthetic chemistry), EA (flow battery performance). Computation and analysis: BRBV, MLM. Synthesis: SKP; characterization: SKP, JAG, PJC; experimental solubility: SKP. Viscosity measurements and analysis: TCG, EA. All authors contributed to writing the manuscript.

## Conflicts of interest

There are no conflicts to declare.

## Supplementary Material

SC-012-D1SC04990A-s001

SC-012-D1SC04990A-s002
