# Supplementary material for: Computational and experimental investigation of the effect of cation structure on the solubility of anionic flow battery active-materials
Source: Chem Sci. 2021 Nov 26;12(48):15892–907. doi: 10.1039/d1sc04990a (PMC8672735; doi:10.1039/d1sc04990a)
Supplement: SC-012-D1SC04990A-s001 [file SC-012-D1SC04990A-s001.pdf]

Electronic Supplementary Material (ESI) for Chemical Science.
This journal is © The Royal Society of Chemistry 2021

Electronic Supplementary Information

Computational and Experimental Investigation of the Effect of Cation Structure on the Solubility of Anionic Flow Battery Active-Materials

Benjoe Rey B. Visayas,[a] Shyam K. Pahari,[a] Tugba Ceren Gokoglan,[b] James A. Golen,[a] Ertan Agar,[b] Patrick J. Cappillino*[a] and Maricris L. Mayes*[a]

[a] *Department of Chemistry and Biochemistry, University of Massachusetts Dartmouth, MA 02747-2300, USA*
[b] *Department of Mechanical Engineering, Energy Engineering Graduate Program, University of Massachusetts Lowell, Lowell, MA 01854, USA*

email: maricris.mayes@umassd.edu
pcappillino@umassd.edu

Computational Details

The intrinsic solubility (S_o) of a substance is directly related to the difference in its stabilities in both the solid and solvated states. This relationship is demonstrated from the well-known Born-Fajans-Haber thermochemical correlation[1] (Scheme 1), which translates to

$$\Delta G^*_{dis} = \Delta G^*_{sub} + \Delta G^*_{sol} = -RTln(S_o V_m) \qquad \textbf{(1)}$$

where ΔG^*_{dis} is the free energy of dissolution and is the sum of the free energies of sublimation (ΔG^*_{sub}) and solvation (ΔG^*_{sol}) while R, T, and V_m are the gas constant, temperature, and the solid's molar volume, respectively. In this cycle, a substance first sublimes into the gas phase, then gets solvated into the solution phase and the free energies associated with each process determine its dissolution. The free energy of sublimation is roughly the negative of the lattice free energy (ΔG^o_{latt}).

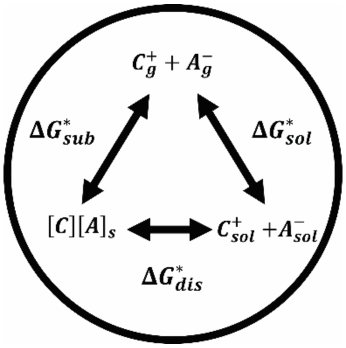

Scheme 1. The Born-Fajans-Haber thermochemical correlation

Sublimation free energy ΔG^*_{sub}

The free energy of sublimation (ΔG^*_{sub}) was obtained from the negative of the lattice free energy (ΔG^o_{latt}) and the isothermal expansion of an ideal gas:[2–4]

$$\Delta G^*_{sub} = -\Delta G^o_{latt} - RTln\left(\frac{V_m p_o}{RT}\right) \qquad \textbf{(2)}$$

where p_o is the standard condition pressure. The subsequent equations detail the calculation of the ΔG^o_{latt}.

$$\Delta G^o_{latt} = \Delta H^o_{latt} - T\Delta S^o_{latt} \qquad \textbf{(3)}$$

$$\Delta H^o_{latt} = \Delta U_{latt} + p\Delta V = \Delta U_{latt} - (n+m)RT \qquad \textbf{(4)}$$

where ΔH^o_{latt}, ΔS^o_{latt}, ΔU_{latt}, and ΔV are the lattice enthalpy, entropy, internal energy, and volume change, respectively. The $p\Delta V$ term in the enthalpy is generally negligible in solids at ordinary pressures, while $p\Delta V = RT$ for each gaseous species, hence the $(n+m)RT$ term in equation (4).[2–6] The lattice internal energy can be expressed as the sum of the lattice potential energy, ΔU_o, and the internal energy change, ΔU_{therm}, as shown in equation (5).

$$U_{latt} = \Delta U_o + \Delta U_{therm} \qquad \textbf{(5)}$$

ΔU_o can be expressed as

$$\Delta U_o = \frac{E_{total}^{[C]_n[A]_m} + E_{ZPE}^{[C]_n[A]_m}}{Z} - n\left(E_{total}^{C_{gas}^+} + E_{ZPE}^{C_{gas}^+}\right) - m\left(E_{total}^{A_{gas}^-} + E_{ZPE}^{A_{gas}^-}\right) \quad \textbf{(6)}$$

where E_{total}^{X} and E_{ZPE}^{X} ($X = C_{gas}^+$, A_{gas}^-, and $[C]_n[A]_m$) are the total energies and zero-point vibrational energies of the cations and anions in the gas phase and the solids, respectively. Z is the number of formula units included in the unit cell while n and m are the stoichiometric coefficients of the cation and anion, respectively, in one formula unit.[5] In a similar manner, the ΔU_{therm} and ΔS_{latt}^o were estimated from the difference between the sum of the corresponding quantities of the gas-phase ions and the solid phase using the rigid-rotor harmonic oscillator approximation.[5,7]

$$\Delta U_{therm} = \left(\frac{U_{vib}^{[C]_n[A]_m}}{Z} + (n+m)(3RT)\right) - n\left(U_{vib}^{C_{gas}^+} + U_{rot}^{C_{gas}^+} + U_{trans}^{C_{gas}^+}\right) - m\left(U_{vib}^{A_{gas}^-} + U_{rot}^{A_{gas}^-} + U_{trans}^{A_{gas}^-}\right) \quad \textbf{(7)}$$

$$\Delta S_{latt}^o = \frac{S_{vib}^{[C]_n[A]_m}}{Z} - n\left(S_{vib}^{C_{gas}^+} + S_{rot}^{C_{gas}^+} + S_{trans}^{C_{gas}^+}\right) - m\left(S_{vib}^{A_{gas}^-} + S_{rot}^{A_{gas}^-} + S_{trans}^{A_{gas}^-}\right) \quad \textbf{(8)}$$

Only the lattice vibrational contribution was considered for the internal energy and entropy whereas all three vibrational, rotational, and translational contributions were considered for the gas-phase ions. Additionally, three acoustic modes of the solids were approximated by $3RT$ per ion, hence the $(n+m)(3RT)$ term in equation (7).[8]

Solvation free energy ΔG_{sol}^*.

The standard free energy of solvation must be corrected for the free energy change ($\Delta G^{o\rightarrow *}$) associated with the solvation of 1 mole of each gaseous ion at 1 atmosphere (24.46 L/mol) into a 1 M solution. Thus,

$$\Delta G_{sol}^* = \Delta G_{sol}^o + (n+m)\Delta G^{o\rightarrow *} \quad \textbf{(9)}$$

where $\Delta G^{o\rightarrow *}$ is equal to 7.95 kJ/mol at 298 K.[9,10] The standard free energy of solvation (G_{sol}^o) was obtained using

$$\Delta G_{sol}^o = n\left(E_{total,SMD}^{C_{sol}^+} - E_{total}^{C_{gas}^+}\right) + m\left(E_{total,SMD}^{A_{sol}^-} - E_{total}^{A_{gas}^-}\right) \quad \textbf{(10)}$$

where $E_{total,SMD}^{X_{sol}}$ and $E_{total}^{X_{gas}}$ are the final energies of species X in solution and in gas phase, respectively.

Under the solvation model based on density (SMD)[11] method, the solvation energy of each ion can be decomposed into bulk-electrostatic (ΔG_{ENP}) and non-electrostatic (ΔG_{CDS}) contributions as shown in (11).[11,12]

$$\Delta G_{sol}^{o} = \Delta G_{ENP} + \Delta G_{CDS} \tag{11}$$

Table S1. Number of formula (*Z*), asymmetric (*Z*`) units, and trapped solvent impurities in the crystallized $[N_{xxxx}]_y[VBH]$ active materials in the +4 (where y=2) and +5 (where y=1) states and the corresponding molar volume, V_m(exp), in L·mol^{-1}. Also listed are the calculated molar volumes, V_m(calc), of the simplified crystals with the corresponding percent difference from V_m(exp).

	Vanadium (+4) species						Vanadium (+5) species					
xxxx	*Z*	*Z*`	Solvent	V_m (exp)	V_m (calc)	% Diff	*Z*	*Z*`	Solvent	V_m (exp)	V_m (calc)	% Diff
1111	2	1	4 H_2O, 2 MeOH	0.397	0.345	13.10	16	0.5	32 H_2O	0.302	0.259	14.24
2222	4	1	2 H_2O	0.435	0.427	1.84	2	1	---	0.322	0.315	2.17
3333	4	2	4 H_2O	0.605	0.573	5.29	2	1	---	0.378	0.380	0.53
4444	4	1	4 H_2O	0.723	0.726	0.41	4	1	4 MeCN	0.500	0.428	14.40

Table S2. SMD solvents used in ΔG_{sol}^{*} calculations.

Solvent	Dielectric Constant	Viscosity (25 °C), mPa·s
Dimethyl sulfoxide (DMSO)	46.826	2.00
N,N-dimethylacetamide	37.781	0.95
N,N-Dimethylformamide (DMF)	37.219	0.80
Nitromethane	36.562	0.63
Acetonitrile (MeCN)	35.688	0.34
Methanol	32.613	0.54
Propanonitrile	29.324	0.39 *
Nitroethane	28.29	0.66
Ethanol	24.852	1.08
1-Propanol	20.524	1.95
Acetone	20.493	0.30
2-Propanol	19.264	2.07
4-Methyl-2-pentanone	12.887	0.58 **
Dichloromethane	8.93	0.41
Tetrahydrofuran (THF)	7.4257	0.46
Ethyl acetate (Ethyl ethanoate)	5.9867	0.43
Toluene	2.3741	0.55
n-Hexane	1.8819	0.29

* at 30°C, ** at 20°C

Table S3. Calculated lattice thermodynamic properties, molar volumes, and free energies of sublimation of the $[N_{xxxx}]_y[VBH]$ active materials in the +4 (where y=2) and +5 (where y=1) states. Energy values are in units of kJ·mol^{-1} and molar volumes are in units of L·mol^{-1}.

	Vanadium (+4) species					Vanadium (+5) species				
xxxx	ΔH^o_{latt}	$T\Delta S^o_{latt}$	ΔG^o_{latt}	V_m	ΔG^*_{sub}	ΔH^o_{latt}	$T\Delta S^o_{latt}$	ΔG^o_{latt}	V_m	ΔG^*_{sub}
Symmetric										
1111	-1253.0	-196.3	-1056.6	0.345	1067.2	-522.6	-141.6	-381.0	0.259	392.3
2222	-1176.8	-218.5	-958.3	0.427	968.4	-514.2	-147.9	-366.2	0.315	377.0
3333	-1164.8	-240.1	-924.7	0.573	934.0	-518.3	-161.1	-357.2	0.380	367.5
4444	-1105.1	-244.4	-860.7	0.726	869.4	-505.8	-168.9	-336.9	0.428	346.9
Monosubstituted										
1112	-1216.4	-197.6	-1018.9	0.397	1029.1	-515.7	-143.3	-372.4	0.284	383.5
1113	-1223.3	-205.3	-1018.1	0.405	1028.2	-524.4	-151.1	-373.3	0.285	384.3
1114	-1246.7	-216.3	-1030.4	0.415	1040.5	-516.0	-156.3	-359.7	0.298	370.6
Symmetric disubstituted										
1122	-1211.1	-207.7	-1003.4	0.406	1013.6	-515.0	-146.9	-368.1	0.294	379.1
1133	-1178.0	-216.2	-961.8	0.470	971.6	-506.5	-149.7	-356.8	0.337	367.4
1144	-1180.2	-219.8	-960.4	0.571	969.7	-504.6	-156.0	-348.7	0.353	359.2
Asymmetric disubstituted										
1123	-1191.0	-208.9	-982.1	0.431	992.1	-503.2	-144.1	-359.1	0.316	369.9
1124	-1166.4	-218.7	-947.8	0.517	957.3	-509.9	-149.9	-360.0	0.339	370.6
1134	-1184.5	-216.7	-967.9	0.518	977.4	-515.7	-155.3	-360.3	0.345	370.9
Monosubstituted										
444c	-1099.3	-235.7	-863.6	0.719	872.3	-498.8	-160.1	-338.7	0.417	348.8
4441	-1138.1	-237.3	-900.8	0.584	910.0	-493.9	-161.8	-332.1	0.406	342.3
444s	-1130.4	-235.1	-895.3	0.700	904.1	-492.0	-160.8	-331.3	0.435	341.2
444t	-1148.4	-240.6	-907.9	0.654	916.9	-463.0	-162.1	-300.9	0.449	310.8
Symmetric disubstituted										
44cc	-1152.2	-229.8	-922.3	0.560	931.7	-483.6	-147.6	-336.0	0.413	346.2
44ss	-1155.0	-243.4	-911.6	0.639	920.6	-480.6	-164.2	-316.3	0.434	326.3
44tt	-1130.9	-231.6	-899.3	0.676	908.2	-484.7	-154.8	-329.9	0.440	339.8
Asymmetric disubstituted										
441c	-1158.0	-223.3	-934.7	0.543	944.2	-512.0	-155.0	-357.1	0.368	367.5
44cs	-1135.1	-224.5	-910.6	0.647	919.6	-495.1	-152.5	-342.6	0.410	352.7
44ct	-1119.5	-226.2	-893.3	0.644	902.3	-483.4	-156.9	-326.6	0.425	336.6
441s	-1169.2	-230.9	-938.3	0.575	947.6	-504.2	-156.3	-347.9	0.382	358.2
441t	-1104.8	-229.2	-875.7	0.618	884.8	-494.8	-160.3	-334.5	0.385	344.8
44st	-1135.9	-230.1	-905.8	0.666	914.7	-486.1	-162.1	-324.0	0.422	334.0

Table S4. Heatmap of the free energies of solvation (ΔG^{*}_{sol}), in kJ/mol units, of the $[N_{xxxx}]_2$[VBH] active materials. Red-yellow-green cell format indicates the favorability of solvation from least to most favorable ΔG^{*}_{sol}.

xxxx	Dimethyl sulfoxide	N,N-Dimethylacetate	N,N-Dimethylformamide	Nitromethane	Aceonitrile	Methanol	Propanonitrile	Nitroethane	Ethanol	1-Propanol	Acetone	2-Propanol	4-Methyl-2-pentanone	Dichloromethane	Tetrahydrofuran	Ethyl acetate	Toluene	n-Hexane
1111	-1099	-1106	-1103	-1098	-1107	-1156	-1099	-1091	-1143	-1131	-1089	-1127	-1055	-1022	-984	-951	-667	-541
2222	-1047	-1057	-1054	-1049	-1060	-1113	-1052	-1043	-1100	-1089	-1044	-1085	-1011	-983	-943	-912	-645	-525
3333	-1025	-1040	-1036	-1031	-1045	-1098	-1038	-1028	-1087	-1075	-1032	-1073	-1000	-975	-931	-902	-645	-528
4444	-1024	-1045	-1040	-1034	-1050	-1104	-1045	-1033	-1093	-1082	-1039	-1080	-1008	-985	-938	-910	-658	-541
1112	-1085	-1093	-1090	-1085	-1094	-1144	-1086	-1078	-1132	-1120	-1077	-1116	-1043	-1011	-973	-940	-661	-536
1113	-1079	-1088	-1084	-1079	-1089	-1140	-1081	-1073	-1127	-1116	-1073	-1112	-1039	-1008	-969	-937	-660	-536
1114	-1077	-1087	-1084	-1078	-1089	-1140	-1082	-1073	-1127	-1116	-1073	-1112	-1039	-1009	-969	-937	-662	-539
444c	-1024	-1041	-1036	-1030	-1045	-1101	-1039	-1028	-1089	-1078	-1033	-1075	-1001	-975	-931	-902	-644	-526
4441	-1040	-1056	-1052	-1046	-1061	-1113	-1055	-1044	-1102	-1091	-1048	-1088	-1016	-991	-946	-917	-658	-539
444s	-1016	-1036	-1031	-1026	-1041	-1095	-1036	-1024	-1084	-1073	-1030	-1071	-999	-976	-930	-902	-650	-534
444t	-1014	-1033	-1028	-1023	-1038	-1092	-1033	-1021	-1082	-1070	-1027	-1068	-996	-973	-927	-899	-647	-531
1122	-1072	-1081	-1077	-1072	-1083	-1134	-1074	-1066	-1121	-1109	-1066	-1106	-1032	-1002	-963	-931	-656	-533
1133	-1060	-1072	-1068	-1063	-1074	-1126	-1067	-1058	-1113	-1102	-1059	-1098	-1025	-997	-956	-925	-655	-533
1144	-1058	-1071	-1067	-1062	-1074	-1126	-1067	-1058	-1114	-1103	-1060	-1100	-1027	-999	-957	-926	-659	-538
44cc	-1011	-1025	-1021	-1015	-1029	-1087	-1022	-1011	-1074	-1062	-1016	-1060	-983	-956	-914	-885	-624	-507
44ss	-1008	-1027	-1022	-1017	-1032	-1086	-1027	-1015	-1075	-1064	-1021	-1062	-989	-967	-921	-893	-642	-527
44tt	-1003	-1020	-1016	-1011	-1026	-1079	-1020	-1008	-1068	-1057	-1013	-1054	-982	-959	-915	-887	-636	-521
1123	-1066	-1076	-1073	-1067	-1078	-1130	-1071	-1062	-1117	-1106	-1062	-1102	-1029	-999	-959	-928	-655	-533
1124	-1064	-1076	-1072	-1067	-1078	-1130	-1071	-1062	-1117	-1106	-1063	-1102	-1029	-1000	-960	-928	-657	-535
1134	-1059	-1071	-1068	-1062	-1074	-1126	-1067	-1058	-1114	-1102	-1059	-1099	-1026	-998	-957	-926	-657	-536
441c	-1046	-1060	-1056	-1050	-1063	-1118	-1056	-1046	-1106	-1094	-1049	-1091	-1016	-988	-946	-915	-647	-527
44cs	-1014	-1030	-1026	-1020	-1035	-1090	-1029	-1017	-1079	-1067	-1022	-1064	-990	-965	-922	-893	-636	-519
44ct	-1013	-1029	-1024	-1019	-1033	-1089	-1027	-1016	-1077	-1065	-1020	-1063	-988	-963	-920	-891	-634	-518
441s	-1032	-1048	-1044	-1038	-1053	-1105	-1046	-1036	-1094	-1083	-1039	-1080	-1008	-982	-938	-909	-650	-533
441t	-1030	-1046	-1042	-1036	-1050	-1103	-1044	-1033	-1092	-1080	-1037	-1078	-1005	-980	-936	-907	-648	-530
44st	-1007	-1024	-1020	-1015	-1030	-1084	-1024	-1013	-1073	-1062	-1018	-1059	-987	-964	-919	-891	-640	-525

Table S5. Heatmap of the free energies of solvation (ΔG^{*}_{sol}), in kJ/mol units, of the [N_{xxxx}][VBH] active materials. Red-yellow-green cell format indicates the favorability of solvation from least to most favorable ΔG^{*}_{sol}.

xxxx	Dimethyl sulfoxide	N,N-Dimethylacetate	N,N-Dimethylformamide	Nitromethane	Aceonitrile	Methanol	Propanonitrile	Nitroethane	Ethanol	1-Propanol	Acetone	2-Propanol	4-Methyl-2-pentanone	Dichloromethane	Tetrahydrofuran	Ethyl acetate	Toluene	n-Hexane
1111	-461	-467	-465	-452	-458	-461	-459	-454	-460	-455	-457	-458	-446	-421	-415	-402	-283	-229
2222	-435	-443	-440	-427	-434	-439	-436	-430	-439	-433	-434	-436	-425	-402	-394	-382	-272	-222
3333	-424	-435	-432	-418	-427	-432	-429	-422	-432	-427	-428	-430	-419	-398	-388	-377	-272	-223
4444	-424	-437	-433	-420	-429	-435	-432	-425	-435	-430	-431	-434	-423	-403	-392	-381	-279	-230
1112	-454	-461	-458	-445	-452	-455	-453	-447	-454	-449	-451	-452	-440	-416	-409	-396	-280	-227
1113	-451	-458	-456	-442	-449	-453	-451	-445	-452	-447	-448	-450	-438	-415	-407	-395	-280	-227
1114	-450	-458	-455	-442	-449	-453	-451	-445	-452	-447	-449	-450	-439	-415	-407	-395	-281	-228
444c	-423	-435	-432	-418	-427	-433	-430	-422	-433	-428	-428	-431	-419	-398	-388	-377	-271	-222
4441	-431	-442	-439	-426	-435	-440	-437	-430	-440	-434	-436	-438	-427	-406	-396	-385	-278	-228
444s	-420	-432	-429	-416	-425	-431	-428	-421	-431	-426	-427	-429	-418	-399	-387	-377	-275	-226
444t	-419	-431	-428	-414	-424	-429	-426	-419	-429	-424	-425	-428	-417	-397	-386	-376	-273	-225
1122	-447	-455	-452	-439	-446	-450	-447	-442	-449	-444	-445	-447	-435	-411	-404	-392	-278	-225
1133	-442	-450	-447	-434	-441	-446	-443	-437	-445	-440	-441	-443	-432	-409	-401	-389	-277	-226
1144	-440	-450	-447	-434	-442	-446	-444	-437	-446	-440	-442	-444	-432	-410	-401	-389	-279	-228
44cc	-417	-427	-424	-411	-419	-426	-421	-414	-426	-420	-420	-424	-410	-389	-380	-369	-261	-213
44ss	-415	-428	-425	-411	-421	-426	-423	-416	-426	-421	-422	-425	-414	-394	-383	-373	-271	-223
44tt	-413	-424	-421	-408	-417	-423	-420	-413	-423	-418	-419	-421	-410	-390	-380	-370	-268	-220
1123	-444	-452	-450	-437	-444	-448	-445	-439	-447	-442	-443	-445	-433	-410	-402	-390	-277	-225
1124	-444	-452	-449	-436	-443	-448	-445	-439	-447	-442	-443	-445	-434	-411	-403	-390	-278	-227
1134	-441	-450	-447	-434	-441	-446	-444	-437	-445	-440	-442	-443	-432	-410	-401	-389	-278	-227
441c	-434	-444	-441	-428	-436	-442	-438	-431	-441	-436	-436	-439	-427	-405	-395	-384	-273	-223
44cs	-418	-429	-426	-413	-422	-428	-424	-417	-428	-423	-423	-426	-414	-393	-383	-373	-268	-219
44ct	-418	-429	-426	-412	-421	-427	-423	-416	-427	-422	-422	-425	-413	-392	-383	-372	-267	-218
441s	-427	-438	-435	-422	-431	-436	-433	-426	-436	-430	-432	-434	-423	-402	-392	-381	-275	-225
441t	-426	-437	-434	-421	-429	-435	-432	-425	-434	-429	-430	-433	-421	-400	-391	-380	-274	-224
44st	-415	-427	-424	-411	-419	-425	-422	-415	-425	-420	-421	-423	-412	-393	-382	-372	-270	-221

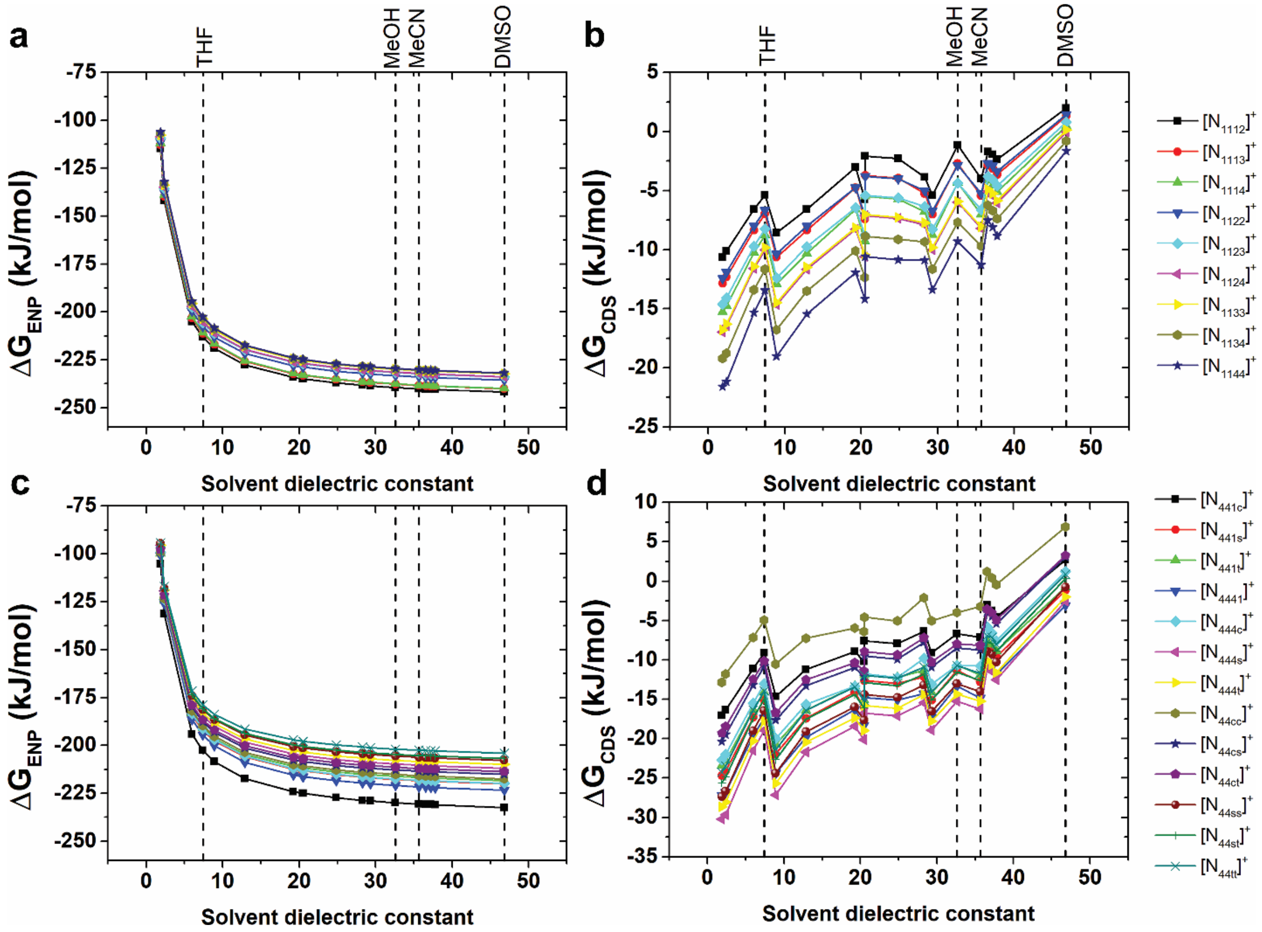

Fig. S1. The electrostatic (ΔG_{ENP}) and cavity-dispersion (ΔG_{CDS}) components of the SMD solvation energies of the asymmetric cations: **a** shows the ΔG_{ENP} while **b** shows the ΔG_{CDS} of the asymmetric $[N_{1111}]^+$-based cations across the different solvents used; panels **c** and **d** show the ΔG_{ENP} and ΔG_{CDS}, respectively, of the $[N_{4444}]^+$-based cations.

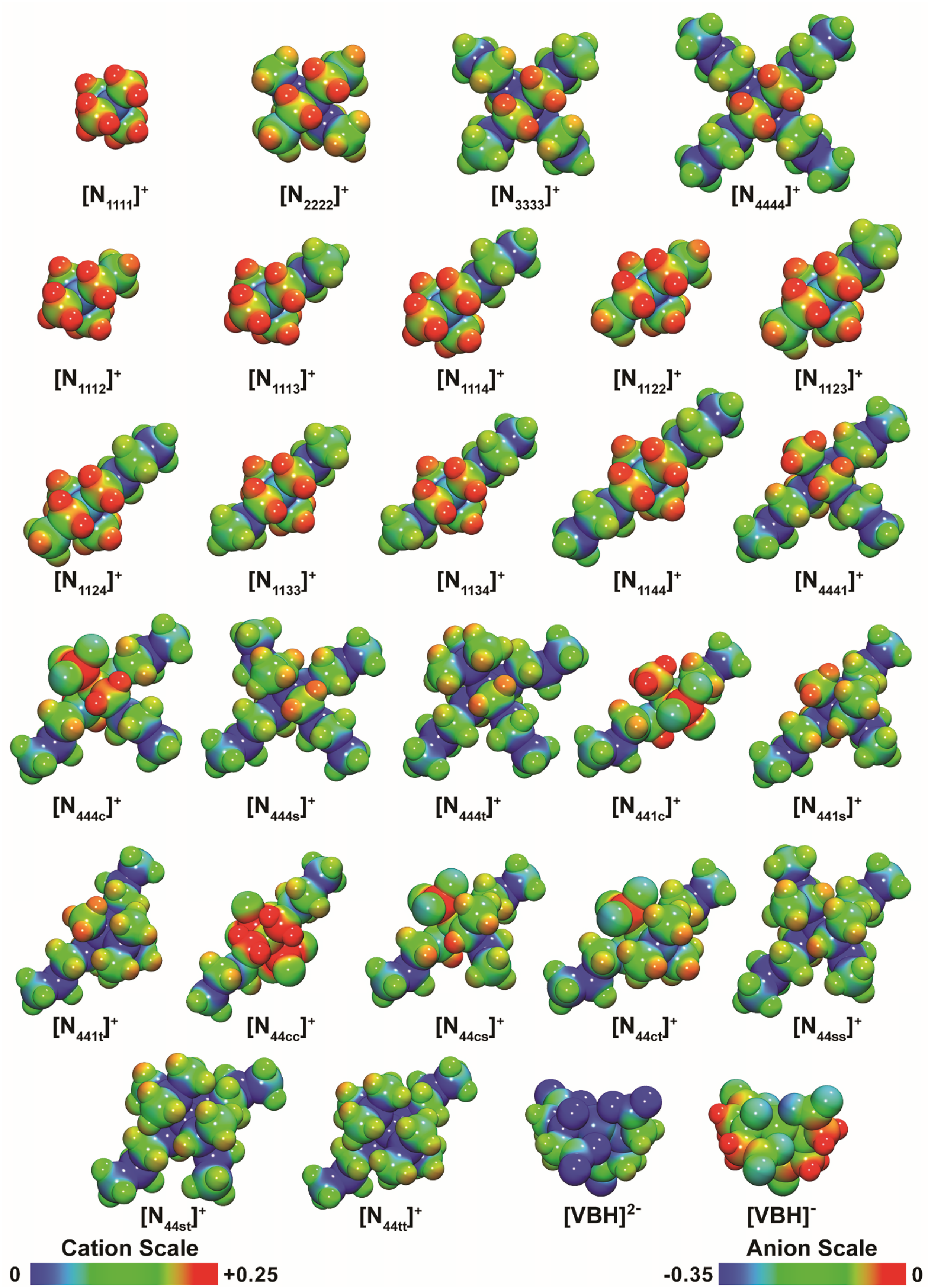

Fig. S2. Electrostatic potential maps from natural atomic charges of the free ions in the gas phase.

Table S6. Crystal data and structure refinement summary.

Compound	[N1111]VBH	[N2222]VBH	$[N1114]_2VBH$
Identification code	UMD702_a	UMD2266f_a	UMD1830B_a
Empirical formula	$C_{12}H_{25}N_3O_{12}V$	$C_{16}H_{28}N_3O_{10}V$	$C_{22}H_{48}N_4O_{12}V$
Formula weight	454.29	473.35	611.58
Temperature/K	200(2)	297(2)	200(2)
Crystal system	orthorhombic	monoclinic	monoclinic
Space group	Fddd	$P2_1$	$P2_1/c$
a/Å	15.926(2)	11.8775(7)	13.3481(10)
b/Å	18.696(3)	8.1637(5)	12.7135(10)
c/Å	26.925(4)	12.1364(6)	18.0999(13)
α/°	90.00	90	90
β/°	90.00	114.799(2)	96.632(2)
γ/°	90.00	90	90
Volume/$Å^3$	8017(2)	1068.28(11)	3051.0(4)
Z	16	2	4
$\rho_{calc}g/cm^3$	1.506	1.472	1.331
μ/mm^{-1}	0.559	0.521	0.387
F(000)	3792.0	496.0	1308.0
Crystal size/mm^3	0.3 × 0.14 × 0.08	0.22 × 0.15 × 0.1	0.32 × 0.28 × 0.27
Radiation	MoKα (λ = 0.71073)	MoKα (λ = 0.71073)	MoKα (λ = 0.71073)
2Θ range for data collection/°	5.94 to 52.82	6.298 to 52.79	6.086 to 50.884
Index ranges	-19 ≤ h ≤ 19, -23 ≤ k ≤ 23, -33 ≤ l ≤ 33	-14 ≤ h ≤ 14, -10 ≤ k ≤ 10, -15 ≤ l ≤ 14	-16 ≤ h ≤ 16, -15 ≤ k ≤ 15, -21 ≤ l ≤ 21
Reflections collected	56007	34061	82551
Independent reflections	2073 [R_{int} = 0.0479, R_{sigma} = 0.0143]	4351 [R_{int} = 0.0329, R_{sigma} = 0.0207]	5619 [R_{int} = 0.0474, R_{sigma} = 0.0171]
Data/restraints/parameters	2073/2/138	4351/1/275	5619/370/390
Goodness-of-fit on F^2	1.096	1.016	1.085
Final R indexes [I>=2σ (I)]	R_1 = 0.0297, wR_2 = 0.0715	R_1 = 0.0215, wR_2 = 0.0564	R_1 = 0.0358, wR_2 = 0.0802
Final R indexes [all data]	R_1 = 0.0392, wR_2 = 0.0759	R_1 = 0.0231, wR_2 = 0.0575	R_1 = 0.0443, wR_2 = 0.0857
Largest diff. peak/hole / e $Å^{-3}$	0.24/-0.31	0.16/-0.14	0.75/-0.50

NMR spectra

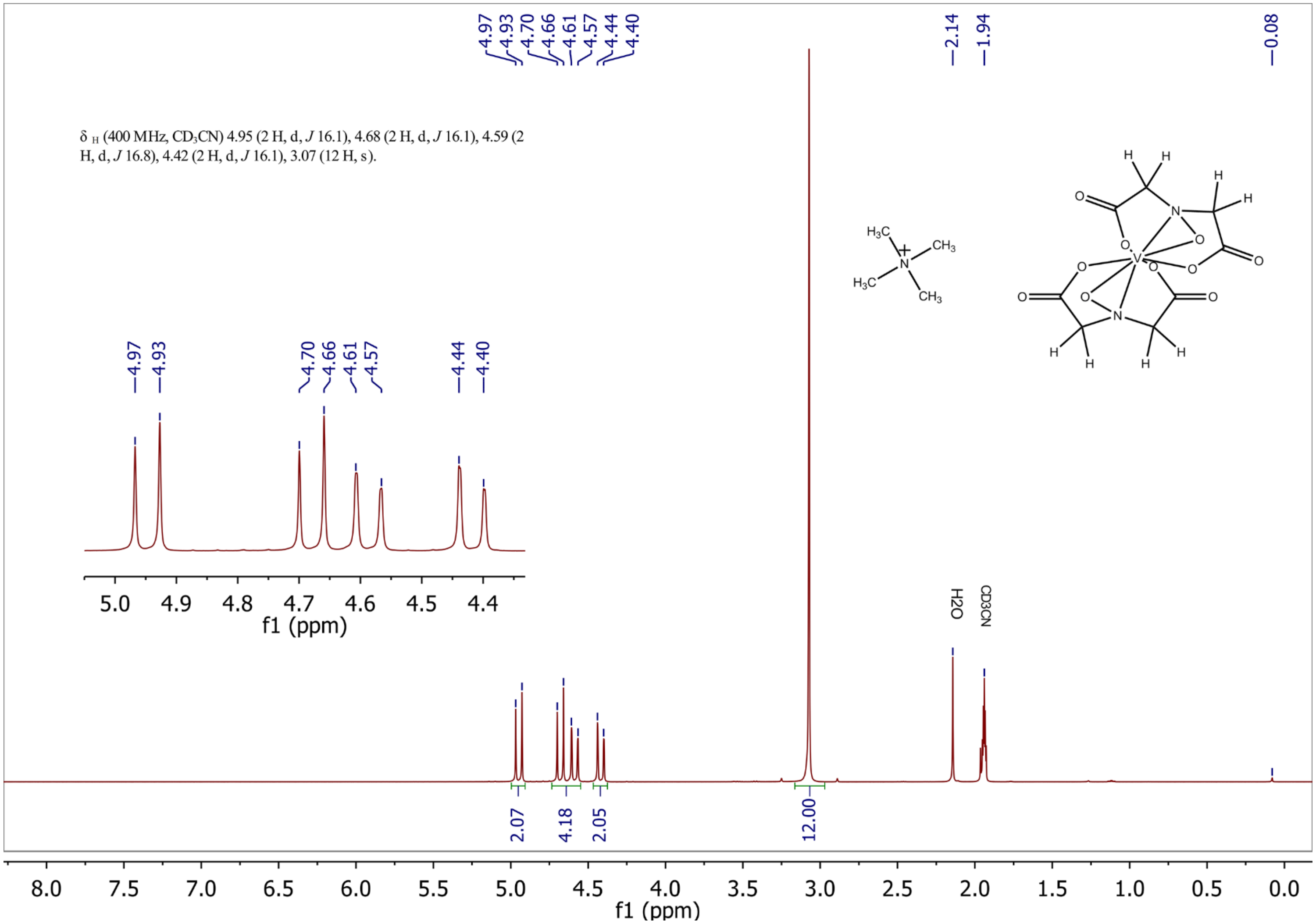

Fig. S3. ^{1}H-NMR of [N_{1111}]VBH

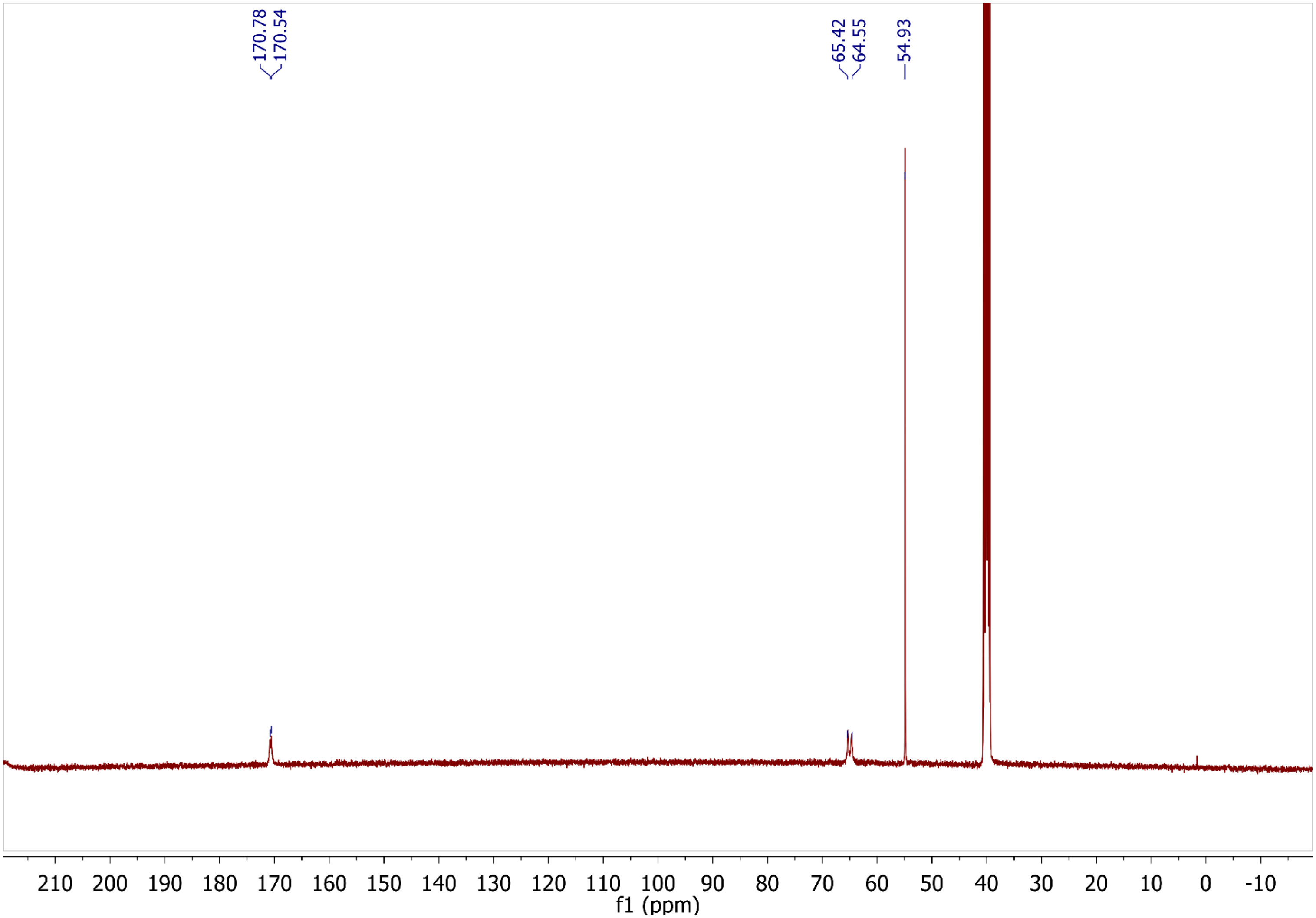

Fig. S4. ^{13}C-NMR of [N_{1111}]VBH

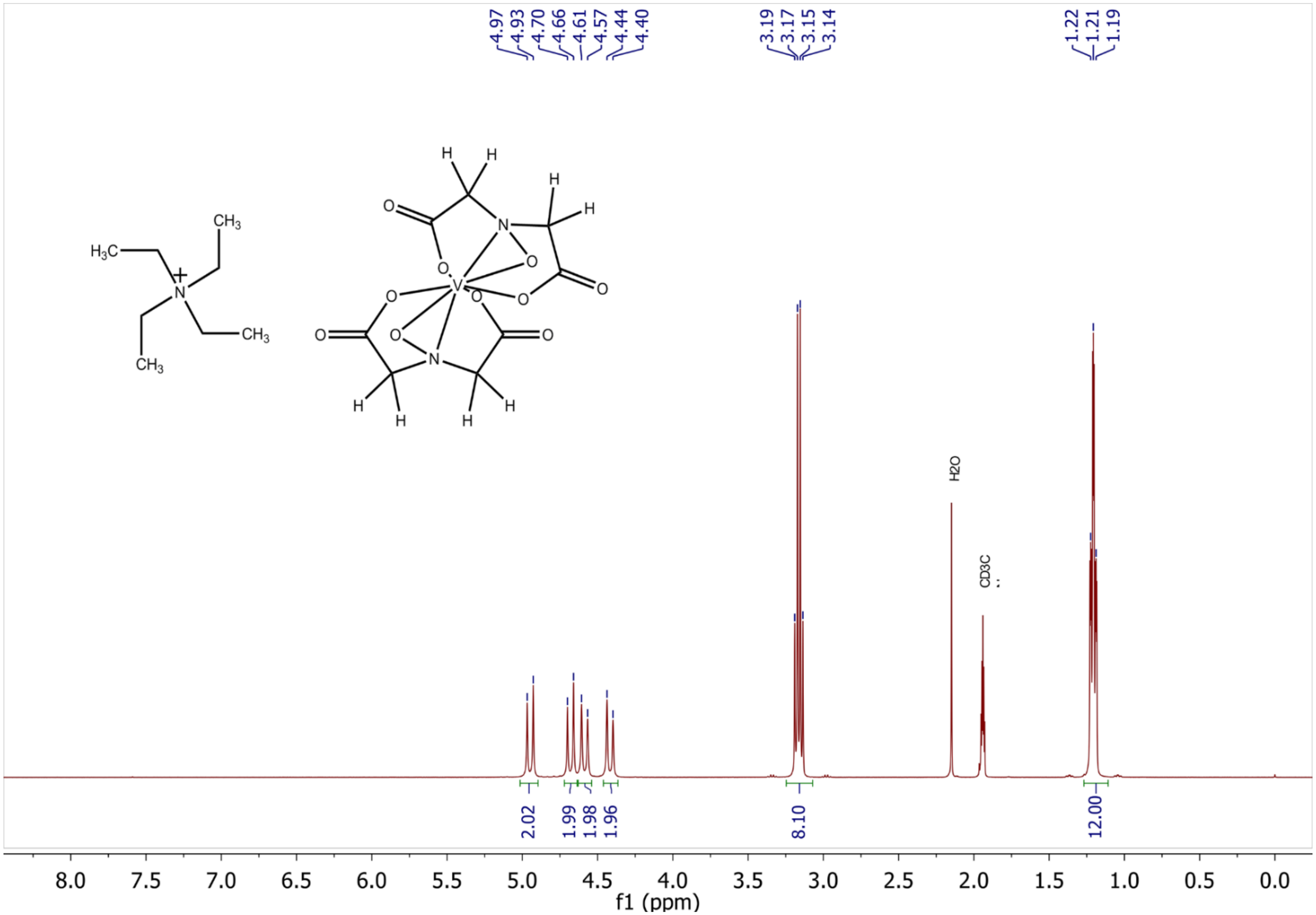

Fig. S5. ^{1}H-NMR of [N_{2222}]VBH

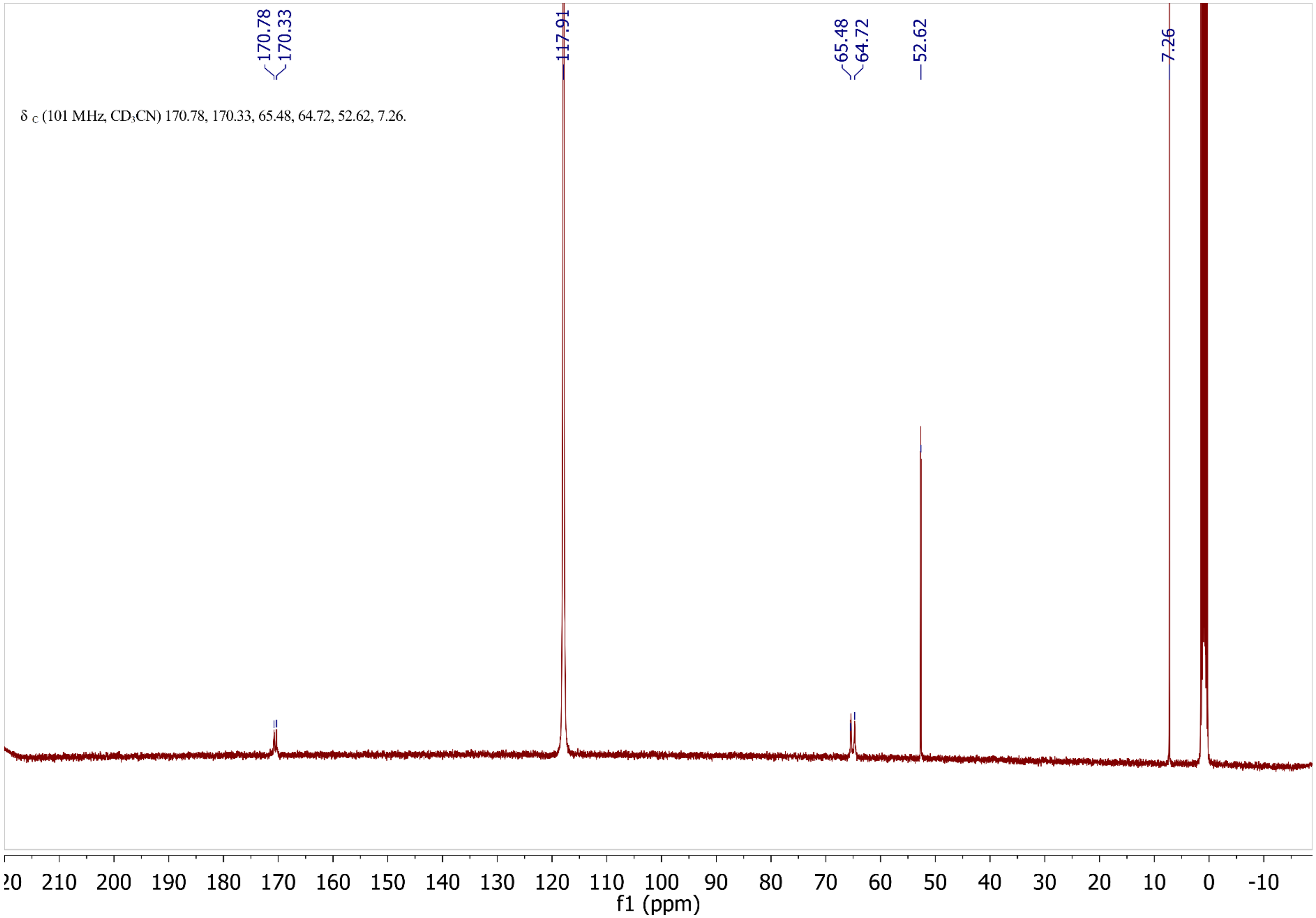

Fig. S6. ^{13}C-NMR of [N_{2222}]VBH

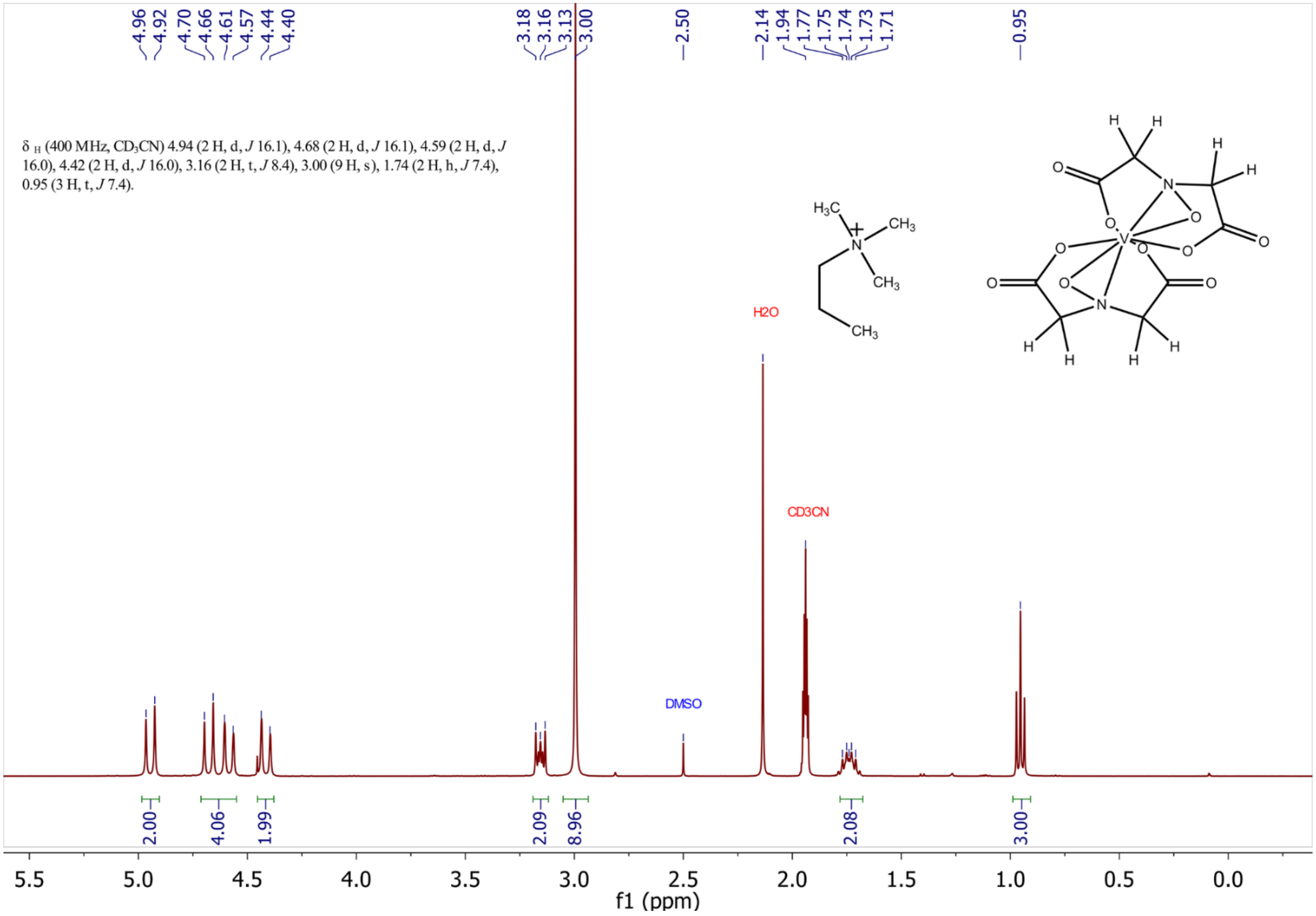

Fig. S7. ^{1}H-NMR of [N_{1113}]VBH

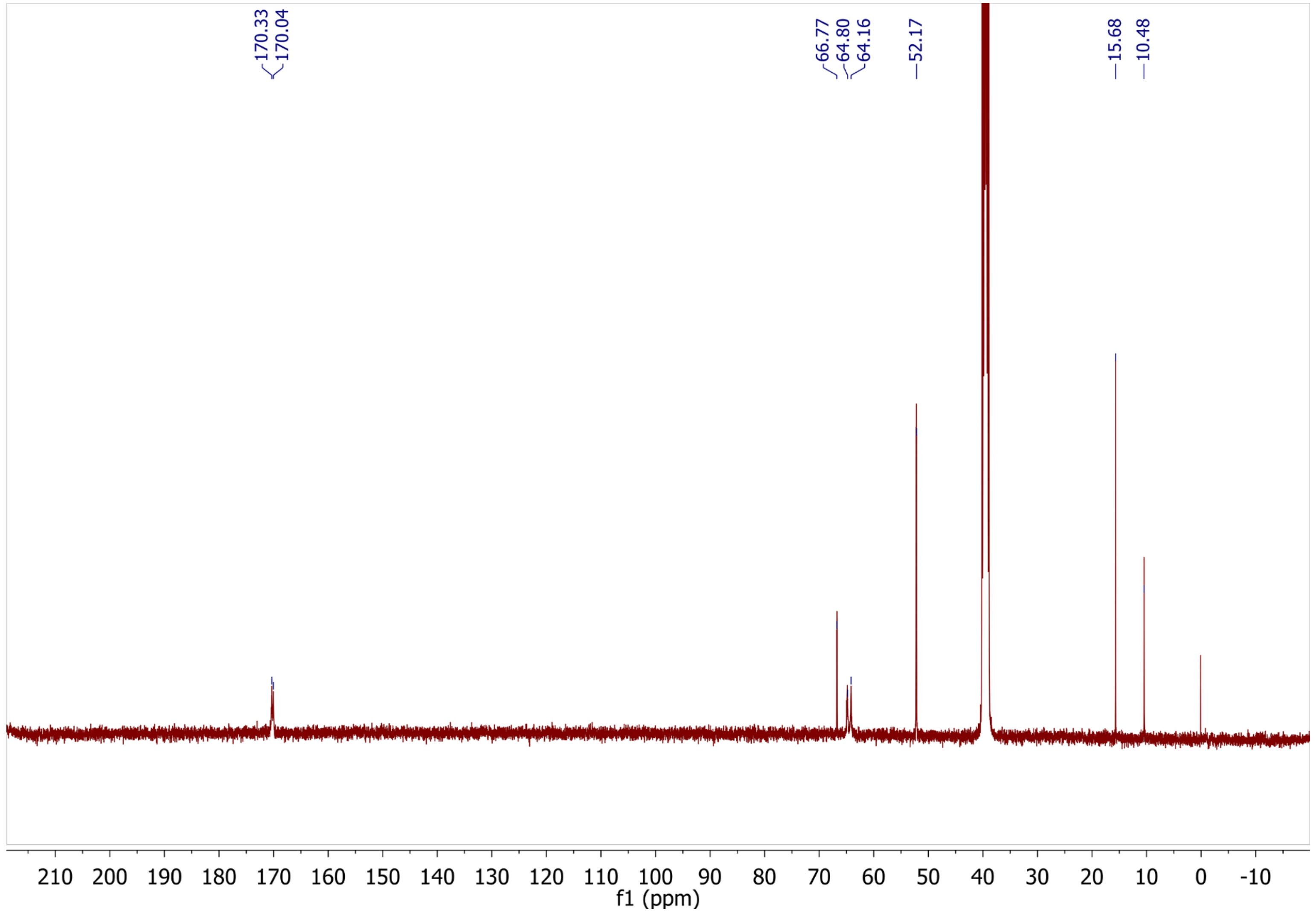

Fig. S8. ^{13}C-NMR of [N_{1113}]VBH

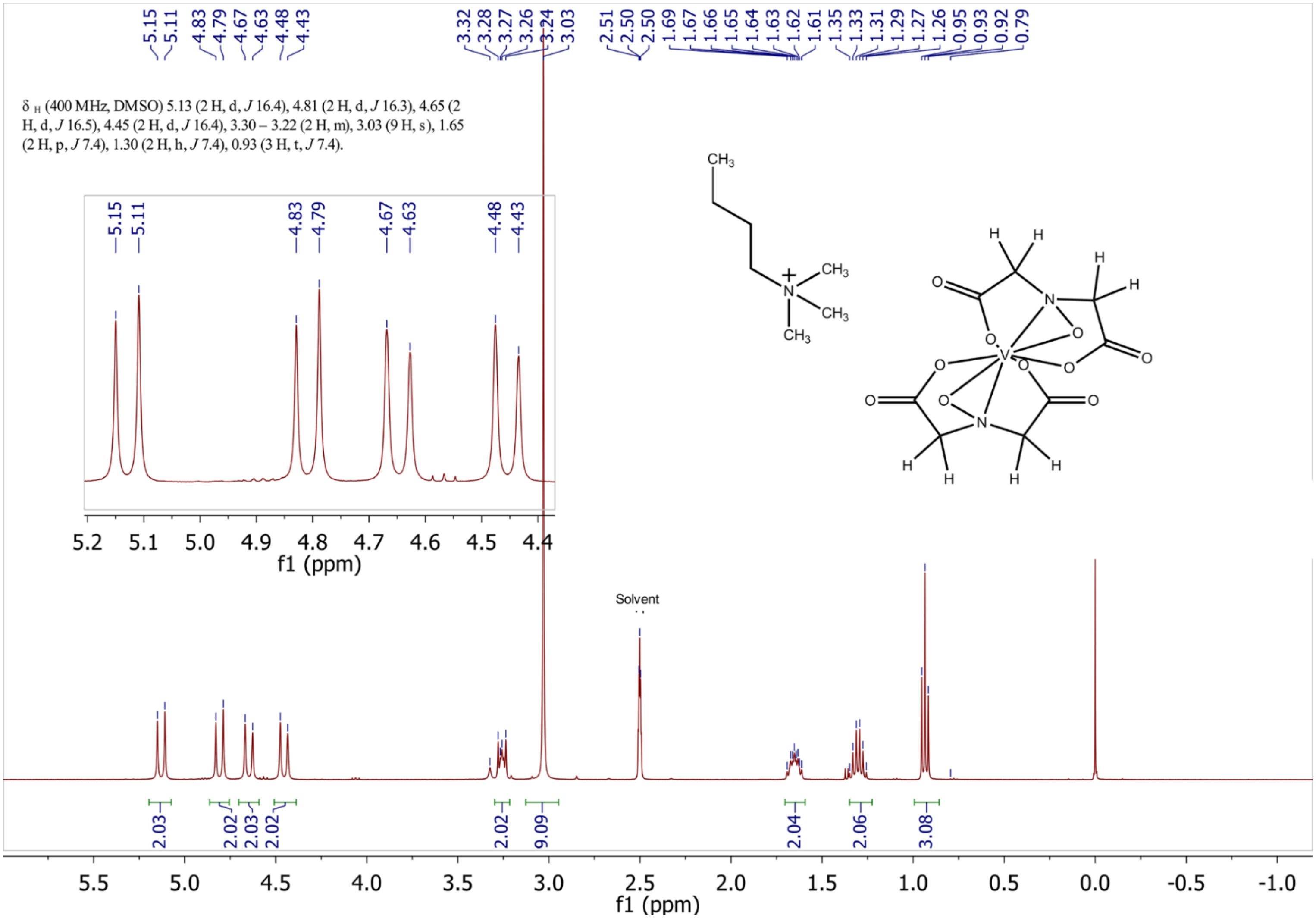

Fig. S9. ^{1}H-NMR of [N_{1114}]VBH

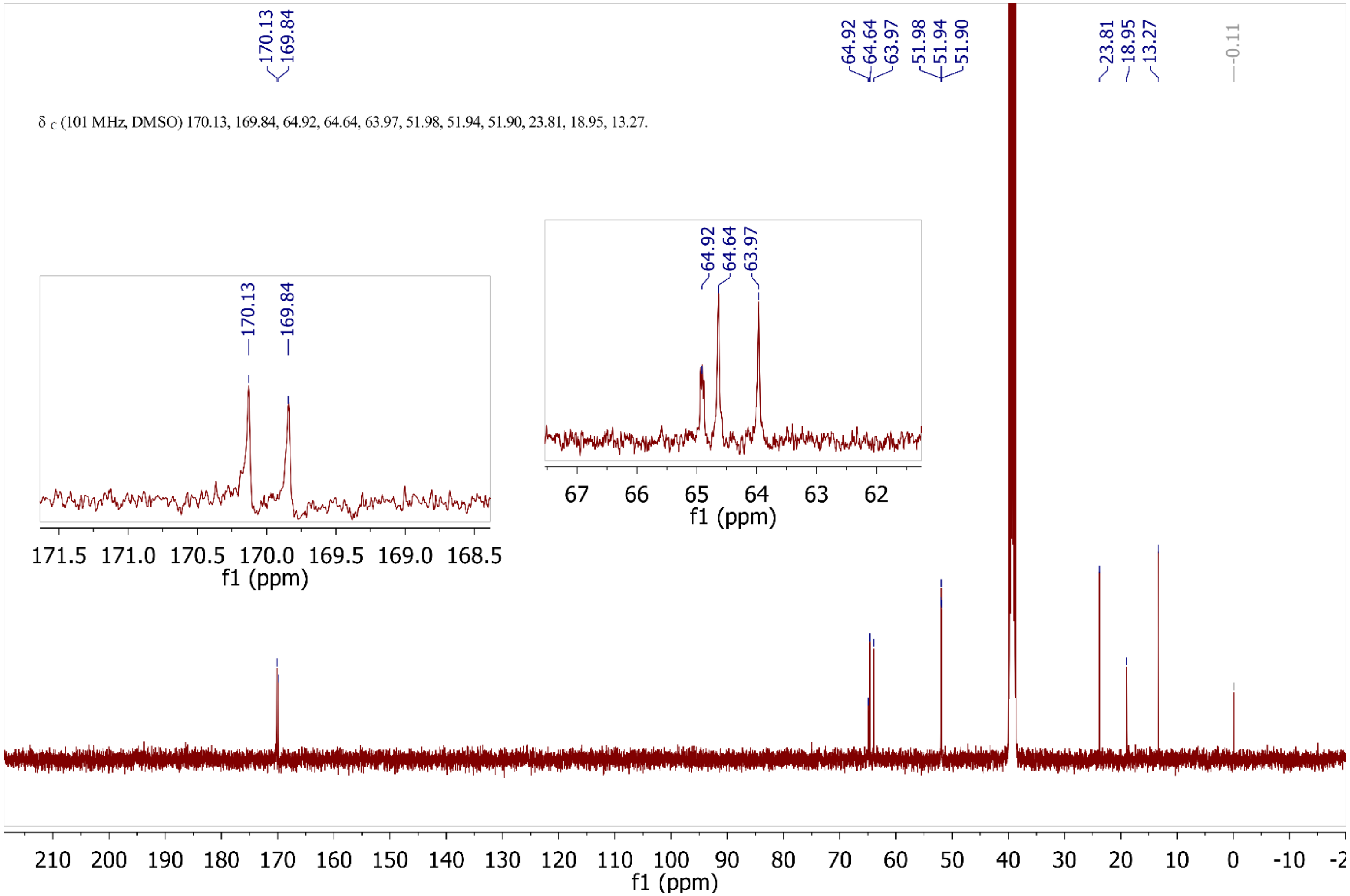

Fig. S10. ^{13}C-NMR of [N_{1114}]VBH

Table S7. Cartesian coordinates of the formula units in the DFT-optimized crystals.

**Reduced (V^{+4}) State**	**Oxidized (V^{+5}) State**
**Symmetric**	
**$[N_{1111}]_2[VBH]$**	**$[N_{1111}][VBH]$**
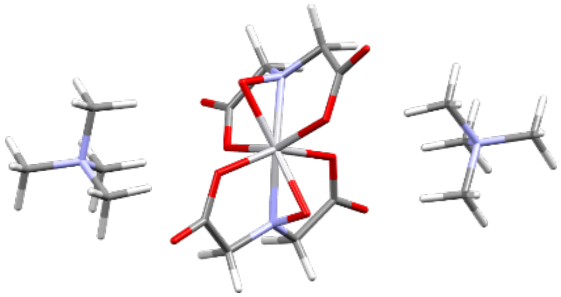	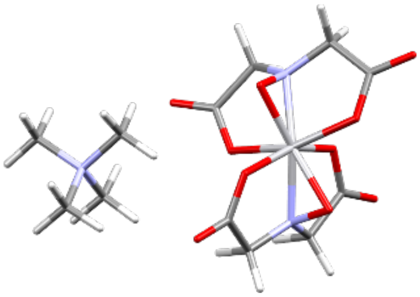
63	46
N -3.748725 -3.357626 2.163700	O -1.528721 1.269096 0.103523
C -2.412568 -3.628849 2.793092	O -3.220906 2.013053 1.386607
H -2.015621 -4.534117 2.321823	O 2.336292 -2.213732 2.466184
H -1.770597 -2.763102 2.596176	O 1.281241 -1.425737 0.641797
C -4.389490 -2.180383 2.832807	O 0.757738 1.008673 1.476085
H -5.362844 -2.020155 2.356636	N -0.212294 0.181070 1.996565
H -4.528982 -2.430695 3.888512	C -2.135170 1.446602 1.257599
C -3.548468 -3.045905 0.708829	C -1.416311 0.862829 2.463962
H -3.117303 -3.924972 0.220771	H -2.041397 0.125616 2.983798
H -2.878830 -2.180301 0.656895	H -1.140857 1.672257 3.153073
C -4.620280 -4.566178 2.340611	C 0.288306 -0.977475 2.743762
H -4.678703 -4.779597 3.412566	H 0.567115 -0.692578 3.765087
H -5.620122 -4.313560 1.976038	H -0.536431 -1.702360 2.787340
H -3.723152 -1.320187 2.704075	C 1.412601 -1.591643 1.936868
H -4.521206 -2.793852 0.273959	V 0.000000 0.000000 0.000000
H -2.556736 -3.765551 3.871842	O 1.627991 0.826452 -0.833773
H -4.176360 -5.386851 1.766427	O 3.220868 0.613727 -2.397490
V 0.000000 0.000000 0.000000	O -2.643423 -3.018049 -0.660417
O 1.941460 0.275732 -0.686253	O -1.426902 -1.393717 0.300288
O 4.107492 0.438703 -0.084252	O -0.620578 -0.016272 -1.858956
O 0.926525 -1.278482 1.220936	N 0.184973 -1.116701 -1.668437
O -1.960658 -0.156191 3.635925	C 2.187106 0.227402 -1.848464
O -1.505158 -0.281773 1.435983	C 1.496116 -1.053347 -2.308043
O -0.185011 2.030842 0.498158	H 2.075089 -1.920652 -1.961409
O -1.565272 3.805105 0.399149	H 1.412233 -1.086067 -3.402556
O -0.422332 0.756447 -1.798111	C -0.536750 -2.391688 -1.666782
O -1.889073 -2.734539 -2.454529	H -0.933526 -2.582494 -2.672185
O -0.497044 -1.770525 -0.966725	H 0.160588 -3.189836 -1.365393
N 1.060864 0.012147 1.718580	C -1.641558 -2.294791 -0.617610
N -1.604238 0.550122 -1.096730	C 4.776390 -2.070314 0.095400
C 2.895705 0.391745 0.186365	H 4.134260 -1.821233 -0.755912
C 2.432359 0.503962 1.640353	H 4.160856 -2.412278 0.936558
H 3.088858 -0.076807 2.301686	C 4.531679 0.227449 0.948266
H 2.445212 1.550349 1.977492	H 3.857163 0.464821 0.121559

C 0.284034 0.292674 2.925404
H 0.362626 1.370738 3.125680
H 0.690718 -0.241614 3.795364
C -1.175124 -0.078129 2.670140
C -1.280002 2.618903 0.139434
C -2.275722 1.768625 -0.646305
H -3.103753 1.466114 0.010457
H -2.703231 2.324883 -1.492179
C -2.412617 -0.524663 -1.662739
H -2.773368 -0.250536 -2.662917
H -3.286464 -0.679747 -1.013826
C -1.548648 -1.785875 -1.727895
N 2.121329 5.042326 -0.301367
C 1.208634 5.472305 0.806002
H 0.496934 4.659011 0.986355
H 0.704278 6.389051 0.486899
C 2.833349 3.786134 0.109687
H 3.370099 3.984660 1.043147
H 3.526378 3.502142 -0.687770
C 1.316377 4.764558 -1.538264
H 0.656839 3.916797 -1.322083
H 0.729209 5.658917 -1.780155
C 3.097654 6.142653 -0.597368
H 3.597457 6.406943 0.338996
H 3.808037 5.769200 -1.343116
H 2.028075 4.522259 -2.334486
H 2.526677 7.007665 -0.949196
H 1.830036 5.676136 1.684714
H 2.064947 3.022654 0.274128

$[N_{2222}]_2[VBH]$

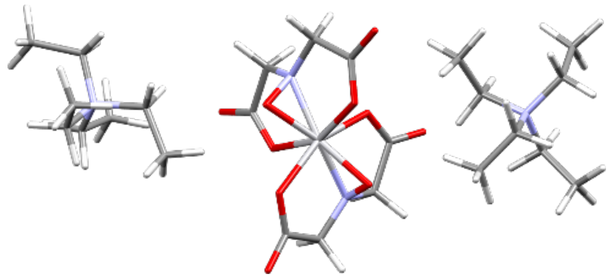

87

C 0.720982 6.437042 -3.981963
H -0.138045 7.024298 -4.329859
H 0.826965 5.572828 -4.647167
H 1.616264 7.065062 -4.064116
N 1.644483 5.581547 -1.742579
C 1.094621 4.813559 -0.556078
H 0.444296 4.039191 -0.965455
H 0.446362 5.516063 -0.021955
C 2.155321 4.197000 0.317555
H 1.663961 3.798873 1.212036

H 3.969017 -0.181393 1.794911
C 6.285994 -0.297036 -0.678224
H 6.792853 0.624249 -0.373188
H 7.009225 -1.064494 -0.969146
N 5.507034 -0.819275 0.495619
C 6.443651 -1.131867 1.624396
H 7.198263 -1.839708 1.260859
H 6.923248 -0.195512 1.926542
H 5.516271 -2.823087 -0.196579
H 5.109213 1.118382 1.220598
H 5.569720 -0.094080 -1.479923
H 5.849509 -1.543900 2.448861

$[N_{2222}][VBH]$

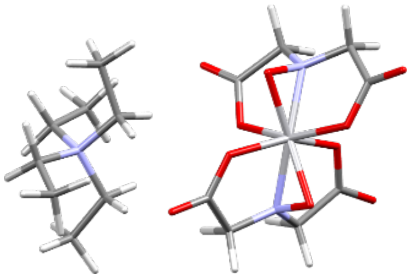

82

O -1.455835 1.286909 -0.402973
O -2.518055 3.192114 0.162914
O 2.441800 -0.938390 3.171949
O 1.339994 -0.977398 1.206365
O 0.947333 1.571508 0.626004
N 0.060530 1.226036 1.624322
C -1.740396 2.277050 0.418067
C -1.045768 2.169586 1.773672
H -1.754864 1.776680 2.517637
H -0.680152 3.149197 2.109210

H 2.928444 4.915856 0.608486	C 0.696781 0.670376 2.818302
H 2.656784 3.349950 -0.158091	H 1.287376 1.435924 3.342724
C 2.585886 4.708818 -2.548030	H -0.097106 0.304431 3.483468
H 3.428615 4.481613 -1.881287	C 1.579461 -0.506799 2.399697
H 2.960033 5.345440 -3.358617	V 0.000000 0.000000 0.000000
C 1.963437 3.437066 -3.080542	O 1.556816 0.116894 -1.263994
H 2.757271 2.893391 -3.609336	O 2.837966 -0.914872 -2.802822
H 1.163595 3.628114 -3.808096	O -2.509438 -2.883366 1.420522
H 1.592100 2.781213 -2.281985	O -1.288278 -1.017510 1.158184
C 0.430710 6.034147 -2.551161	O -0.926019 -0.827466 -1.484040
H -0.274430 5.195085 -2.533170	N -0.097055 -1.799671 -0.959901
H -0.029782 6.852296 -1.986174	C 1.886940 -0.903080 -2.016324
C 2.477100 6.784074 -1.293988	C 1.026511 -2.142239 -1.819206
H 3.406478 6.365078 -0.891404	H 1.614207 -2.920515 -1.317044
H 2.750804 7.308032 -2.217199	H 0.667454 -2.540678 -2.777445
C 1.814298 7.702140 -0.290784	C -0.822850 -2.875876 -0.286074
H 2.504921 8.528333 -0.066507	H -1.470335 -3.402043 -1.002780
H 1.619939 7.200539 0.666130	H -0.106760 -3.601183 0.124215
H 0.871867 8.132969 -0.644064	C -1.622444 -2.255647 0.849733
N -1.545635 -5.307919 3.702459	C 4.759854 -1.151694 0.010861
C -1.145367 -5.141481 5.158804	H 4.038914 -0.834885 -0.745503
H -0.109970 -4.779886 5.149722	H 4.194414 -1.543283 0.865262
H -1.780831 -4.349566 5.559145	C 4.314487 1.005902 1.027453
C -1.272401 -6.387275 6.000942	H 3.533143 1.014223 0.257504
H -1.274661 -6.085603 7.054988	H 3.908780 0.473798 1.894915
H -0.439616 -7.074372 5.819193	C 6.199849 0.795841 -0.607248
H -2.211278 -6.921147 5.831315	H 6.707520 1.651408 -0.149268
C -3.061897 -5.421378 3.602680	H 6.975801 0.086263 -0.919761
H -3.260969 -5.716362 2.571377	N 5.428657 0.112604 0.505430
H -3.339622 -6.265640 4.246479	C 6.415441 -0.204982 1.623564
C -3.837299 -4.168020 3.965235	H 7.337880 -0.551101 1.147833
H -4.901888 -4.405231 3.823460	H 6.634261 0.748880 2.118478
H -3.703034 -3.886805 5.017624	C 5.705991 -2.198616 -0.552585
C -1.032599 -4.080162 2.959001	H 5.973118 -1.939771 -1.589023
H 0.051208 -4.068293 3.123775	H 6.638956 -2.250538 0.024487
H -1.465157 -3.213510 3.472047	C 4.718735 2.417763 1.407200
C -1.359517 -4.043271 1.484142	H 5.036059 2.987693 0.523663
H -1.004951 -4.934584 0.950257	H 5.563387 2.424713 2.108300
C -0.980324 -6.589407 3.102717	C 5.356621 1.246191 -1.784623
H -1.452367 -7.409185 3.652798	H 4.795944 0.410841 -2.228047
H -1.336011 -6.647414 2.068869	H 4.605947 1.981575 -1.459105
C 0.529660 -6.697586 3.131072	C 5.930071 -1.235262 2.629543
H 0.782209 -7.607776 2.569244	H 4.912028 -1.009565 2.982093
H 0.948120 -6.770397 4.144787	H 5.886657 -2.228205 2.165425
H 1.008121 -5.868442 2.593772	C 6.895415 -1.297688 3.813848
H -0.867961 -3.159528 1.055447	H 7.852940 -1.698854 3.457099
H -3.599625 -3.311877 3.320668	H 7.094777 -0.269364 4.154206
H -2.436764 -3.916041 1.331353	C 6.351197 -2.162416 4.947931
V 0.000000 0.000000 0.000000	H 5.355271 -1.828368 5.275491
O 1.955542 0.762078 0.060830	H 6.240730 -3.204175 4.626820
O 3.511065 1.664198 1.438364	H 7.018663 -2.163761 5.820138
O 0.737227 -1.309151 1.270010	C 3.523386 3.116942 2.062649
O -2.830140 -1.298327 2.734183	H 2.710856 3.207868 1.326146

O -1.801242 -0.707263 0.809690
O -0.591512 1.948690 0.544368
O -1.803918 3.747438 -0.101097
O 0.078523 0.825697 -1.808893
O -1.012969 -2.722870 -2.994872
O -0.075417 -1.772925 -1.165589
N 0.462234 -0.141183 1.965785
N -1.247909 0.522450 -1.510327
C 2.466367 1.034000 1.228956
C 1.662388 0.546125 2.434528
H 2.290476 -0.135742 3.026707
H 1.362216 1.405926 3.049834
C -0.676511 -0.260868 2.866904
H -0.923479 0.743819 3.235740
H -0.435676 -0.914040 3.719900
C -1.883360 -0.804748 2.090211
C -1.460846 2.553345 -0.199905
C -2.085895 1.698586 -1.300168
H -3.076297 1.370277 -0.946210
H -2.184283 2.274864 -2.234970
C -1.794929 -0.573189 -2.291598
H -1.905501 -0.291463 -3.349251
H -2.778009 -0.810746 -1.867929
C -0.894935 -1.801035 -2.159583

H 3.137439 2.481861 2.875841
C 3.891152 4.478940 2.634466
H 4.353887 5.119494 1.874207
H 3.004668 4.987067 3.038997
H 4.619804 4.379048 3.449693
C 5.029325 -3.569657 -0.507396
H 4.876387 -3.843070 0.546985
H 4.030158 -3.497286 -0.956991
C 5.835498 -4.655618 -1.212020
H 5.884626 -4.477762 -2.295998
H 6.863432 -4.692684 -0.822467
H 5.376632 -5.643767 -1.071069
C 6.243966 1.889584 -2.853851
H 6.800965 2.735980 -2.425130
H 7.011010 1.162172 -3.174018
C 5.420425 2.362366 -4.048749
H 4.841449 1.542295 -4.499619
H 4.696241 3.127719 -3.734650
H 6.061898 2.815373 -4.814925

$[N_{3333}]_2[VBH]$

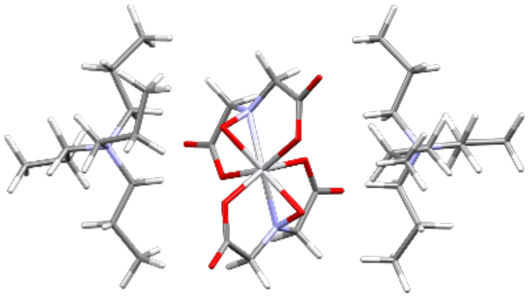

111

N -1.504272 -3.896909 3.111926
C -0.658625 -2.861321 3.826430
H -0.017351 -2.434801 3.043317
H -1.349552 -2.069432 4.139318
C -2.495559 -4.544754 4.060192
H -2.954691 -5.369520 3.499814
H -1.914983 -4.969831 4.888406
C -2.223781 -3.141868 1.995607
H -1.453061 -2.781221 1.305936
H -2.655044 -2.250547 2.466061
C -0.655907 -5.021655 2.539376
H -0.294881 -5.593244 3.405613
H -1.346033 -5.656882 1.971670
C 0.153284 -3.337749 5.016941

$[N_{3333}][VBH]$

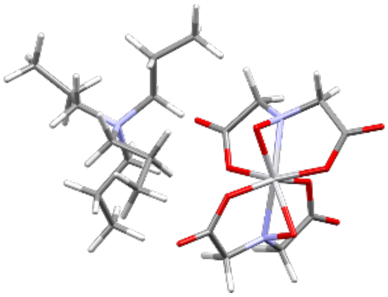

70

O -1.364021 1.445307 -0.008540
O -2.797184 2.658515 1.233678
O 2.106439 -2.213332 2.743766
O 1.152030 -1.460106 0.851761
O 1.026447 1.182830 1.145563
N 0.055916 0.616141 1.941211
C -1.808088 1.939666 1.128778
C -1.000073 1.541194 2.358959
H -1.649884 1.031059 3.081256
H -0.568169 2.446752 2.812539
C 0.565552 -0.371943 2.897583
H 1.176404 0.109251 3.675054
H -0.303346 -0.849709 3.372518
C 1.353106 -1.440428 2.136307

H -0.498328 -3.656593 5.841567	V 0.000000 0.000000 0.000000
H 0.790443 -4.191976 4.746293	O 1.624073 0.360403 -1.086271
C 1.027594 -2.166551 5.471853	O 2.974018 -0.285351 -2.759299
H 0.400293 -1.298062 5.715943	O -2.935069 -2.799898 0.263442
H 1.585224 -2.406531 6.383182	O -1.477391 -1.155500 0.711224
H 1.742052 -1.881784 4.684453	O -0.841051 -0.241449 -1.741366
C -3.295379 -3.932943 1.272409	N -0.152598 -1.405199 -1.461139
H -2.865660 -4.801048 0.753793	C 1.967960 -0.440368 -2.069243
H -4.033741 -4.313609 1.992546	C 1.039610 -1.626304 -2.273836
C -4.004821 -3.013375 0.270907	H 1.528157 -2.538588 -1.903638
H -3.448930 -2.962043 -0.671476	H 0.760676 -1.773405 -3.329598
H -5.011274 -3.385003 0.045959	C -1.026585 -2.540554 -1.178931
H -4.117586 -1.991270 0.659732	H -1.619098 -2.782601 -2.071264
C 0.501897 -4.617387 1.633460	H -0.412479 -3.404725 -0.887779
H 0.242328 -3.745626 1.017284	C -1.909505 -2.173406 0.004818
H 0.581635 -5.454551 0.925565	C 4.665110 -1.123345 0.338617
C 1.847028 -4.387352 2.323519	H 3.732455 -0.731480 -0.080879
H 2.088043 -5.213038 3.010737	H 4.391717 -1.741483 1.203053
H 2.653069 -4.329651 1.579884	C 4.409069 0.700988 1.879828
H 1.871406 -3.448731 2.890207	H 3.501868 0.885521 1.290584
C -3.557896 -3.609804 4.604359	H 4.181359 -0.085679 2.609827
H -4.071470 -3.083286 3.789264	C 5.820993 1.057982 -0.172747
H -3.084835 -2.847072 5.234901	H 6.482609 1.789723 0.308412
C -4.570829 -4.403100 5.425914	H 6.428984 0.463534 -0.866028
H -5.362951 -3.745612 5.803763	N 5.401730 0.077976 0.911900
H -5.048206 -5.181008 4.809269	C 6.691092 -0.326317 1.608290
H -4.075374 -4.871657 6.286090	H 7.420119 -0.537527 0.817383
V 0.000000 0.000000 0.000000	H 7.023787 0.574041 2.138638
O 1.974899 0.185032 -0.586517	C 5.404368 -1.953394 -0.690499
O 4.105212 0.433218 0.097839	H 5.603236 -1.360354 -1.593229
O 0.798810 -1.247334 1.337868	H 6.369072 -2.300311 -0.296677
O -2.186507 0.047198 3.501040	C 4.826002 1.966469 2.603501
O -1.589682 -0.078264 1.329145	H 5.137165 2.746294 1.894422
O -0.090743 2.076396 0.350622	H 5.665341 1.767374 3.281544
O -1.292826 3.936800 -0.074670	C 4.684945 1.754141 -0.897026
O -0.220840 0.619544 -1.870830	H 3.816943 1.099989 -1.012812
O -1.775594 -2.788890 -2.451177	H 4.371048 2.627629 -0.315584
O -0.575771 -1.818337 -0.805484	C 6.601222 -1.518651 2.537674
N 0.966795 0.058567 1.784904	H 5.736545 -1.433709 3.205731
N -1.472207 0.554801 -1.268172	H 6.507221 -2.436278 1.945687
C 2.884746 0.332989 0.316288	C 7.879612 -1.573393 3.374849
C 2.367976 0.468501 1.757339	H 8.760342 -1.639904 2.723266
H 2.977633 -0.134187 2.448096	H 7.950316 -0.671817 4.001308
H 2.433932 1.526643 2.051545	H 7.878088 -2.442203 4.043815
C 0.122359 0.417559 2.925678	C 3.610707 2.426174 3.419438
H 0.225400 1.500367 3.085836	H 2.764054 2.666121 2.759922
H 0.428478 -0.115765 3.836778	H 3.284520 1.640215 4.115766
C -1.350294 0.108499 2.582583	H 3.843871 3.313413 4.017300
C -1.086056 2.711026 -0.176856	C 4.511670 -3.141968 -1.058264
C -2.072744 1.856410 -0.985302	H 4.230739 -3.729509 -0.173252
H -2.976448 1.671097 -0.387888	H 3.589951 -2.787796 -1.537737
H -2.345154 2.371575 -1.918723	H 5.012441 -3.820396 -1.756363
C -2.294994 -0.516701 -1.823346	C 5.151876 2.186221 -2.285896

H -2.556107 -0.291780 -2.867308
H -3.217208 -0.587301 -1.226112
C -1.495898 -1.823026 -1.717322
N 2.680938 4.018939 -2.153212
C 2.949019 3.633339 -0.704764
H 2.887391 2.542103 -0.683679
H 2.097209 4.018212 -0.131396
C 3.631070 3.293160 -3.091768
H 4.639564 3.632992 -2.822057
H 3.409448 3.685436 -4.093060
C 1.231174 3.643680 -2.423841
H 1.118103 2.590728 -2.136673
H 0.631235 4.229962 -1.717850
C 2.913151 5.498108 -2.388599
H 3.982686 5.669230 -2.211633
H 2.730160 5.666749 -3.456935
C 4.259796 4.135246 -0.130698
H 4.252434 5.230718 -0.084111
H 5.117869 3.854495 -0.754840
C 4.472170 3.566035 1.273556
H 5.423896 3.932726 1.677126
H 3.662361 3.878761 1.950216
H 4.513730 2.468687 1.251783
C 0.750399 3.873576 -3.845631
H 1.318240 3.248120 -4.550425
H 0.892164 4.924242 -4.141830
C -0.735898 3.515964 -3.924698
H -1.127007 3.620311 -4.943646
H -0.885842 2.469633 -3.627601
H -1.314941 4.157959 -3.244369
C 3.549597 1.779881 -3.087391
H 3.899966 1.364946 -2.132067
H 2.509929 1.449241 -3.200743
C 4.388719 1.236255 -4.243817
H 4.019235 1.594009 -5.216577
H 5.443075 1.540793 -4.180771
H 4.349337 0.141686 -4.251972
C 2.085873 6.453835 -1.556546
H 2.308079 6.316495 -0.490702
H 1.006650 6.294819 -1.687444
C 2.433525 7.871790 -1.997351
H 3.510195 8.067557 -1.916041
H 2.143218 8.002383 -3.046856
H 1.895740 8.611045 -1.400615
H 6.072919 2.779971 -2.227233
H 5.342655 1.303863 -2.914212
H 4.392451 2.792477 -2.796165

$[N_{4444}]_2[VBH]$

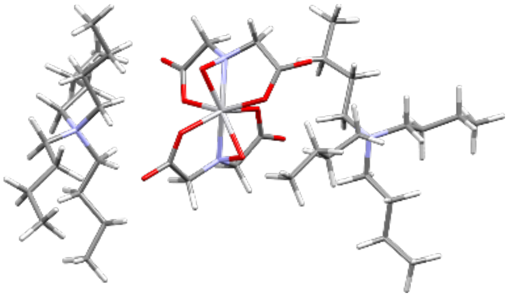

135

N -4.053854 -3.482146 2.060654
C -2.568862 -3.205813 2.258109
H -2.073070 -3.586712 1.355943
H -2.453379 -2.114847 2.249396
C -4.911036 -2.935256 3.195314
H -5.934399 -2.911706 2.796183
H -4.868686 -3.672651 4.005442
C -4.456901 -2.793895 0.750421
H -3.604589 -2.928909 0.079165
H -4.550334 -1.727730 0.987458
C -4.304829 -4.974714 1.988428
H -4.000010 -5.382678 2.959078
H -5.393582 -5.086014 1.917582
C -1.958816 -3.826706 3.502244
H -2.478973 -3.491629 4.412952
H -2.039811 -4.925055 3.467967
C -0.475858 -3.453689 3.603736
H -0.382062 -2.365624 3.724927
H 0.021933 -3.691052 2.649733
C -5.707698 -3.324011 0.070238
H -5.667034 -4.419196 -0.005478
H -6.627459 -3.050646 0.604153
C -5.772798 -2.778830 -1.363936
H -4.754106 -2.750822 -1.785607
H -6.146905 -1.748361 -1.351327
C -3.608002 -5.666519 0.821805
H -2.588264 -5.963161 1.107382
H -3.481868 -4.970156 -0.017438
C -4.391910 -6.889979 0.332727
H -5.468201 -6.649209 0.315056
H -4.275271 -7.727521 1.037874
C -4.499415 -1.565211 3.696220
H -4.229253 -0.900100 2.865245
H -3.595763 -1.630360 4.317120
C -5.630442 -0.917159 4.496478
H -6.506858 -0.834190 3.837284
H -5.929473 -1.577866 5.327301
C -3.952385 -7.318080 -1.065801
H -4.423888 -8.266428 -1.358002

$[N_{4444}][VBH]$

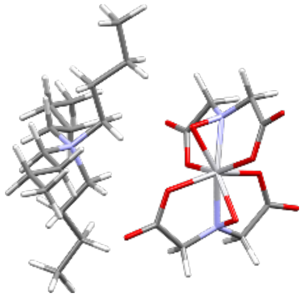

82

O -1.455835 1.286909 -0.402973
O -2.518055 3.192114 0.162914
O 2.441800 -0.938390 3.171949
O 1.339994 -0.977398 1.206365
O 0.947333 1.571508 0.626004
N 0.060530 1.226036 1.624322
C -1.740396 2.277050 0.418067
C -1.045768 2.169586 1.773672
H -1.754864 1.776680 2.517637
H -0.680152 3.149197 2.109210
C 0.696781 0.670376 2.818302
H 1.287376 1.435924 3.342724
H -0.097106 0.304431 3.483468
C 1.579461 -0.506799 2.399697
V 0.000000 0.000000 0.000000
O 1.556816 0.116894 -1.263994
O 2.837966 -0.914872 -2.802822
O -2.509438 -2.883366 1.420522
O -1.288278 -1.017510 1.158184
O -0.926019 -0.827466 -1.484040
N -0.097055 -1.799671 -0.959901
C 1.886940 -0.903080 -2.016324
C 1.026511 -2.142239 -1.819206
H 1.614207 -2.920515 -1.317044
H 0.667454 -2.540678 -2.777445
C -0.822850 -2.875876 -0.286074
H -1.470335 -3.402043 -1.002780
H -0.106760 -3.601183 0.124215
C -1.622444 -2.255647 0.849733
C 4.759854 -1.151694 0.010861
H 4.038914 -0.834885 -0.745503
H 4.194414 -1.543283 0.865262
C 4.314487 1.005902 1.027453
H 3.533143 1.014223 0.257504
H 3.908780 0.473798 1.894915
C 6.199849 0.795841 -0.607248
H 6.707520 1.651408 -0.149268
H 6.975801 0.086263 -0.919761
N 5.428657 0.112604 0.505430

H -4.201459 -6.536505 -1.799453	C 6.415441 -0.204982 1.623564
H -2.862061 -7.457564 -1.091547	H 7.337880 -0.551101 1.147833
C 0.205570 -4.180807 4.761311	H 6.634261 0.748880 2.118478
H 1.240825 -3.843595 4.898743	C 5.705991 -2.198616 -0.552585
H -0.327163 -4.007126 5.707391	H 5.973118 -1.939771 -1.589023
H 0.223780 -5.268302 4.588384	H 6.638956 -2.250538 0.024487
C -6.650412 -3.659265 -2.245510	C 4.718735 2.417763 1.407200
H -6.756989 -3.224638 -3.248235	H 5.036059 2.987693 0.523663
H -6.201551 -4.659574 -2.357694	H 5.563387 2.424713 2.108300
H -7.653113 -3.778221 -1.816019	C 5.356621 1.246191 -1.784623
C -5.213388 0.451123 5.038316	H 4.795944 0.410841 -2.228047
H -4.676191 1.022710 4.270954	H 4.605947 1.981575 -1.459105
H -6.089200 1.035791 5.354416	C 5.930071 -1.235262 2.629543
H -4.542330 0.345330 5.901011	H 4.912028 -1.009565 2.982093
V 0.000000 0.000000 0.000000	H 5.886657 -2.228205 2.165425
O 1.944505 0.492016 -0.572351	C 6.895415 -1.297688 3.813848
O 3.612302 1.938959 -0.113939	H 7.852940 -1.698854 3.457099
O 0.909736 -0.757694 1.590272	H 7.094777 -0.269364 4.154206
O -2.409024 0.709696 3.280311	C 6.351197 -2.162416 4.947931
O -1.652562 0.011968 1.274313	H 5.355271 -1.828368 5.275491
O -0.353122 2.046318 -0.218575	H 6.240730 -3.204175 4.626820
O -1.530342 3.614676 -1.342096	H 7.018663 -2.163761 5.820138
O -0.146525 -0.010238 -1.962064	C 3.523386 3.116942 2.062649
O -1.831714 -3.532121 -1.173592	H 2.710856 3.207868 1.326146
O -0.429384 -2.028295 -0.247100	H 3.137439 2.481861 2.875841
N 0.850133 0.621266 1.753389	C 3.891152 4.478940 2.634466
N -1.436218 0.032727 -1.442158	H 4.353887 5.119494 1.874207
C 2.627610 1.253548 0.213412	H 3.004668 4.987067 3.038997
C 2.153979 1.282508 1.668351	H 4.619804 4.379048 3.449693
H 2.890401 0.772520 2.304661	C 5.029325 -3.569657 -0.507396
H 2.062437 2.318403 2.015706	H 4.876387 -3.843070 0.546985
C -0.097807 1.055333 2.777792	H 4.030158 -3.497286 -0.956991
H -0.110103 2.154809 2.773400	C 5.835498 -4.655618 -1.212020
H 0.198327 0.701658 3.778356	H 5.884626 -4.477762 -2.295998
C -1.509932 0.556558 2.434515	H 6.863432 -4.692684 -0.822467
C -1.279989 2.433715 -1.045381	H 5.376632 -5.643767 -1.071069
C -2.110621 1.311790 -1.656594	C 6.243966 1.889584 -2.853851
H -3.084467 1.296427 -1.141537	H 6.800965 2.735980 -2.425130
H -2.270326 1.488651 -2.727224	H 7.011010 1.162172 -3.174018
C -2.178502 -1.204453 -1.655397	C 5.420425 2.362366 -4.048749
H -2.324995 -1.397796 -2.728705	H 4.841449 1.542295 -4.499619
H -3.166312 -1.097075 -1.185572	H 4.696241 3.127719 -3.734650
C -1.424236 -2.364080 -0.993047	H 6.061898 2.815373 -4.814925
N 2.239586 4.161469 -3.327329	
C 1.482330 4.220527 -1.997020	
H 1.177220 3.192433 -1.774611	
H 0.567454 4.794893 -2.172584	
C 3.141081 2.920057 -3.334328	
H 3.631268 2.898670 -2.351607	
H 3.902695 3.098015 -4.105686	
C 1.200010 4.089182 -4.435258	
H 0.614671 3.182463 -4.248363	
H 0.532377 4.944578 -4.282123	

C 3.122770 5.377680 -3.517612<br>H 3.817728 5.389965 -2.669899<br>H 3.712945 5.189684 -4.422304<br>C 2.250811 4.808632 -0.826309<br>H 2.480300 5.871180 -1.008081<br>H 3.200009 4.276718 -0.666022<br>C 1.389807 4.702803 0.436694<br>H 0.370085 5.045179 0.217754<br>H 1.272774 3.644467 0.690297<br>C 1.776530 4.099215 -5.837763<br>H 2.472423 3.259260 -5.966410<br>H 2.332740 5.033068 -6.017080<br>C 0.672345 3.977249 -6.888672<br>H 0.213562 2.983005 -6.800246<br>H -0.112405 4.723997 -6.678091<br>C 2.404861 1.623555 -3.612977<br>H 1.591669 1.463948 -2.890848<br>H 1.978643 1.628292 -4.626110<br>C 3.349052 0.425482 -3.517815<br>H 4.266407 0.617810 -4.099732<br>H 3.645498 0.289430 -2.470263<br>C 2.363237 6.685544 -3.652295<br>H 1.713255 6.853086 -2.781809<br>H 1.708363 6.647659 -4.535563<br>C 3.313335 7.871287 -3.821104<br>H 3.938275 7.985435 -2.922843<br>H 4.002974 7.664894 -4.658023<br>C 2.655280 -0.825795 -4.044771<br>H 3.297189 -1.710900 -3.951841<br>H 1.733780 -1.005979 -3.474550<br>H 2.382356 -0.688979 -5.100692<br>C 1.986708 5.494100 1.596186<br>H 2.033698 6.568099 1.355172<br>H 1.384985 5.381235 2.510982<br>H 3.009660 5.158791 1.815867<br>C 1.217211 4.163288 -8.302082<br>H 2.044105 3.468380 -8.503331<br>H 0.452557 3.956475 -9.060283<br>H 1.597893 5.185451 -8.450956<br>C 2.538944 9.161357 -4.082548<br>H 1.936777 9.453882 -3.210489<br>H 3.221783 9.992932 -4.296771<br>H 1.861269 9.032809 -4.940881	

Monosubstituted [N111x]	
**[N_{1112}]$_2$[VBH]**	**[N_{1112}][VBH]**
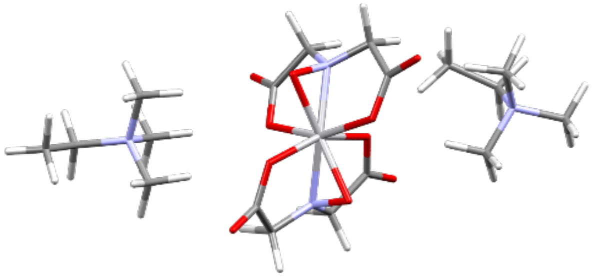	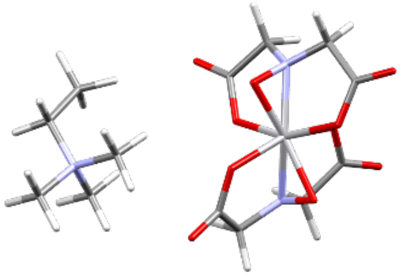
69	49
N -4.630079 -2.336323 1.943002	O -1.601051 1.181531 0.088662
C -3.411992 -2.960426 2.558066	O -3.261059 1.984687 1.377969
H -3.267350 -3.948570 2.109127	O 2.407902 -2.109093 2.513877
H -2.559160 -2.314146 2.317402	O 1.385883 -1.316329 0.671421
C -4.793716 -0.944474 2.492464	O 0.691244 1.082182 1.455172
H -5.671391 -0.502192 2.006268	N -0.219414 0.203886 1.992246
H -4.943347 -1.026387 3.573118	C -2.186389 1.398692 1.246167
C -4.448198 -2.245913 0.457379	C -1.457515 0.827940 2.450311
H -4.296018 -3.251005 0.052945	H -2.063815 0.058530 2.945260
H -3.549964 -1.642142 0.291000	H -1.221839 1.641988 3.148457
C -5.883304 -3.119970 2.303224	C 0.341796 -0.906780 2.767438
H -5.946402 -3.068471 3.393678	H 0.627614 -0.587610 3.779572
H -6.708971 -2.528596 1.887890	H -0.449756 -1.665707 2.850102
H -3.883036 -0.379073 2.266029	C 1.487912 -1.496306 1.966618
H -5.335067 -1.753879 0.040455	V 0.000000 0.000000 0.000000
H -3.562169 -3.005807 3.644216	O 1.483770 0.985773 -0.886691
C -5.932873 -4.555560 1.822158	O 2.708824 1.210059 -2.755987
H -5.120422 -5.164840 2.235598	O -2.395520 -3.182399 -0.702340
H -6.883419 -4.972861 2.184982	O -1.300345 -1.512524 0.325297
H -5.894880 -4.638829 0.729592	O -0.693515 -0.136284 -1.828485
V 0.000000 0.000000 0.000000	N 0.268064 -1.110450 -1.666942
O 1.975835 -0.183989 -0.610779	C 1.949578 0.563361 -2.033636
O 4.113246 -0.373195 0.096176	C 1.500753 -0.849901 -2.406601
O 0.591647 -1.326905 1.359798	H 2.255766 -1.577388 -2.074542
O -2.105275 0.871863 3.416222	H 1.343046 -0.942608 -3.489844
O -1.615622 0.161152 1.332447	C -0.255786 -2.476622 -1.581589
O 0.204045 2.025399 0.396802	H -0.549031 -2.847335 -2.572657
O -0.686705 4.059961 0.046697	H 0.530399 -3.123234 -1.160249
O -0.161799 0.719620 -1.851744	C -1.423788 -2.426003 -0.598454
O -2.088155 -2.456942 -2.535180	C 5.642796 -2.143384 -0.975081
O -0.765053 -1.693443 -0.878483	H 5.242209 -1.899237 -1.964678
N 0.967013 -0.058473 1.773498	H 4.901669 -2.695647 -0.392900
N -1.401695 0.789132 -1.235513	C 4.714684 -0.041207 -0.131319
C 2.903880 -0.170173 0.293751	H 4.371314 0.231008 -1.134124
C 2.416446 0.148686 1.719337	H 3.953758 -0.632508 0.388576
H 2.949369 -0.479604 2.447997	C 6.958398 -0.082844 -1.098563
H 2.615711 1.205476 1.950672	H 7.175323 0.851400 -0.574678

C 0.184135 0.452857 2.900961
H 0.533010 1.468667 3.130017
H 0.309190 -0.182498 3.787561
C -1.304917 0.507755 2.537845
C -0.682645 2.829525 -0.097654
C -1.797537 2.162072 -0.912734
H -2.713558 2.127068 -0.305588
H -1.978016 2.749357 -1.820350
C -2.352383 -0.165345 -1.814295
H -2.615669 0.096532 -2.848929
H -3.267237 -0.161174 -1.203802
C -1.701113 -1.564559 -1.768685
N 3.577980 4.450754 0.044443
C 2.900798 5.053629 1.268317
H 2.223192 5.814643 0.867012
C 4.431104 3.278328 0.421650
H 5.082223 3.582549 1.249685
H 4.994413 2.978409 -0.468390
C 2.547532 4.033236 -0.968659
H 1.899563 3.281012 -0.510224
H 1.968392 4.922002 -1.246909
C 4.457232 5.502654 -0.571261
H 5.220163 5.782625 0.163467
H 4.911304 5.063872 -1.466848
H 3.082117 3.619422 -1.829728
H 3.833333 6.368288 -0.811583
H 3.697988 5.546004 1.832480
H 3.783081 2.457342 0.734958
C 2.192787 4.050805 2.156035
H 1.445542 3.458678 1.613398
H 2.915677 3.406901 2.673258
H 1.671844 4.623697 2.935870

$[N_{1113}]_2[VBH]$

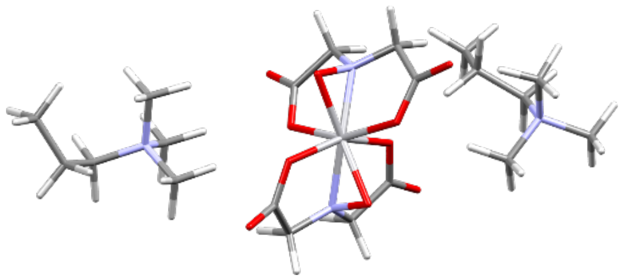

75

N -5.033306 -2.041165 1.828593
C -4.027461 -2.814615 2.624523
H -3.810749 -3.744251 2.088366
H -3.124803 -2.196162 2.692432
C -5.248451 -0.707592 2.491479
H -5.990383 -0.161443 1.896654
H -5.619025 -0.889246 3.504359

H 7.868263 -0.682441 -1.194449
N 5.964569 -0.856208 -0.280626
C 6.595950 -1.128738 1.080254
H 7.570952 -1.585510 0.863698
H 6.764114 -0.132657 1.509996
H 6.561603 -2.730623 -1.068516
H 4.963973 0.876036 0.411099
H 6.527274 0.129584 -2.082352
C 5.758062 -2.007145 1.984647
H 5.715825 -3.047036 1.636916
H 6.214749 -2.004634 2.980426
H 4.724631 -1.660382 2.107537

$[N_{1113}][VBH]$

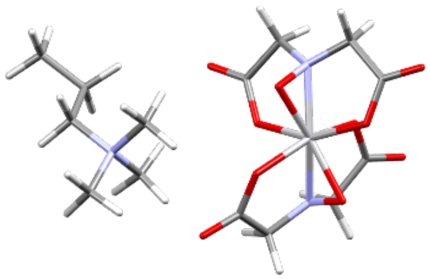

52

O -1.582383 1.206089 0.072820
O -3.242701 2.012902 1.355079
O 2.464927 -2.033332 2.544567
O 1.371132 -1.317484 0.725078
O 0.720971 1.171540 1.367339
N -0.156086 0.319676 1.999309
C -2.158144 1.450670 1.232168

C -4.486041 -1.797078 0.454026
H -4.259543 -2.755964 -0.021648
H -3.563429 -1.216251 0.577393
C -6.378916 -2.755622 1.759136
H -6.769559 -2.739708 2.783614
H -6.990980 -2.085900 1.142327
H -4.281754 -0.188619 2.517887
H -5.242752 -1.237758 -0.108330
H -4.434196 -2.986364 3.627884
C -6.357722 -4.175582 1.194309
H -5.564665 -4.284131 0.441890
H -7.308689 -4.304498 0.655314
C -6.236460 -5.277451 2.250333
H -6.268355 -6.275882 1.790701
H -5.289662 -5.209819 2.799909
H -7.059551 -5.213018 2.976274
V 0.000000 0.000000 0.000000
O 1.978869 -0.129849 -0.603497
O 4.127261 -0.190439 0.084223
O 0.588441 -1.368802 1.311917
O -2.153362 0.504658 3.478666
O -1.619329 0.117829 1.323395
O 0.204207 2.014697 0.434691
O -0.606482 4.079997 0.062682
O -0.122784 0.737720 -1.841297
O -2.071232 -2.388210 -2.607292
O -0.781914 -1.665963 -0.908361
N 0.960153 -0.118044 1.778464
N -1.375538 0.829186 -1.250410
C 2.904535 -0.103814 0.297767
C 2.410954 0.078835 1.744781
H 2.929435 -0.626183 2.412692
H 2.632789 1.103083 2.072678
C 0.166454 0.338330 2.922390
H 0.464302 1.370385 3.156476
H 0.336038 -0.297979 3.801317
C -1.329361 0.320840 2.565317
C -0.636138 2.849575 -0.086150
C -1.746917 2.212250 -0.939453
H -2.680175 2.201492 -0.357800
H -1.892058 2.800633 -1.854425
C -2.325773 -0.102436 -1.862939
H -2.561316 0.179119 -2.900065
H -3.255020 -0.093929 -1.275396
C -1.692933 -1.513029 -1.817494
N 3.780273 4.209818 0.059743
C 3.086137 4.748023 1.300319
H 2.347529 5.469796 0.931799
C 4.738613 3.110089 0.409143
H 5.373975 3.461659 1.230817
H 5.310060 2.873173 -0.494977
C 2.765279 3.718765 -0.934345
H 2.177517 2.919156 -0.473866

C -1.405359 0.932838 2.450422
H -2.009004 0.164567 2.951003
H -1.207164 1.763226 3.140618
C 0.501079 -0.673963 2.853965
H 0.967684 -0.185485 3.723107
H -0.254304 -1.399966 3.188583
C 1.538320 -1.408058 2.009485
V 0.000000 0.000000 0.000000
O 1.586854 0.805620 -0.924840
O 3.229544 0.482535 -2.438843
O -2.519610 -3.121934 -0.409551
O -1.295947 -1.447714 0.450805
O -0.711210 -0.173790 -1.816196
N 0.181298 -1.207143 -1.617192
C 2.164459 0.162154 -1.913556
C 1.425486 -1.091209 -2.366660
H 2.034109 -1.978909 -2.154767
H 1.222589 -1.047199 -3.444065
C -0.446200 -2.527028 -1.489354
H -0.836083 -2.856832 -2.463276
H 0.318902 -3.229574 -1.123729
C -1.524033 -2.399482 -0.428334
C 4.723181 -1.983603 -0.235152
H 4.127821 -1.635177 -1.084924
H 4.071679 -2.399742 0.539658
C 4.459857 0.179633 0.903751
H 3.766858 0.500713 0.122417
H 3.920386 -0.340586 1.699415
C 6.186217 -0.110894 -0.800755
H 6.748574 0.726034 -0.378128
H 6.865498 -0.849764 -1.240503
N 5.444516 -0.788341 0.316365
C 6.453452 -1.197412 1.379025
H 7.330973 -1.571114 0.844272
H 6.718221 -0.264381 1.894372
H 5.463175 -2.724280 -0.557031
H 5.019428 1.045218 1.272284
H 5.446215 0.253550 -1.521657
C 5.983563 -2.259605 2.355561
H 5.829737 -3.211564 1.831649
H 5.015443 -1.999606 2.804647
C 7.061726 -2.434456 3.427702
H 6.753732 -3.168797 4.182369
H 8.006450 -2.776751 2.979797
H 7.268330 -1.480501 3.938616

H 2.117020 4.563831 -1.196400
C 4.556002 5.329533 -0.574011
H 5.296459 5.683982 0.150773
H 5.037408 4.925183 -1.471197
H 3.314383 3.351517 -1.806218
H 3.858272 6.135480 -0.817875
H 3.857343 5.282985 1.863104
H 4.179036 2.225014 0.718021
C 2.460562 3.689326 2.186975
H 1.716243 3.105879 1.628993
H 3.253220 3.030796 2.566790
C 1.802215 4.363583 3.388893
H 1.025986 5.069564 3.074990
H 1.341126 3.621177 4.055730
H 2.556480 4.908740 3.968947

$[N_{1114}]_2[VBH]$

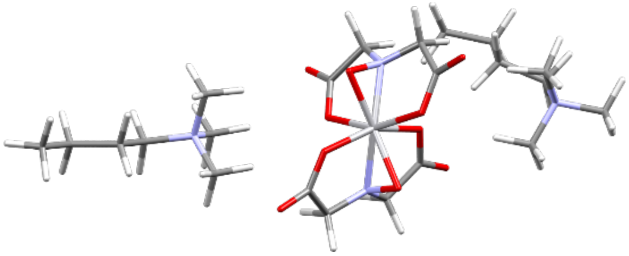

81

N -5.062383 -2.055894 1.962973
C -3.858208 -2.651295 2.623982
H -3.619164 -3.590304 2.115925
H -3.038102 -1.934774 2.501044
C -5.323194 -0.705181 2.565478
H -6.172393 -0.263416 2.030536
H -5.567244 -0.851959 3.622602
C -4.780696 -1.892521 0.501532
H -4.561975 -2.874268 0.069034
H -3.898825 -1.247270 0.422117
C -6.292610 -2.910348 2.200382
H -6.431479 -2.911930 3.285836
H -7.122049 -2.350928 1.746799
H -4.411558 -0.106502 2.453312
H -5.657757 -1.429409 0.037221
H -4.078422 -2.800276 3.687967
C -6.232696 -4.331041 1.673958
H -5.337067 -4.849171 2.042836
H -6.170614 -4.348866 0.577372
C -7.476839 -5.077821 2.157939
H -7.510105 -5.051637 3.255947
H -8.381812 -4.539350 1.833416
C -7.528845 -6.523546 1.677572
H -6.621729 -7.076879 1.960192

$[N_{1114}][VBH]$

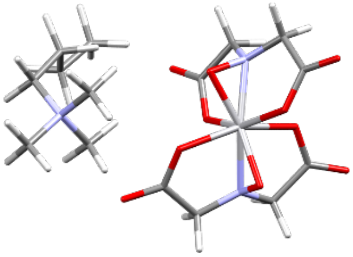

55

O -1.538628 1.294994 0.140619
O -3.149753 2.090952 1.499509
O 2.487401 -2.093627 2.411402
O 1.267625 -1.418111 0.653367
O 0.814931 1.098874 1.354280
N -0.094547 0.294617 2.003147
C -2.074077 1.515562 1.316695
C -1.292842 0.965333 2.501295
H -1.902849 0.211002 3.015890
H -1.046781 1.779845 3.198858
C 0.505356 -0.772536 2.810363
H 0.950226 -0.385662 3.734752
H -0.289481 -1.488619 3.056810
C 1.524711 -1.490702 1.935952
V 0.000000 0.000000 0.000000
O 1.645502 0.705547 -0.933925
O 3.314880 0.172025 -2.344735
O -2.757242 -2.945890 -0.404869
O -1.428835 -1.342563 0.437249
O -0.708976 -0.048716 -1.810945
N 0.065399 -1.178237 -1.652054
C 2.188866 -0.016595 -1.878689
C 1.333392 -1.178812 -2.380839
H 1.844856 -2.124092 -2.154262

H	-8.385438	-7.048579	2.120898
H	-7.646004	-6.576526	0.583953
V	0.000000	0.000000	0.000000
O	1.986001	-0.194155	-0.606174
O	4.110836	-0.438720	0.126893
O	0.576591	-1.357648	1.333989
O	-2.159047	0.582228	3.467589
O	-1.618099	0.149934	1.326215
O	0.262960	2.020677	0.394873
O	-0.566515	4.087369	0.061100
O	-0.164647	0.699754	-1.857202
O	-2.412928	-2.447970	-2.257508
O	-0.839738	-1.675080	-0.843986
N	0.954884	-0.101711	1.780462
N	-1.389581	0.832450	-1.222990
C	2.900860	-0.216610	0.310863
C	2.405687	0.092744	1.735667
H	2.931049	-0.547641	2.458545
H	2.614378	1.145931	1.973174
C	0.167060	0.373072	2.921603
H	0.474061	1.402480	3.148723
H	0.332501	-0.256808	3.805988
C	-1.327728	0.369006	2.564615
C	-0.595511	2.858920	-0.097725
C	-1.728658	2.226040	-0.919155
H	-2.645895	2.233468	-0.315371
H	-1.889660	2.807992	-1.835496
C	-2.397277	-0.092143	-1.751809
H	-2.642405	0.126731	-2.801378
H	-3.312170	0.012880	-1.151770
C	-1.862341	-1.533979	-1.629970
N	3.817512	4.289014	0.026480
C	3.226834	4.885394	1.294433
H	2.617729	5.731093	0.957586
C	4.570900	3.032154	0.331811
H	5.235039	3.236691	1.175884
H	5.128393	2.741536	-0.564650
C	2.741126	4.000455	-0.980875
H	2.048765	3.270619	-0.549420
H	2.216525	4.936407	-1.208871
C	4.769665	5.292214	-0.562384
H	5.569657	5.469906	0.164785
H	5.175440	4.861454	-1.484612
H	3.235896	3.593445	-1.869433
H	4.218973	6.219340	-0.745038
H	4.077947	5.266480	1.870333
H	3.857965	2.248600	0.593781
C	2.423208	3.926752	2.150215
H	1.591023	3.494538	1.577655
H	3.071403	3.115799	2.514262
C	1.884340	4.675832	3.374565
H	2.731045	4.889277	4.039498
H	1.485863	5.647687	3.057188
H	1.182162	-1.095362	-3.469785
C	-0.695892	-2.430505	-1.549180
H	-1.169407	-2.682450	-2.507064
H	0.003341	-3.225044	-1.243037
C	-1.732579	-2.263162	-0.443091
C	4.848129	-1.940420	0.005827
H	4.270093	-1.650709	-0.877958
H	4.179790	-2.332213	0.779438
C	4.501025	0.317615	0.921552
H	3.854640	0.526386	0.065135
H	3.912218	-0.085567	1.749005
C	6.341302	-0.114687	-0.604613
H	6.851659	0.776905	-0.227640
H	7.057140	-0.880820	-0.908895
N	5.522886	-0.696992	0.510278
C	6.461626	-0.990741	1.673147
H	7.312964	-1.531393	1.238470
H	6.804860	0.001361	1.991545
H	5.624432	-2.665094	-0.261318
H	5.035426	1.228509	1.204008
H	5.660219	0.150091	-1.420610
C	5.836850	-1.787365	2.815061
H	4.756331	-1.595737	2.891007
H	6.279471	-1.434171	3.759261
C	6.079099	-3.294272	2.707834
H	5.767851	-3.659102	1.722275
H	7.162458	-3.473504	2.789196
C	5.334199	-4.060864	3.789289
H	4.254321	-3.876567	3.706923
H	5.506791	-5.143540	3.711189
H	5.675811	-3.735251	4.777209

C 0.808064 3.884697 4.114728<br>H 0.003191 3.585469 3.425296<br>H 1.236481 2.971611 4.557502<br>H 0.354604 4.476875 4.922170	

Symmetric disubstituted [N11xx]

$[N_{1122}]_2[VBH]$	$[N_{1122}][VBH]$
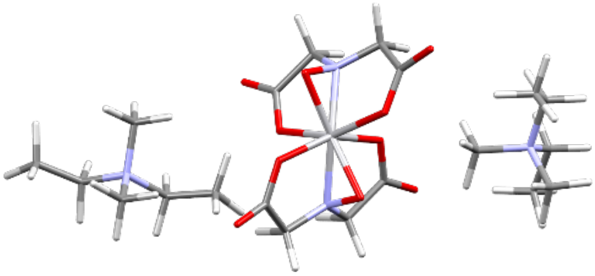	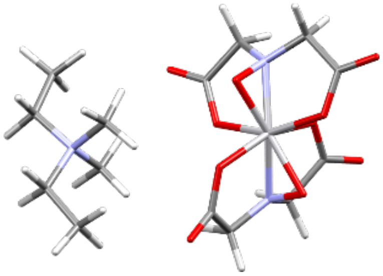
75	52
N -3.932092 -3.130003 2.484382<br>C -3.431626 -4.218362 3.374430<br>H -2.698394 -4.813722 2.824640<br>H -2.976353 -3.786631 4.266750<br>C -4.912435 -2.239646 3.242970<br>H -5.697551 -2.903142 3.623991<br>C -2.797745 -2.302140 1.968783<br>H -2.191960 -2.903576 1.290125<br>H -2.193006 -1.959743 2.812242<br>C -4.703493 -3.739992 1.323764<br>H -5.605526 -4.154775 1.792857<br>H -4.988898 -2.891848 0.688318<br>H -5.324790 -1.571353 2.474740<br>H -3.206608 -1.435255 1.440491<br>H -4.284791 -4.825886 3.686811<br>C -3.963239 -4.803689 0.535146<br>H -3.726536 -5.690855 1.134249<br>H -4.618534 -5.128520 -0.283130<br>H -3.057259 -4.418555 0.054001<br>C -4.286849 -1.440646 4.365619<br>H -5.118078 -0.970568 4.906427<br>H -3.766200 -2.080904 5.090412<br>H -3.611649 -0.660293 3.993801<br>V 0.000000 0.000000 0.000000<br>O 1.992401 -0.343605 -0.465942<br>O 4.097012 -0.439437 0.341193<br>O 0.500808 -1.010151 1.632680<br>O -2.216122 1.128409 3.295270<br>O -1.609262 0.538608 1.209547<br>O 0.387723 2.030433 -0.187883<br>O -0.229708 3.929061 -1.222867<br>O -0.158371 0.124729 -1.997870	O -1.597720 1.181357 0.136710<br>O -3.223961 1.969430 1.474689<br>O 2.518480 -2.075720 2.442000<br>O 1.408313 -1.312603 0.643443<br>O 0.737994 1.115891 1.411817<br>N -0.137024 0.232988 2.000475<br>C -2.147349 1.396095 1.312290<br>C -1.372593 0.835555 2.495686<br>H -1.950500 0.053579 3.007064<br>H -1.138801 1.648374 3.194427<br>C 0.489674 -0.831076 2.785814<br>H 0.874024 -0.439075 3.736988<br>H -0.280848 -1.584204 3.003628<br>C 1.573699 -1.464009 1.930630<br>V 0.000000 0.000000 0.000000<br>O 1.456193 0.954690 -0.955055<br>O 2.484739 1.233609 -2.934126<br>O -2.443067 -3.157059 -0.538825<br>O -1.269955 -1.498193 0.412664<br>O -0.773113 -0.158989 -1.793656<br>N 0.193552 -1.133964 -1.667084<br>C 1.831647 0.553457 -2.147015<br>C 1.384689 -0.876051 -2.475549<br>H 2.163314 -1.592391 -2.175517<br>H 1.170281 -0.986234 -3.547521<br>C -0.334397 -2.496331 -1.532477<br>H -0.683740 -2.882113 -2.500015<br>H 0.466439 -3.141712 -1.137306<br>C -1.457157 -2.419377 -0.495775<br>C 4.765557 -2.127195 -0.083910<br>H 4.139860 -1.882844 -0.945906<br>H 4.128986 -2.486959 0.729038

O	-2.596479	-2.799630	-1.585232
O	-0.936959	-1.822349	-0.409675
N	0.909729	0.312706	1.779154
N	-1.344976	0.535134	-1.399816
C	2.884753	-0.163209	0.453953
C	2.362977	0.462314	1.753519
H	2.812144	0.010594	2.648896
H	2.593800	1.538126	1.750041
C	0.127824	1.058920	2.769810
H	0.405444	2.120883	2.689088
H	0.370754	0.714041	3.784205
C	-1.354719	0.899214	2.424832
C	-0.391636	2.724238	-0.949083
C	-1.595747	1.970766	-1.528099
H	-2.484988	2.210891	-0.926260
H	-1.769229	2.245920	-2.580125
C	-2.454026	-0.397067	-1.576187
H	-2.833517	-0.371107	-2.605672
H	-3.260511	-0.107265	-0.883915
C	-1.974323	-1.801380	-1.189975
N	3.240923	6.215281	-0.053449
C	2.376598	6.219296	1.200998
H	1.714728	7.086634	1.075584
C	4.249820	5.113371	0.029722
H	4.853707	5.274399	0.930221
H	4.860530	5.124684	-0.878862
C	2.385995	6.053120	-1.280176
H	1.788342	5.141508	-1.184659
H	1.721347	6.924247	-1.339494
C	3.939460	7.569903	-0.097557
H	4.464280	7.654719	0.860402
H	3.053215	5.982174	-2.145712
H	3.135814	8.310653	-0.102886
H	3.069855	6.430250	2.028577
H	3.722473	4.159767	0.094657
C	1.601392	4.937364	1.441753
H	0.864778	4.727802	0.656989
H	2.261728	4.067357	1.542386
H	1.092786	5.015071	2.412083
C	4.875118	7.774457	-1.269449
H	4.323530	7.864499	-2.213115
H	5.402577	8.717334	-1.084773
H	5.613112	6.975726	-1.391059
C	4.476390	0.149735	0.806077
H	3.755658	0.334384	0.006402
H	3.947645	-0.248096	1.674652
C	6.323519	-0.325510	-0.776929
H	6.853497	0.532815	-0.347315
H	7.051358	-1.116625	-0.996472
N	5.467806	-0.880555	0.356890
C	6.421377	-1.203048	1.501499
H	7.237137	-1.786524	1.055177
H	6.815778	-0.228970	1.813632
H	5.517616	-2.871536	-0.360779
H	5.023462	1.071035	1.032110
C	5.793079	-1.946127	2.662802
H	5.489381	-2.964969	2.393515
H	6.547244	-2.007907	3.453511
H	4.914532	-1.442386	3.082029
C	5.545416	0.086808	-2.007748
H	6.264333	0.483009	-2.738757
H	5.026493	-0.752788	-2.487005
H	4.831254	0.888006	-1.795172

$[N_{1133}]_2[VBH]$

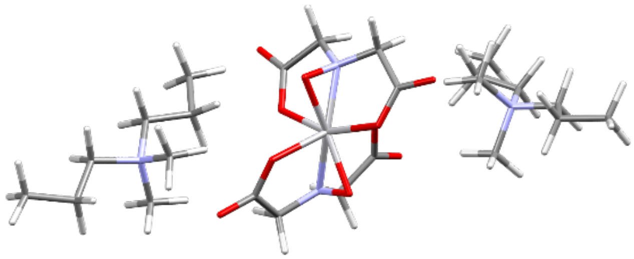

87

N -6.177971 -0.964721 0.087767
C -4.768781 -1.429327 -0.128985
H -4.647035 -1.767908 -1.162902
H -4.078149 -0.607206 0.091163
C -6.341007 -0.327195 1.469301
H -6.091102 -1.111234 2.195755
C -6.561043 0.026516 -0.964035
H -6.424254 -0.423024 -1.951318
H -5.904193 0.895173 -0.873085
C -7.135813 -2.146760 0.059612
H -6.768773 -2.845490 0.822172
H -8.106912 -1.734017 0.362532
H -7.414380 -0.113475 1.548376
H -7.608678 0.309117 -0.801925
H -4.581950 -2.262006 0.553893
C -7.269767 -2.863587 -1.270545
H -6.324297 -3.347197 -1.549334
H -7.570658 -2.160054 -2.058240
C -5.518748 0.922031 1.726841
H -4.445919 0.697920 1.784240
H -5.672179 1.674151 0.941289
C -5.959930 1.521578 3.062863
H -7.015026 1.837915 3.022646
H -5.865893 0.778184 3.864977
H -5.349065 2.395951 3.314105
C -8.370901 -3.908838 -1.101850
H -8.496305 -4.514974 -2.008604
H -8.127871 -4.585321 -0.272572
H -9.326894 -3.417760 -0.882786
V 0.000000 0.000000 0.000000
O 2.057210 -0.248526 -0.219490
O 4.053686 -0.152500 0.833051
O 0.261486 -1.400645 1.396073
O -2.667577 0.565001 3.117718
O -1.804447 0.272359 1.048462
O 0.362309 2.026195 0.458939
O -0.284691 4.142745 0.020021
O 0.249005 0.660161 -1.838806
O -2.543588 -2.153337 -2.462016
O -0.942141 -1.589908 -0.963953

$[N_{1133}][VBH]$

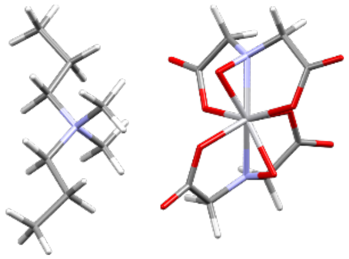

58

O -1.593834 1.183434 0.142418
O -3.213097 1.969320 1.490697
O 2.503750 -2.133130 2.410177
O 1.414860 -1.309857 0.626136
O 0.762441 1.132368 1.380483
N -0.101386 0.265084 2.002166
C -2.129147 1.413290 1.323378
C -1.329593 0.882895 2.506223
H -1.915765 0.111485 3.022421
H -1.089120 1.695329 3.203982
C 0.547880 -0.792277 2.784931
H 0.965500 -0.383919 3.713425
H -0.221693 -1.542664 3.017434
C 1.589968 -1.472676 1.910948
V 0.000000 0.000000 0.000000
O 1.454487 0.924483 -0.980570
O 2.682287 1.037017 -2.861099
O -2.393936 -3.228404 -0.397787
O -1.261326 -1.495077 0.472127
O -0.786485 -0.197686 -1.790803
N 0.175491 -1.171920 -1.635893
C 1.897290 0.450729 -2.120595
C 1.372602 -0.954150 -2.440881
H 2.120223 -1.705053 -2.147788
H 1.142942 -1.064858 -3.509476
C -0.340573 -2.533450 -1.456992
H -0.716212 -2.939874 -2.401972
H 0.476333 -3.160123 -1.065943
C -1.435950 -2.455274 -0.400564
C 4.773868 -2.174808 -0.276416
H 4.215529 -1.892686 -1.172672
H 4.075882 -2.496126 0.502622
C 4.562964 0.096831 0.633191
H 3.921540 0.363488 -0.209880
H 3.953804 -0.305113 1.446209
C 6.453149 -0.458062 -0.887176
H 7.052151 0.334664 -0.422003
H 7.112228 -1.304631 -1.117244
N 5.523588 -0.970845 0.205843
C 6.416217 -1.350986 1.381742

N	0.625786	-0.168569	1.917337
N	-1.050383	0.951125	-1.451748
C	2.801977	-0.167015	0.819146
C	2.059669	-0.047351	2.157606
H	2.401079	-0.825599	2.854990
H	2.260374	0.937776	2.599469
C	-0.301095	0.350501	2.915880
H	0.007669	1.373605	3.170405
H	-0.287561	-0.262263	3.826570
C	-1.719896	0.399342	2.335864
C	-0.333892	2.913957	-0.170255
C	-1.304814	2.372611	-1.228611
H	-2.328767	2.474598	-0.843891
H	-1.211851	2.941223	-2.165400
C	-2.047937	0.162736	-2.166716
H	-2.003522	0.363160	-3.248245
H	-3.042161	0.444664	-1.790543
C	-1.833427	-1.326060	-1.868151
N	3.751753	4.397850	0.854294
C	3.216205	5.550183	1.700710
H	3.236995	6.427744	1.041475
C	3.528508	3.080833	1.520566
H	4.005523	3.098678	2.505587
H	3.960897	2.290283	0.902582
C	3.047042	4.382739	-0.474284
H	1.975612	4.269653	-0.281977
H	3.254522	5.326102	-0.989507
C	5.233527	4.663214	0.661592
H	5.652580	4.804316	1.667398
H	3.421470	3.542288	-1.065298
H	5.288651	5.623042	0.132539
H	3.970607	5.731235	2.476875
H	2.448562	2.917642	1.595818
C	1.854572	5.329399	2.332110
H	1.104194	5.071001	1.570302
H	1.890693	4.477780	3.027972
C	6.003906	3.580035	-0.071172
H	5.551048	3.381202	-1.053768
H	5.992303	2.643203	0.504514
C	7.444680	4.063646	-0.229297
H	7.907285	4.276131	0.745774
H	8.068137	3.316370	-0.738729
H	7.470816	4.985381	-0.826201
C	1.457781	6.582214	3.116597
H	0.445395	6.489617	3.525023
H	2.120394	6.737616	3.979186
H	1.519353	7.490101	2.502886
H	7.246621	-1.928536	0.957154
H	6.803701	-0.395505	1.760524
H	5.491631	-2.964902	-0.513388
H	5.141323	0.972076	0.942961
C	5.728335	-2.146603	2.475753
H	5.412208	-3.124665	2.093043
H	4.819349	-1.645457	2.828660
C	6.688228	-2.353257	3.646139
H	6.204496	-2.980636	4.406948
H	7.611232	-2.858025	3.320409
H	6.956907	-1.398364	4.123403
C	5.777900	0.065330	-2.144419
H	5.091378	-0.680223	-2.569240
H	5.197520	0.968246	-1.918329
C	6.857608	0.374259	-3.186071
H	7.612542	1.064778	-2.785908
H	7.383200	-0.541771	-3.484751
H	6.412320	0.823247	-4.084154

$[N_{1144}]_2[VBH]$

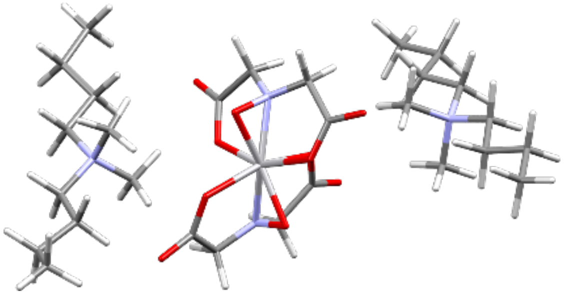

99

N -5.229349 -1.596082 1.245593
C -3.947491 -2.280848 1.605336
H -3.204531 -2.068214 0.832754
H -3.590664 -1.865435 2.549194
C -6.188020 -1.666082 2.424226
H -6.226627 -2.730108 2.697677
C -4.957253 -0.169430 0.875760
H -4.362520 -0.179682 -0.040733
H -4.361556 0.281549 1.676230
C -5.907858 -2.334436 0.100681
H -6.224058 -3.293890 0.532122
H -6.787291 -1.730405 -0.158730
H -7.159591 -1.352953 2.021768
H -5.920302 0.326565 0.721420
H -4.156425 -3.352870 1.663618
C -5.023123 -2.623717 -1.095087
H -4.119585 -3.154666 -0.773354
H -4.701387 -1.710056 -1.613295
C -5.819304 -0.814928 3.623180
H -4.771555 -0.958304 3.921440
H -5.931893 0.252270 3.381883
C -6.738872 -1.177574 4.788886
H -7.790456 -1.011290 4.505214
H -6.654907 -2.250008 5.018600
C -5.770439 -3.539652 -2.069404
H -6.334527 -4.283235 -1.485722
H -6.517614 -2.965715 -2.639023
C -6.412403 -0.375375 6.039425
H -7.074451 -0.671856 6.862430
H -5.374746 -0.570793 6.349400
H -6.514346 0.705991 5.856591
C -4.802674 -4.254162 -3.007562
H -4.111111 -4.887637 -2.434997
H -5.339446 -4.894096 -3.720352
H -4.182803 -3.534031 -3.556951
V 0.000000 0.000000 0.000000
O 2.031859 -0.038541 -0.404434
O 4.093339 0.049000 0.506027
O 0.446872 -1.587450 1.116352

$[N_{1144}][VBH]$

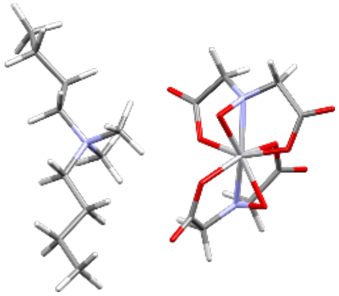

64

O -1.587590 1.150523 0.332476
O -3.035332 1.985103 1.844103
O 2.505144 -2.437905 2.117280
O 1.402076 -1.399287 0.456783
O 0.872651 0.992220 1.410169
N 0.015067 0.110462 2.023139
C -2.011157 1.368069 1.559209
C -1.146635 0.742668 2.648459
H -1.719271 -0.030783 3.177539
H -0.820328 1.525457 3.347107
C 0.662863 -1.018100 2.697214
H 1.171919 -0.704234 3.617519
H -0.119260 -1.748326 2.950788
C 1.615187 -1.684520 1.716116
V 0.000000 0.000000 0.000000
O 1.391677 1.010781 -0.981978
O 2.331125 1.407009 -2.988279
O -2.363425 -3.263194 -0.500026
O -1.226914 -1.549095 0.399635
O -0.898691 -0.035562 -1.749892
N 0.070303 -1.016752 -1.752719
C 1.697011 0.690599 -2.221433
C 1.212085 -0.708921 -2.615040
H 2.002342 -1.444887 -2.404123
H 0.934712 -0.757692 -3.677806
C -0.449749 -2.387124 -1.687801
H -0.927323 -2.670225 -2.634448
H 0.390191 -3.061678 -1.461156
C -1.443688 -2.445393 -0.527560
C 4.800958 -2.243858 -0.295635
H 4.144574 -2.021140 -1.140202
H 4.211182 -2.692052 0.508356
C 4.320585 -0.037307 0.695029
H 3.503104 0.016609 -0.027178
H 3.950605 -0.455888 1.634751
C 6.160926 -0.287197 -0.946461
H 6.694703 0.556657 -0.490914
H 6.899226 -1.031737 -1.270936
N 5.394486 -0.951382 0.184237

O	-2.462045	-0.009488	3.327020
O	-1.704211	0.005308	1.203890
O	0.257646	1.951692	0.763770
O	-0.559065	4.053998	0.694320
O	0.067931	0.963116	-1.704533
O	-2.312353	-1.924134	-2.851487
O	-0.879407	-1.485793	-1.156339
N	0.782516	-0.443960	1.829633
N	-1.221137	1.070525	-1.209508
C	2.852953	-0.082452	0.578828
C	2.225686	-0.269777	1.970374
H	2.675659	-1.130508	2.491328
H	2.414108	0.630942	2.566237
C	-0.093762	-0.180726	2.971840
H	0.205844	0.775762	3.421984
H	-0.011067	-0.973467	3.732905
C	-1.547991	-0.051945	2.485943
C	-0.541293	2.864168	0.324313
C	-1.557797	2.412069	-0.736024
H	-2.554520	2.376286	-0.275807
H	-1.573352	3.120504	-1.574396
C	-2.172644	0.328837	-2.027187
H	-2.200766	0.735090	-3.050542
H	-3.173950	0.430495	-1.587008
C	-1.761980	-1.158186	-2.041757
N	3.592913	4.438590	1.648475
C	3.392803	5.194401	2.956988
H	3.554533	6.247112	2.689943
C	3.241464	2.996404	1.822804
H	3.850186	2.591378	2.638848
H	3.448243	2.463318	0.892861
C	2.720662	5.036126	0.581732
H	1.674055	4.882728	0.862343
H	2.975736	6.094316	0.468506
C	5.064203	4.580309	1.269747
H	5.622745	4.156177	2.116218
H	2.904768	4.505100	-0.351280
H	5.245992	5.662245	1.221033
H	4.185375	4.855122	3.640796
H	2.171519	2.919002	2.038271
C	2.034454	4.986030	3.612452
H	1.224334	4.999088	2.871566
H	2.001034	3.999354	4.093005
C	5.459554	3.907666	-0.036210
H	4.887335	4.330128	-0.872948
H	5.248571	2.827828	-0.005402
C	6.948541	4.105834	-0.340029
H	7.555545	3.728895	0.500708
H	7.161683	5.183259	-0.426156
C	1.773011	6.066634	4.666972
H	2.551913	5.999808	5.442657
H	1.874721	7.066014	4.214845
C	7.331012	3.391227	-1.636851
C	6.404823	-1.185387	1.306702
H	7.383660	-1.282367	0.830377
H	6.405957	-0.258929	1.896379
H	5.613619	-2.902026	-0.614411
H	4.765777	0.953281	0.840450
C	6.178407	-2.409937	2.169574
H	6.256678	-3.309849	1.545304
H	5.183117	-2.418371	2.636919
C	7.279349	-2.459259	3.234802
H	8.241643	-2.204409	2.761296
H	7.078092	-1.703395	4.010877
C	5.323876	0.210625	-2.107262
H	4.717843	-0.591407	-2.553705
H	4.647856	1.004649	-1.771798
C	6.271928	0.778529	-3.170187
H	7.089159	1.313498	-2.663066
H	6.751762	-0.049431	-3.709776
C	5.564381	1.710066	-4.147151
H	6.276980	2.158280	-4.854414
H	4.804391	1.172501	-4.729787
H	5.060721	2.521589	-3.602913
C	7.397845	-3.845060	3.859446
H	7.836364	-4.548329	3.139674
H	8.033510	-3.822268	4.752164
H	6.417169	-4.238948	4.161028

H 8.383858 3.563874 -1.894420
H 7.178847 2.304608 -1.542094
H 6.723405 3.731982 -2.486732
C 0.392974 5.931679 5.306264
H -0.399110 6.022854 4.548684
H 0.267415 4.956115 5.795373
H 0.214906 6.698491 6.071138

Asymmetric disubstituted [N11xy]

$[N_{1123}]_2[VBH]$

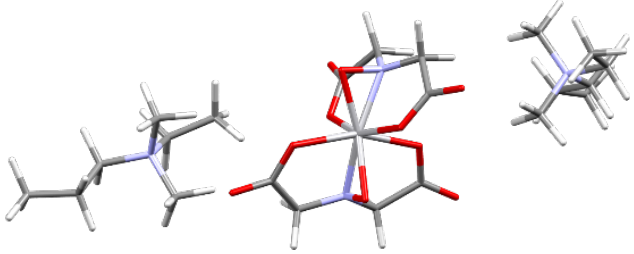

81

N -6.241376 -1.351040 0.650974
C -4.901176 -1.938449 0.970133
H -4.411063 -2.235409 0.036853
H -4.287309 -1.192459 1.487091
C -6.917675 -0.853495 1.924477
H -6.974033 -1.715713 2.600969
C -6.103792 -0.239546 -0.343651
H -5.643469 -0.643498 -1.249133
H -5.454675 0.533866 0.074512
C -7.165230 -2.425314 0.088408
H -7.237352 -3.189623 0.869051
H -8.132621 -1.924404 -0.040345
H -7.929170 -0.568591 1.610356
H -7.093930 0.169409 -0.564292
H -5.065679 -2.815536 1.604149
C -6.696893 -3.053197 -1.207983
H -5.708180 -3.515009 -1.114955
H -7.406297 -3.857276 -1.442422
H -6.711123 -2.342889 -2.039688
C -6.247291 0.320988 2.614222
H -5.260263 0.036465 3.005311
H -6.096944 1.153597 1.913077
C -7.153567 0.795262 3.753471
H -8.182660 0.932466 3.391478
H -7.198766 0.054190 4.559555
H -6.800309 1.757537 4.141016
V 0.000000 0.000000 0.000000
O 1.944670 0.197131 -0.662127
O 4.101783 0.336851 -0.015292
O 0.811380 -1.451561 1.103947

$[N_{1123}][VBH]$

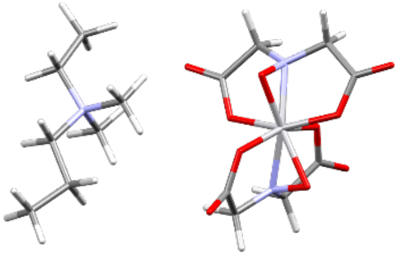

55

O -1.635080 1.109847 0.234020
O -3.195094 1.863091 1.669669
O 2.708888 -2.036330 2.243894
O 1.463310 -1.286933 0.532632
O 0.724014 1.076881 1.441720
N -0.085105 0.126969 2.018362
C -2.138652 1.276136 1.436594
C -1.341833 0.636654 2.562673
H -1.888385 -0.212684 2.994299
H -1.147429 1.379912 3.344188
C 0.641485 -0.918642 2.741033
H 1.077293 -0.518574 3.667150
H -0.067692 -1.719743 2.992564
C 1.706892 -1.465158 1.805438
V 0.000000 0.000000 0.000000
O 1.380847 1.064235 -0.943567
O 2.344650 1.481390 -2.931033
O -2.275979 -3.301876 -0.586027
O -1.219996 -1.562404 0.364455
O -0.802086 -0.097246 -1.789153
N 0.195868 -1.044470 -1.725305
C 1.747494 0.734145 -2.159348
C 1.367880 -0.705247 -2.534225
H 2.182190 -1.391270 -2.259356
H 1.153813 -0.791923 -3.608688
C -0.291851 -2.427593 -1.653131
H -0.694751 -2.757596 -2.620918
H 0.546154 -3.073417 -1.347191
C -1.363450 -2.473409 -0.559736
C 5.425590 -2.365575 0.285513

O -2.037896 -0.632281 3.536634
O -1.570488 -0.268222 1.363509
O 0.130670 1.958753 0.691615
O -0.925881 3.950565 0.767221
O -0.354304 0.876623 -1.742838
O -2.765019 -2.406341 -1.891538
O -0.844184 -1.657846 -0.965050
N 1.023335 -0.235313 1.730383
N -1.527274 0.865399 -1.007005
C 2.883895 0.237732 0.219761
C 2.422362 0.185246 1.691688
H 3.066123 -0.493691 2.273351
H 2.480574 1.197401 2.119121
C 0.217072 -0.052579 2.939206
H 0.305037 0.999083 3.249778
H 0.559256 -0.701769 3.753946
C -1.256196 -0.351108 2.610823
C -0.826268 2.774808 0.390683
C -1.927525 2.184936 -0.508418
H -2.840712 2.061397 0.090469
H -2.134612 2.873321 -1.339076
C -2.539750 -0.054864 -1.525283
H -2.824664 0.210921 -2.555911
H -3.425345 0.013445 -0.881048
C -2.017228 -1.499089 -1.470180
N 5.044666 4.258230 0.737281
C 4.125035 5.259284 1.424177
H 4.336229 6.229121 0.962803
C 5.073721 2.981663 1.514328
H 5.582050 3.171303 2.464329
H 5.589126 2.221611 0.923685
C 4.574151 3.996034 -0.660587
H 3.640463 3.432285 -0.600903
H 4.406285 4.964743 -1.147738
C 6.425756 4.889021 0.713362
H 6.560643 5.335151 1.703448
H 5.329803 3.397008 -1.180498
H 6.364517 5.699163 -0.022414
H 4.480829 5.286933 2.462349
H 4.044481 2.651609 1.670561
C 2.647487 4.939065 1.327952
H 2.261737 5.143941 0.324138
H 2.382334 3.908249 1.595134
H 2.126841 5.600030 2.032340
C 7.585250 3.951159 0.430663
H 7.413965 3.363840 -0.483168
H 7.701096 3.253071 1.272466
C 8.848055 4.807662 0.311915
H 8.985696 5.420057 1.214959
H 9.753313 4.198071 0.199611
H 8.776163 5.492058 -0.546673
H 4.974201 -2.299548 -0.705151
H 4.654392 -2.636547 1.009895
C 4.902207 -0.015290 0.762691
H 4.359383 0.015809 -0.185964
H 4.218207 -0.333815 1.552640
C 7.014300 -0.589882 -0.391989
H 7.429627 0.353933 -0.022001
H 7.794292 -1.360396 -0.376188
N 5.998626 -1.030105 0.649269
C 6.761161 -1.127727 1.965434
H 7.651130 -1.731390 1.738713
H 7.088570 -0.102311 2.181289
H 6.241290 -3.094725 0.263051
H 5.358500 0.962186 0.952838
C 5.973168 -1.727798 3.112770
H 5.699986 -2.773065 2.930405
H 6.600093 -1.707139 4.014047
H 5.047326 -1.179609 3.322210
C 6.487997 -0.391985 -1.800518
H 5.979725 -1.296698 -2.163792
H 5.782412 0.448783 -1.828628
C 7.691089 -0.082984 -2.693921
H 8.294012 0.723106 -2.258082
H 8.353744 -0.955929 -2.772749
H 7.381004 0.232178 -3.695412

$[N_{1124}]_2[VBH]$

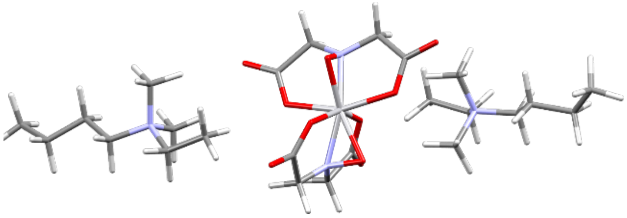

87

N -5.898149 -2.606243 2.062615
C -5.001977 -3.497096 2.862808
H -4.007823 -3.045704 2.848665
H -5.371411 -3.519254 3.891716
C -7.341539 -3.057005 2.170817
H -7.373153 -4.053669 1.708343
C -5.736532 -1.192448 2.553289
H -4.673389 -0.933506 2.478467
H -6.042785 -1.154275 3.603991
C -5.547531 -2.683997 0.574123
H -5.905800 -3.665059 0.238899
H -6.145499 -1.889584 0.106760
H -7.911776 -2.364094 1.535868
H -6.366072 -0.536216 1.935427
H -4.990464 -4.496229 2.412401
C -4.072105 -2.558603 0.267015
H -3.532790 -3.446208 0.616283
H -3.957262 -2.528728 -0.825304
H -3.602911 -1.662267 0.690960
C -7.936525 -3.107506 3.567605
H -7.442278 -3.877008 4.177565
H -7.845351 -2.141770 4.083917
C -9.424555 -3.441852 3.432347
H -9.862750 -2.728022 2.719029
H -9.523805 -4.442259 2.978340
C -10.19617 -3.396986 4.746029
H -11.27033 -3.553012 4.564873
H -9.860088 -4.188638 5.427218
H -10.07406 -2.419910 5.230624
V 0.000000 0.000000 0.000000
O 1.987809 0.455778 -0.366103
O 4.077122 0.434846 0.489042
O 0.819888 -1.476932 1.026940
O -2.325594 -1.209611 3.198261
O -1.647709 -0.505147 1.171306
O -0.254706 1.939130 0.786043
O -1.562628 3.778440 0.713753
O -0.222020 0.950626 -1.711158
O -2.027394 -2.551538 -2.564701
O -0.484804 -1.664094 -1.180102
N 0.855981 -0.333578 1.818193
N -1.482396 0.721911 -1.175983

$[N_{1124}][VBH]$

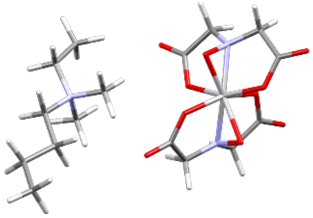

58

O -1.597249 1.174935 0.095794
O -3.209779 2.013518 1.419081
O 2.558590 -1.984859 2.473257
O 1.420903 -1.273919 0.672245
O 0.762306 1.206853 1.314847
N -0.083240 0.361474 1.990390
C -2.125149 1.455224 1.270516
C -1.316467 0.987760 2.470130
H -1.887651 0.236995 3.028685
H -1.087994 1.834787 3.128459
C 0.604453 -0.634753 2.819941
H 1.062287 -0.160346 3.694033
H -0.146934 -1.362026 3.156014
C 1.625063 -1.363496 1.959390
V 0.000000 0.000000 0.000000
O 1.463846 0.871121 -1.009692
O 2.869815 0.782890 -2.756040
O -2.332178 -3.322434 -0.160157
O -1.254040 -1.484060 0.547816
O -0.776718 -0.262995 -1.785634
N 0.166793 -1.245529 -1.586167
C 1.973712 0.298381 -2.067516
C 1.390611 -1.086964 -2.368910
H 2.100814 -1.859448 -2.041512
H 1.186612 -1.211053 -3.443086
C -0.390914 -2.585442 -1.382078
H -0.837297 -2.968665 -2.310062
H 0.420712 -3.248327 -1.045907
C -1.423257 -2.498059 -0.261921
C 5.760354 -2.426660 -0.558596
H 5.365007 -2.353379 -1.575152
H 5.033504 -2.928254 0.082861
C 4.686537 -0.317292 0.065411
H 4.277594 -0.218120 -0.943094
H 4.006902 -0.901628 0.692465
C 6.963772 -0.289175 -0.928602
H 7.212409 0.630791 -0.386871
H 7.863089 -0.914731 -0.970901
N 5.993212 -1.042922 -0.031398
C 6.660858 -1.113515 1.338259
H 7.655366 -1.540367 1.151416
H 6.774896 -0.066870 1.649215

C 2.837150 0.341405 0.596672<br>C 2.227568 0.128536 1.994474<br>H 2.804903 -0.582439 2.599148<br>H 2.212890 1.077543 2.547354<br>C -0.060044 -0.409632 2.959987<br>H -0.089978 0.578962 3.440524<br>H 0.285937 -1.148681 3.695953<br>C -1.464355 -0.748377 2.428809<br>C -1.265237 2.620745 0.356911<br>C -2.154200 1.922720 -0.680099<br>H -3.087300 1.610747 -0.189821<br>H -2.392748 2.609648 -1.502352<br>C -2.264893 -0.254022 -1.928654<br>H -2.420868 0.088680 -2.964572<br>H -3.242460 -0.371951 -1.441282<br>C -1.542527 -1.606456 -1.912574<br>N 2.805648 4.688217 0.570760<br>C 2.158877 5.670998 1.542146<br>H 1.743236 6.472967 0.921271<br>C 3.187856 3.432394 1.282140<br>H 3.816627 3.686073 2.143433<br>H 3.722071 2.771323 0.597099<br>C 1.827078 4.351260 -0.515450<br>H 0.914810 3.968133 -0.049689<br>H 1.628674 5.261177 -1.094219<br>C 4.023192 5.381325 -0.026614<br>H 4.553185 5.840627 0.814022<br>H 2.259119 3.565891 -1.142992<br>H 3.623796 6.198850 -0.640830<br>H 2.980267 6.106988 2.113455<br>H 2.264237 2.943118 1.600904<br>C 1.139454 5.061807 2.479306<br>H 0.292896 4.585794 1.965290<br>H 1.597769 4.334374 3.162631<br>H 0.773827 5.891643 3.094918<br>C 4.978368 4.485120 -0.791566<br>H 4.454889 3.881759 -1.549797<br>H 5.458829 3.770273 -0.106593<br>C 6.067058 5.341967 -1.454174<br>H 6.326199 6.181499 -0.788705<br>H 5.685263 5.804451 -2.375055<br>C 7.321045 4.527675 -1.753875<br>H 8.028362 5.113298 -2.353484<br>H 7.815296 4.207889 -0.824168<br>H 7.069135 3.619973 -2.324162	H 6.711945 -2.966703 -0.559790<br>H 4.870127 0.679408 0.476840<br>C 5.905509 -1.925239 2.372468<br>H 5.855656 -2.988699 2.112025<br>H 6.455515 -1.835387 3.320152<br>H 4.885743 -1.563525 2.543673<br>C 6.472314 0.055692 -2.326033<br>H 6.010215 -0.806470 -2.830241<br>H 5.716711 0.853847 -2.269249<br>C 7.674048 0.521073 -3.162253<br>H 8.289944 1.209722 -2.565793<br>H 8.323010 -0.343025 -3.361615<br>C 7.259946 1.182794 -4.472738<br>H 8.131694 1.545739 -5.036516<br>H 6.713487 0.477600 -5.112285<br>H 6.596407 2.041387 -4.290541

$[N_{1134}]_2[VBH]$	$[N_{1134}][VBH]$
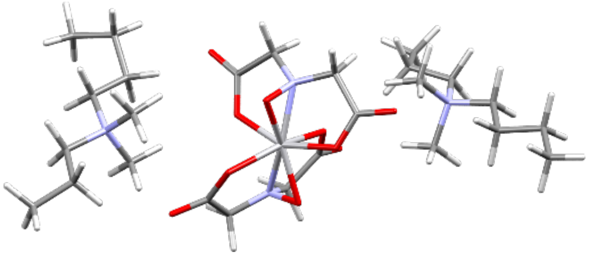	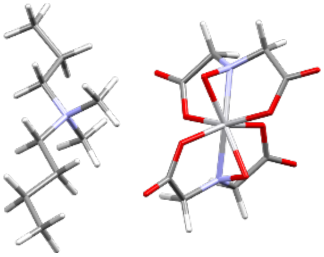
93	61
N -5.657595 -1.364022 1.009842	O -1.573206 1.200518 0.189090
C -4.314474 -2.010797 1.163547	O -3.129893 2.040647 1.577897
H -3.852481 -2.086910 0.173576	O 2.509931 -2.194016 2.351553
H -3.699261 -1.383630 1.815001	O 1.401264 -1.344414 0.590074
C -6.296180 -1.128881 2.374307	O 0.811202 1.094516 1.386003
H -6.206993 -2.088350 2.902968	N -0.064409 0.237371 2.006258
C -5.500674 -0.064525 0.285220	C -2.072243 1.444796 1.382573
H -5.080091 -0.275093 -0.703303	C -1.267070 0.875557 2.542718
H -4.803408 0.558700 0.852704	H -1.862656 0.109517 3.056871
C -6.598806 -2.275901 0.241673	H -0.993700 1.671239 3.247898
H -6.796559 -3.125787 0.909072	C 0.561956 -0.850615 2.764270
H -7.513953 -1.686487 0.098908	H 0.985871 -0.472524 3.703318
H -7.353681 -0.929386 2.161965	H -0.222291 -1.593408 2.973163
H -6.488165 0.400570 0.201807	C 1.590630 -1.526104 1.870146
H -4.477641 -3.005815 1.585274	V 0.000000 0.000000 0.000000
C -6.040993 -2.793062 -1.069631	O 1.437521 0.925564 -1.008450
H -5.291289 -3.564018 -0.864252	O 2.563782 1.074333 -2.954173
H -5.532822 -2.003617 -1.638760	O -2.411803 -3.239357 -0.357586
C -5.681035 0.004597 3.181574	O -1.272337 -1.492092 0.476527
H -4.583172 -0.012144 3.122771	O -0.834244 -0.165342 -1.771514
H -6.025381 0.971488 2.782892	N 0.120001 -1.152339 -1.656501
C -6.094368 -0.095347 4.653755	C 1.825445 0.472393 -2.179308
H -5.651932 -1.010229 5.081357	C 1.298673 -0.933584 -2.490168
H -5.658346 0.765412 5.180750	H 2.055049 -1.681965 -2.212319
C -7.175149 -3.385013 -1.897675	H 1.046558 -1.042472 -3.553912
H -6.772266 -3.848737 -2.806560	C -0.410358 -2.509610 -1.491118
H -7.711377 -4.158349 -1.334784	H -0.829865 -2.883482 -2.432525
H -7.913617 -2.625685 -2.191212	H 0.410878 -3.160234 -1.150942
C -7.607585 -0.108568 4.865835	C -1.465281 -2.451491 -0.391086
H -7.850092 -0.116277 5.935973	C 4.933116 -2.296832 -0.134181
H -8.056149 0.794338 4.427635	H 4.388132 -2.146426 -1.069576
H -8.076026 -1.003049 4.431895	H 4.242538 -2.642114 0.640822
V 0.000000 0.000000 0.000000	C 4.439923 -0.008316 0.633017
O 2.038276 -0.065402 -0.458181	H 3.742053 0.068311 -0.203283
O 4.105468 0.015401 0.452305	H 3.913932 -0.385395 1.510390
O 0.395824 -1.707917 0.914325	C 6.406003 -0.423148 -0.815484
O -2.365028 -0.378206 3.372674	H 6.929261 0.432953 -0.371291
O -1.653284 0.019710 1.273655	H 7.138642 -1.211587 -1.024317
O 0.473827 1.851474 0.904183	N 5.522115 -0.983931 0.287121
O -0.166294 4.010995 1.069167	C 6.430706 -1.184209 1.488837

O	0.028012	1.039884	-1.659254
O	-3.165324	-1.487914	-2.238475
O	-1.203069	-1.322705	-1.121835
N	0.816433	-0.676151	1.743745
N	-1.189093	1.275259	-1.041407
C	2.870740	-0.181451	0.508898
C	2.269385	-0.576542	1.867197
H	2.699103	-1.528333	2.219204
H	2.507547	0.204661	2.599941
C	-0.014189	-0.519116	2.935749
H	0.329489	0.366109	3.484441
H	0.062218	-1.397989	3.595041
C	-1.469814	-0.280035	2.515986
C	-0.251251	2.864926	0.581632
C	-1.318659	2.620863	-0.486202
H	-2.301644	2.700377	-0.004992
H	-1.238382	3.377007	-1.279676
C	-2.328446	0.696012	-1.746390
H	-2.358337	1.054215	-2.788724
H	-3.249910	1.011427	-1.239506
C	-2.232459	-0.836700	-1.714609
N	3.725346	4.211268	2.035688
C	3.285669	5.146915	3.158080
H	3.349000	6.159225	2.737723
C	3.451270	2.785880	2.411376
H	3.923732	2.602804	3.383193
H	3.877084	2.130526	1.644917
C	2.984403	4.553894	0.778224
H	1.910234	4.504774	0.982748
H	3.300541	5.552198	0.449595
C	5.219665	4.416981	1.834351
H	5.686204	4.064549	2.764650
H	3.233397	3.809467	0.018842
H	5.353698	5.502503	1.729626
H	4.048128	5.032223	3.941919
H	2.366744	2.638624	2.441057
C	1.899140	4.855583	3.718363
H	1.180367	4.646342	2.913146
H	1.947347	3.945011	4.326329
C	5.800644	3.721138	0.615500
H	5.329814	4.095649	-0.304222
H	5.630099	2.633969	0.650389
C	7.302826	4.001373	0.523228
H	7.809893	3.625925	1.427345
H	7.456637	5.092082	0.499002
C	1.400166	6.014210	4.583347
H	0.463665	5.740968	5.087225
H	2.129103	6.269501	5.363679
H	1.255016	6.923014	3.987443
C	7.896688	3.370202	-0.732607
H	8.961662	3.607592	-0.833550
H	7.802751	2.275397	-0.700543
H	7.385764	3.716690	-1.641846
H	7.326030	-1.689196	1.106945
H	6.704265	-0.169548	1.807605
H	5.749541	-3.007980	-0.289436
H	4.903707	0.965406	0.818847
C	5.844885	-1.975573	2.644051
H	5.629108	-3.008074	2.337736
H	4.897455	-1.545445	2.991671
C	6.867271	-1.978658	3.781434
H	6.472305	-2.529854	4.642658
H	7.804087	-2.462277	3.463790
H	7.092376	-0.957648	4.123840
C	5.690490	0.011477	-2.079939
H	5.087785	-0.803682	-2.508042
H	5.022636	0.857783	-1.873688
C	6.742762	0.435160	-3.108784
H	7.426313	1.161607	-2.645774
H	7.367302	-0.431982	-3.363957
C	6.111762	1.016374	-4.370212
H	6.871142	1.422045	-5.053676
H	5.554907	0.237730	-4.907924
H	5.404834	1.821152	-4.124448

Monosubstituted [N444x]

$[N_{4441}]_2[VBH]$

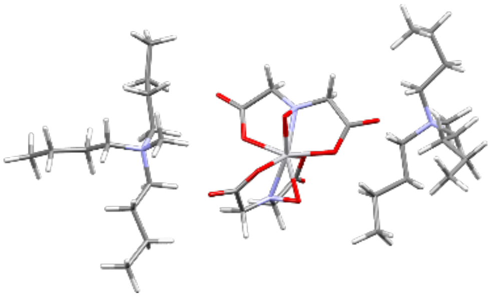

117

N -5.450963 -3.475415 2.023907
C -4.433504 -2.373018 2.332196
H -3.659550 -2.464674 1.561788
H -4.981781 -1.436029 2.165985
C -6.720883 -3.197034 2.773597
H -7.409919 -4.032533 2.618160
H -6.469648 -3.109052 3.835059
C -5.683512 -3.407185 0.520730
H -4.688685 -3.527795 0.073532
H -6.035223 -2.384814 0.329104
C -4.952349 -4.837774 2.450762
H -4.892633 -4.799267 3.544485
H -5.744791 -5.550217 2.187874
C -3.786154 -2.419822 3.703650
H -4.501678 -2.264375 4.524391
H -3.305744 -3.396720 3.870419
C -2.696811 -1.341122 3.756428
H -3.163539 -0.345865 3.740154
H -2.086608 -1.399151 2.841191
C -6.672760 -4.415244 -0.036914
H -6.297765 -5.445073 0.093168
H -7.643122 -4.324211 0.469200
C -6.892876 -4.141766 -1.532067
H -7.257872 -3.110279 -1.649228
H -7.696690 -4.804014 -1.883504
C -3.606920 -5.231420 1.875927
H -2.897015 -4.404299 1.991140
H -3.668935 -5.397102 0.792358
C -3.046019 -6.470081 2.582599
H -3.496235 -7.383601 2.168325
H -3.332157 -6.449321 3.646008
C -1.523337 -6.535650 2.482001
H -1.122107 -7.439867 2.959653
H -1.193262 -6.531691 1.433854
H -1.076414 -5.660146 2.975421
C -1.802915 -1.483552 4.983044
H -1.016924 -0.717321 4.975623

$[N_{4441}][VBH]$

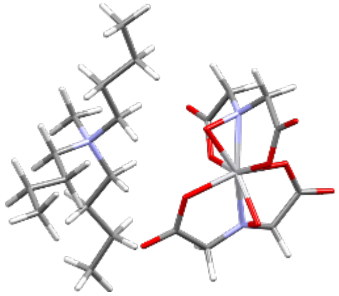

73

O -1.419679 1.322958 -0.371012
O -2.506478 3.169658 0.311169
O 2.494404 -1.076795 3.078966
O 1.321799 -1.053268 1.158671
O 0.978981 1.534919 0.679763
N 0.095733 1.153990 1.666253
C -1.711147 2.254748 0.508282
C -1.009884 2.087939 1.854629
H -1.714916 1.660542 2.582551
H -0.643461 3.050972 2.229163
C 0.737547 0.548140 2.834615
H 1.335559 1.289136 3.385554
H -0.053499 0.158560 3.491391
C 1.604100 -0.617076 2.354402
V 0.000000 0.000000 0.000000
O 1.535898 0.137092 -1.265353
O 2.874444 -0.898351 -2.737873
O -2.643062 -2.792705 1.318304
O -1.331344 -0.983132 1.131178
O -0.934708 -0.788333 -1.510542
N -0.125736 -1.775035 -0.985103
C 1.885450 -0.887171 -2.001166
C 1.015800 -2.127128 -1.825764
H 1.597135 -2.899355 -1.302900
H 0.663640 -2.537766 -2.782254
C -0.871543 -2.837656 -0.311926
H -1.485853 -3.391006 -1.034656
H -0.164043 -3.543820 0.140142
C -1.706589 -2.200280 0.790112
C 4.613549 -1.045026 0.051228
H 3.887127 -0.667744 -0.669283
H 4.065558 -1.477484 0.900021
C 4.219562 1.141724 1.059764
H 3.469829 1.165644 0.261122
H 3.763790 0.641732 1.921496
C 6.211314 0.832374 -0.424692
H 6.677328 1.686783 0.076022

H -2.364635 -1.385990 5.921645	H 7.005107 0.110191 -0.650403
H -1.303437 -2.467223 4.985786	N 5.315082 0.182361 0.603940
C -5.640573 -4.346894 -2.382346	C 6.150794 -0.219795 1.781045
H -5.848982 -4.158905 -3.445196	H 6.965217 -0.870651 1.451510
H -4.828730 -3.667778 -2.090288	H 6.540684 0.677783 2.266210
H -5.259650 -5.375863 -2.289414	C 5.511957 -2.069606 -0.618899
H -7.160403 -2.272594 2.395251	H 5.907180 -1.652905 -1.559394
V 0.000000 0.000000 0.000000	H 6.370506 -2.337706 0.012596
O 2.031939 0.084582 -0.488573	C 4.661896 2.548928 1.429470
O 3.701523 1.605845 -0.468152	H 4.936132 3.119687 0.530926
O 0.760807 -0.262257 1.817822	H 5.543117 2.538316 2.083534
O -2.542714 1.852561 2.688400	C 5.495819 1.288691 -1.680560
O -1.703123 0.558033 1.038579	H 5.009868 0.447318 -2.197571
O -0.085250 1.889238 -0.940774	H 4.692515 1.995198 -1.425618
O -1.088864 3.089351 -2.567319	C 3.505328 3.251602 2.155517
O -0.039387 -0.695877 -1.847262	H 2.627469 3.273165 1.490356
O -2.177615 -3.519482 -0.104343	H 3.214021 2.646792 3.029619
O -0.663012 -1.935764 0.406329	C 3.855456 4.661551 2.623093
N 0.784337 1.095306 1.537385	H 4.152814 5.298559 1.780860
N -1.335690 -0.327708 -1.501073	H 2.999015 5.124401 3.134473
C 2.685791 1.081525 0.015856	H 4.702453 4.640086 3.322856
C 2.121116 1.653228 1.317383	C 4.703735 -3.334873 -0.927562
H 2.810931 1.437222 2.149624	H 4.391148 -3.794490 0.022939
H 2.024215 2.742181 1.226276	H 3.789030 -3.052539 -1.470968
C -0.173610 1.840562 2.349632	C 5.492379 -4.340784 -1.762330
H -0.081502 2.903172 2.100975	H 5.672701 -3.958413 -2.778600
H 0.044662 1.702703 3.420139	H 6.466514 -4.559853 -1.299205
C -1.600402 1.385563 2.017167	H 4.946144 -5.287362 -1.874657
C -0.925246 2.036572 -1.913015	C 6.489850 1.982151 -2.615594
C -1.807015 0.839379 -2.238251	H 7.002283 2.793749 -2.077566
H -2.829028 1.062913 -1.890585	H 7.290803 1.270867 -2.881167
H -1.804056 0.639987 -3.321208	C 5.806634 2.528681 -3.865734
C -2.249988 -1.449507 -1.316623	H 5.197871 1.766264 -4.373453
H -2.419253 -1.985953 -2.263230	H 5.125162 3.349199 -3.605545
H -3.211246 -1.045402 -0.962628	H 6.544113 2.923399 -4.575620
C -1.664237 -2.392398 -0.265900	H 5.502391 -0.765619 2.476035
N 2.937817 4.717135 -2.483520	
C 3.321505 4.783254 -1.007033	
H 2.951932 3.845061 -0.575289	
H 2.764941 5.625970 -0.575141	
C 3.217281 3.335187 -3.013400	
H 4.293170 3.147705 -2.907982	
H 2.903754 3.296649 -4.066297	
C 1.453195 5.007261 -2.563425	
H 0.948182 4.246745 -1.951763	
H 1.317741 5.988617 -2.088681	
C 3.738715 5.700513 -3.309718	
H 4.789447 5.452572 -3.133397	
H 3.524611 5.468407 -4.359071	
C 4.805470 4.931320 -0.738252	
H 5.133708 5.954825 -0.987415	
H 5.389938 4.239422 -1.360793	
C 5.108119 4.645312 0.732027	

H 4.444423 5.248289 1.372894
H 4.889505 3.589907 0.946563
C 0.865057 5.005735 -3.962678
H 0.656122 3.977506 -4.277934
H 1.553084 5.429189 -4.707792
C -0.424658 5.826264 -3.962320
H -1.019309 5.534573 -3.084330
H -0.177947 6.894975 -3.849403
C 3.441100 7.153519 -2.989355
H 3.451946 7.313287 -1.899683
H 2.432373 7.417525 -3.339377
C 4.453833 8.106870 -3.625289
H 5.464709 7.845523 -3.273354
H 4.458209 7.964478 -4.718523
C 6.565463 4.962352 1.056477
H 6.802764 6.013428 0.826478
H 6.782468 4.797323 2.121580
H 7.233648 4.320830 0.462242
C -1.225043 5.578451 -5.233080
H -1.592576 4.546851 -5.225362
H -2.083050 6.256763 -5.328229
H -0.592577 5.698330 -6.124865
C 4.130522 9.557092 -3.276480
H 4.021386 9.690264 -2.190833
H 4.925078 10.237714 -3.604632
H 3.191364 9.872969 -3.752044
H 2.656016 2.616617 -2.409220

[N$_{444c}$]$_2$[VBH]

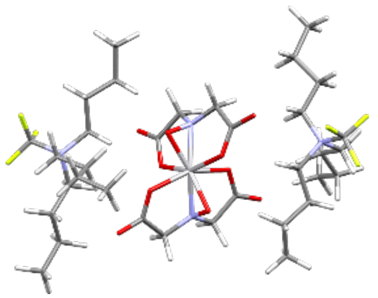

117

N -4.228962 -2.309354 2.720267
C -3.167403 -1.269556 3.132198
H -2.370656 -1.379469 2.386091
H -3.632149 -0.289004 2.972182
C -5.431981 -2.213722 3.646049
C -4.662944 -1.924070 1.280534
H -3.726910 -1.640360 0.788958
H -5.304111 -1.042441 1.395323
C -3.678069 -3.734276 2.776661
H -3.459167 -3.945927 3.828098
H -4.500386 -4.382634 2.450272

[N$_{444c}$][VBH]

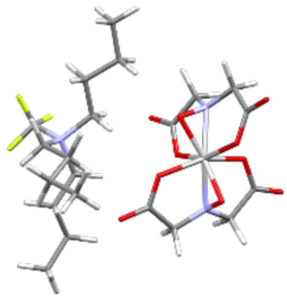

73

O -1.358726 1.325898 -0.612352
O -2.675205 3.070591 -0.092769
O 1.939921 -1.027190 3.439843
O 1.055046 -1.079572 1.372627
O 0.788832 1.523338 0.901875
N -0.273651 1.114953 1.679603
C -1.865243 2.196250 0.219499
C -1.404362 2.038494 1.668255
H -2.214929 1.599162 2.263894
H -1.124632 3.010695 2.099022
C 0.123620 0.470970 2.933746

C -2.610033 -1.399281 4.540238	H 0.540772 1.198760 3.644160
H -3.395985 -1.298345 5.299304	H -0.773339 0.004018 3.367666
H -2.133580 -2.381418 4.684034	C 1.136040 -0.624798 2.593305
C -1.571386 -0.294390 4.747642	V 0.000000 0.000000 0.000000
H -2.064553 0.677461 4.584296	O 1.783597 0.176000 -0.878706
H -0.804130 -0.395326 3.969943	O 3.393859 -0.796223 -2.096720
C -5.365092 -2.994144 0.461774	O -2.817796 -2.871619 0.620650
H -4.662852 -3.797600 0.198862	O -1.531218 -1.036216 0.780489
H -6.220657 -3.427651 0.992750	O -0.563934 -0.708265 -1.706190
C -5.871881 -2.338594 -0.827120	N 0.106469 -1.730349 -1.066125
H -5.006886 -1.939624 -1.371477	C 2.282265 -0.824120 -1.566781
H -6.544003 -1.509987 -0.560394	C 1.408015 -2.061713 -1.639780
C -2.452726 -3.929879 1.906659	H 1.843071 -2.874382 -1.040987
H -1.586651 -3.400482 2.328902	H 1.280960 -2.421203 -2.669538
H -2.605423 -3.521499 0.898647	C -0.775953 -2.811120 -0.633729
C -2.140718 -5.422196 1.783509	H -1.239576 -3.283334 -1.511170
H -3.020114 -5.939430 1.365371	H -0.162492 -3.559700 -0.114716
H -1.973381 -5.856513 2.784438	C -1.810028 -2.231714 0.315977
C -0.929826 -5.660976 0.883665	C 5.070806 -0.874883 0.641028
H -0.774697 -6.732241 0.687973	H 4.061925 -0.726519 0.250017
H -1.079070 -5.174804 -0.089042	H 5.000175 -1.538156 1.511488
H -0.019401 -5.237738 1.334654	C 4.172027 1.162124 1.739389
C -0.930443 -0.330618 6.128698	H 3.520936 1.201450 0.858070
H -0.180928 0.466103 6.222751	H 3.745305 0.425856 2.431587
H -1.673573 -0.185235 6.924256	C 6.137585 1.395708 0.141793
H -0.425006 -1.287477 6.324038	H 6.502667 2.288143 0.655832
C -6.614652 -3.341325 -1.700130	H 7.002737 0.825832 -0.212889
H -6.961685 -2.877894 -2.634021	N 5.471340 0.516283 1.208865
H -5.966809 -4.192289 -1.962992	C 5.974644 -1.445528 -0.434875
H -7.503740 -3.720184 -1.174426	H 5.871188 -0.850054 -1.351834
F -5.139662 -2.739053 4.851444	H 7.025029 -1.447633 -0.124006
F -5.786170 -0.937231 3.802965	C 4.320895 2.532370 2.368100
F -6.469637 -2.900285 3.147702	H 4.796480 3.233529 1.670661
V 0.000000 0.000000 0.000000	H 4.930161 2.482387 3.275489
O 2.025743 -0.516495 0.203521	C 5.191608 1.748249 -0.997871
O 3.924637 -0.062777 1.336515	H 4.442409 0.958722 -1.147384
O -0.036722 -0.509746 1.904889	H 4.646731 2.671496 -0.750703
O -2.803827 2.169904 2.156934	C 2.938991 3.082279 2.738866
O -1.773054 0.993970 0.521025	H 2.343915 3.214449 1.824208
O 0.733908 1.862266 -0.650644	H 2.406566 2.346260 3.360582
O 0.811020 3.296889 -2.392861	C 3.052296 4.398016 3.504284
O 0.434274 -0.533949 -1.837648	H 3.589425 5.144637 2.904426
O -2.758129 -2.806462 -1.277997	H 2.060092 4.793105 3.765316
O -1.194572 -1.695631 -0.089596	H 3.600358 4.244310 4.444368
N 0.470552 0.782561 1.817415	C 5.569471 -2.890815 -0.739482
N -0.784654 0.130209 -1.871464	H 5.669121 -3.498709 0.173510
C 2.688966 0.014433 1.164728	H 4.513379 -2.922523 -1.046583
C 1.885151 0.864504 2.155979	C 6.451316 -3.478337 -1.838967
H 2.064311 0.523997 3.188058	H 6.348258 -2.901012 -2.770909
H 2.206553 1.912640 2.070055	H 7.505048 -3.451042 -1.526381
C -0.433292 1.816879 2.316145	H 6.182468 -4.518296 -2.058940
H -0.012069 2.787706 2.025644	C 5.954249 1.915424 -2.317255
H -0.532797 1.776645 3.409608	H 6.735913 2.683548 -2.228424

C -1.799148 1.656726 1.631704<br>C 0.369520 2.288092 -1.821912<br>C -0.699813 1.435983 -2.526103<br>H -1.673819 1.936885 -2.432583<br>H -0.447150 1.323992 -3.591878<br>C -1.898955 -0.758683 -2.179541<br>H -1.789995 -1.195636 -3.183741<br>H -2.830609 -0.179549 -2.139175<br>C -1.964737 -1.864371 -1.116845<br>N 4.959895 2.799499 -0.916605<br>C 4.515709 2.934010 0.551652<br>H 4.520863 1.912595 0.955081<br>H 3.474153 3.264196 0.460889<br>C 6.367021 2.231238 -0.985264<br>C 3.975757 1.790557 -1.582684<br>H 4.316585 0.794379 -1.276438<br>H 3.011380 1.963984 -1.091260<br>C 4.933892 4.153436 -1.624861<br>H 5.728415 4.757587 -1.179014<br>H 5.196444 3.947311 -2.667938<br>C 5.287648 3.892509 1.439352<br>H 5.305346 4.905474 1.006051<br>H 6.321541 3.555729 1.592746<br>C 4.577763 3.930611 2.801211<br>H 3.511663 4.173418 2.654768<br>H 4.619616 2.924175 3.244230<br>C 3.790270 1.934613 -3.082685<br>H 4.737603 1.884950 -3.635816<br>H 3.318492 2.904067 -3.292343<br>C 2.840542 0.836108 -3.575448<br>H 3.398276 -0.103014 -3.693276<br>H 2.075446 0.628522 -2.816332<br>C 3.583831 4.846624 -1.522457<br>H 3.533356 5.427741 -0.590337<br>H 2.752960 4.125526 -1.502616<br>C 3.361969 5.771003 -2.722339<br>H 4.223554 6.445104 -2.838426<br>H 3.314566 5.148972 -3.632123<br>C 5.208901 4.944891 3.752015<br>H 5.069437 5.967015 3.375551<br>H 4.769394 4.886686 4.757613<br>H 6.288484 4.757843 3.849891<br>C 2.170813 1.243678 -4.884123<br>H 2.896240 1.399224 -5.698103<br>H 1.449536 0.485292 -5.210501<br>H 1.625002 2.184216 -4.727488<br>C 2.078251 6.579953 -2.570081<br>H 2.137970 7.240495 -1.694989<br>H 1.903509 7.215130 -3.448806<br>H 1.214279 5.914051 -2.447062<br>F 7.259442 3.122923 -0.544457<br>F 6.434888 1.109359 -0.273758<br>F 6.673120 1.948909 -2.276633	H 6.479738 0.970047 -2.534667<br>C 5.006503 2.252216 -3.466334<br>H 4.229273 1.482611 -3.563130<br>H 4.495690 3.209344 -3.286021<br>H 5.547914 2.333990 -4.417839<br>C 6.459017 0.292241 2.342548<br>F 6.956392 1.460742 2.783048<br>F 5.850241 -0.347959 3.348058<br>F 7.489061 -0.450009 1.912403

$[N_{444s}]_2[VBH]$

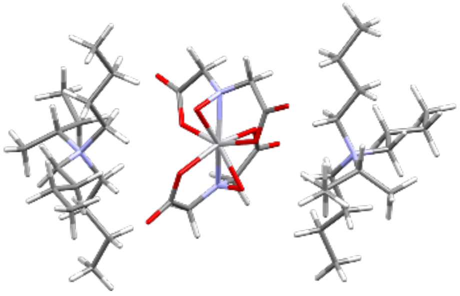

135

N -3.729788 -2.978013 2.600475
C -2.379534 -2.328418 2.940777
H -1.767527 -2.353397 2.032048
H -2.596960 -1.275357 3.144523
C -4.759960 -2.509740 3.662913
C -4.077977 -2.518732 1.192142
H -3.269682 -2.920732 0.577403
H -4.000998 -1.425146 1.185951
C -3.593290 -4.488194 2.626935
H -3.357166 -4.748202 3.665613
H -4.574716 -4.907685 2.392844
C -1.652621 -2.967699 4.112608
H -2.336075 -3.126967 4.958321
H -1.265006 -3.959560 3.831223
C -0.494063 -2.097988 4.607099
H -0.880185 -1.096608 4.850316
H 0.237898 -1.956470 3.797110
C -5.402291 -2.982090 0.608324
H -5.563124 -4.055277 0.796455
H -6.257343 -2.436473 1.032328
C -5.372360 -2.754429 -0.906915
H -4.486427 -3.254801 -1.328976
H -5.266338 -1.677467 -1.093231
C -2.547528 -5.057623 1.679226
H -1.669699 -4.402127 1.603668
H -2.946439 -5.123611 0.658746
C -2.093407 -6.440134 2.152306
H -2.964689 -7.105351 2.264260
H -1.637332 -6.342446 3.153895
C -1.098310 -7.058135 1.173314
H -0.679597 -7.994376 1.566856
H -1.589142 -7.296528 0.219073
H -0.266241 -6.363305 0.982632
C 0.151686 -2.724841 5.842319
H 0.984064 -2.121559 6.230449
H -0.576056 -2.828437 6.660302
H 0.540738 -3.729955 5.619286
C -6.651729 -3.242376 -1.568510
H -6.665164 -2.988853 -2.637512

$[N_{444s}][VBH]$

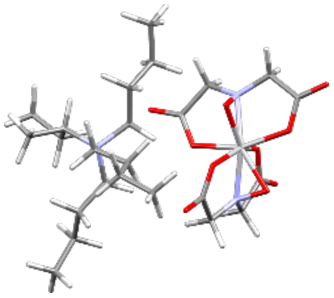

82

O -1.462285 1.238961 -0.580397
O -2.775656 2.993660 -0.073077
O 2.102177 -0.897805 3.390489
O 1.188683 -0.968699 1.340988
O 0.754863 1.597468 0.783899
N -0.231190 1.171345 1.647967
C -1.942603 2.141246 0.238070
C -1.399061 2.050038 1.668315
H -2.159271 1.604961 2.323913
H -1.135517 3.046017 2.051181
C 0.286247 0.608787 2.900356
H 0.760856 1.379972 3.522458
H -0.561094 0.168018 3.445377
C 1.286187 -0.498934 2.552867
V 0.000000 0.000000 0.000000
O 1.711471 0.238052 -1.002472
O 3.244315 -0.646335 -2.379373
O -2.662770 -2.956751 0.804476
O -1.417821 -1.088422 0.908404
O -0.625218 -0.810127 -1.631594
N 0.147554 -1.762798 -0.999984
C 2.208493 -0.740333 -1.714495
C 1.428034 -2.042960 -1.638980
H 1.958143 -2.775111 -1.014369
H 1.262218 -2.481181 -2.630877
C -0.633778 -2.878451 -0.471944
H -1.095416 -3.427207 -1.304296
H 0.046904 -3.550497 0.069967
C -1.672820 -2.303449 0.475969
C 5.363222 -1.334784 0.201328
H 4.645823 -1.078737 -0.582842
H 4.813950 -1.900153 0.959431
C 4.450715 0.701643 1.199289
H 3.834716 0.680439 0.292694
H 3.952382 0.070181 1.944987
C 6.571541 0.822214 -0.102126
H 6.902377 1.705805 0.456129
H 7.459284 0.232786 -0.354017
N 5.754547 -0.022308 0.863067

H	-6.756299	-4.336496	-1.479643
H	-7.532497	-2.775991	-1.109967
C	-5.197119	-1.064769	3.413228
H	-4.354000	-0.447060	3.071506
H	-5.956802	-1.063194	2.621011
C	-5.774143	-0.416622	4.673435
H	-6.665948	-0.942495	5.039599
H	-5.037133	-0.384414	5.488344
H	-6.069781	0.618038	4.455002
H	-4.195057	-2.550683	4.599660
C	-5.964298	-3.434901	3.797665
H	-5.697274	-4.427448	4.179584
H	-6.641233	-2.982681	4.528660
H	-6.512081	-3.539401	2.855464
V	0.000000	0.000000	0.000000
O	1.978198	0.035877	-0.615774
O	4.086764	0.580449	-0.039865
O	0.862081	-0.686241	1.660750
O	-2.209608	1.106163	3.339549
O	-1.606332	0.314329	1.309988
O	-0.166910	2.062183	-0.385932
O	-1.390426	3.632504	-1.453365
O	-0.198236	-0.076298	-1.968968
O	-1.943112	-3.487983	-1.026724
O	-0.535585	-1.989851	-0.101601
N	0.939113	0.702155	1.652670
N	-1.461929	0.044332	-1.404416
C	2.859926	0.548853	0.174330
C	2.286679	1.220792	1.427353
H	2.940535	1.075112	2.294456
H	2.200015	2.298367	1.229606
C	0.089419	1.320610	2.666693
H	0.162038	2.412587	2.564804
H	0.420203	1.025036	3.673554
C	-1.372531	0.888803	2.439989
C	-1.154087	2.453383	-1.125411
C	-2.087637	1.346954	-1.630267
H	-3.029970	1.368905	-1.062502
H	-2.296500	1.494987	-2.701361
C	-2.258225	-1.169651	-1.545103
H	-2.434055	-1.402045	-2.605716
H	-3.225334	-1.002395	-1.050313
C	-1.529369	-2.327894	-0.861627
N	2.541440	3.361634	-3.209525
C	2.711483	3.424152	-1.685534
H	2.718990	2.379145	-1.363462
H	1.799365	3.882616	-1.285567
C	3.562446	2.292835	-3.738695
H	4.397127	2.387395	-3.032431
C	1.090247	2.983203	-3.481496
H	0.916292	2.008664	-3.012407
H	0.481020	3.702860	-2.923380
C	2.831752	4.713902	-3.808969
C	6.517597	-0.250035	2.219535
H	6.270292	0.643015	2.799968
C	6.515046	-2.149926	-0.380134
H	6.662142	-1.864479	-1.434735
H	7.462720	-1.947155	0.131261
C	4.613641	2.128005	1.708811
H	4.745046	2.831987	0.877932
H	5.498300	2.235887	2.351260
C	5.823767	1.213208	-1.368843
H	5.256004	0.364467	-1.769197
H	5.081814	1.994582	-1.150992
C	5.957766	-1.452256	2.983307
H	4.861531	-1.385878	3.051594
H	6.197677	-2.385299	2.456382
C	6.548563	-1.525181	4.391602
H	7.584487	-1.879964	4.361431
H	6.539131	-0.535120	4.865598
C	3.390619	2.519647	2.539111
H	2.499823	2.501260	1.897284
H	3.219483	1.749796	3.305815
C	3.536209	3.877314	3.211139
H	3.632127	4.675813	2.466234
H	2.671944	4.082125	3.855733
H	4.435633	3.907008	3.840848
C	6.237366	-3.653048	-0.282720
H	6.314550	-3.954090	0.772717
H	5.204591	-3.869478	-0.589313
C	7.209407	-4.474802	-1.124045
H	7.104198	-4.229907	-2.193366
H	8.244594	-4.254974	-0.825474
H	7.022484	-5.550966	-1.011409
C	6.775084	1.701273	-2.466048
H	7.284833	2.626905	-2.161851
H	7.572516	0.951318	-2.608649
C	6.022094	1.921891	-3.777959
H	5.560109	0.985140	-4.125905
H	5.209880	2.651167	-3.641671
H	6.679708	2.308611	-4.566047
H	5.973693	-2.224815	5.011737
C	8.034251	-0.305904	2.108042
H	8.424213	-0.195850	3.129318
H	8.424489	-1.250826	1.717219
H	8.445788	0.517346	1.517271

H 3.880924 4.937387 -3.582787<br>H 2.745171 4.606555 -4.894056<br>C 3.951950 4.169237 -1.201962<br>H 3.847969 5.246906 -1.404106<br>H 4.868776 3.838926 -1.715409<br>C 4.156971 3.971377 0.304738<br>H 3.222530 4.214786 0.833364<br>H 4.377976 2.911902 0.498814<br>C 0.687658 2.974515 -4.946014<br>H 1.283614 2.260508 -5.531881<br>H 0.834348 3.970507 -5.394375<br>C -0.793479 2.606778 -5.080201<br>H -0.936635 1.578658 -4.713685<br>H -1.395451 3.258822 -4.426600<br>C 1.935087 5.844330 -3.336262<br>H 1.975507 5.967564 -2.245102<br>H 0.884272 5.652225 -3.595094<br>C 2.400079 7.129393 -4.017881<br>H 3.462384 7.293912 -3.779691<br>H 2.343547 6.980945 -5.107568<br>C 5.290833 4.845408 0.838700<br>H 5.062189 5.912954 0.691492<br>H 5.441258 4.670424 1.914064<br>H 6.231459 4.626624 0.313821<br>C -1.251844 2.720482 -6.532882<br>H -0.615010 2.108310 -7.187380<br>H -2.292541 2.391935 -6.661220<br>H -1.184105 3.760732 -6.886996<br>C 1.586852 8.342995 -3.598595<br>H 1.585331 8.431107 -2.510188<br>H 2.000044 9.276702 -4.002746<br>H 0.539878 8.253588 -3.919781<br>C 2.995496 0.876936 -3.627434<br>H 2.427271 0.749105 -2.697187<br>H 2.322554 0.682780 -4.474951<br>C 4.102296 2.569199 -5.136472<br>H 4.863152 1.802684 -5.325285<br>H 3.338225 2.456985 -5.915176<br>H 4.605562 3.538394 -5.228541<br>C 4.115402 -0.156669 -3.588850<br>H 4.750745 0.029100 -2.717093<br>H 3.693478 -1.161975 -3.476714<br>H 4.736569 -0.152709 -4.496234	

$[N_{444t}]_2[VBH]$	$[N_{444t}][VBH]$
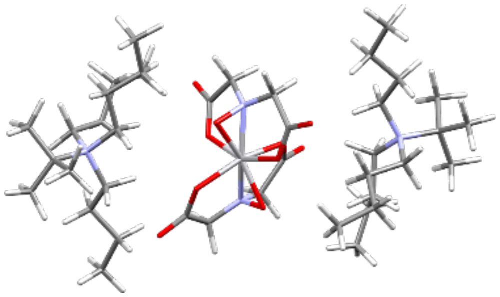	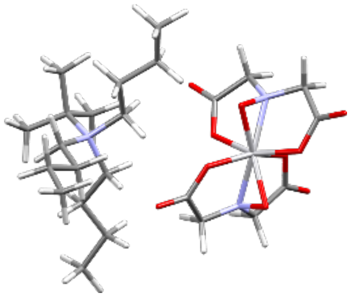
135	82
N -4.176116 -2.761755 3.185175	O -1.699050 0.968155 -0.383361
C -3.316497 -1.564453 3.670594	O -3.162418 2.534009 0.309690
H -2.978648 -1.050774 2.765647	O 2.540899 -0.683217 3.155572
H -4.016405 -0.891033 4.172185	O 1.531357 -0.743091 1.150475
C -5.330232 -3.047914 4.248465	O 0.524613 1.698720 0.795232
C -4.696460 -2.318278 1.807422	N -0.256732 1.045609 1.721703
H -3.785243 -2.137168 1.225711	C -2.216547 1.775168 0.513412
H -5.176412 -1.346661 1.961829	C -1.572859 1.656994 1.899276
C -3.340137 -4.008535 3.005364	H -2.185187 0.985931 2.519395
H -3.031736 -4.321943 4.007953	H -1.495660 2.639139 2.385745
H -4.002109 -4.782801 2.607142	C 0.487882 0.544513 2.882720
C -2.106600 -1.838790 4.572849	H 0.883835 1.368748 3.492755
H -2.162399 -2.817243 5.066225	H -0.202394 -0.059721 3.488306
H -1.200258 -1.856876 3.950145	C 1.625483 -0.356896 2.389603
C -1.934485 -0.763602 5.652522	V 0.000000 0.000000 0.000000
H -2.684219 -0.887831 6.446247	O 1.474699 0.601074 -1.178776
H -2.107785 0.224079 5.203348	O 3.019821 0.062046 -2.713448
C -5.621947 -3.246357 1.033375	O -1.813083 -3.466115 1.107951
H -5.323218 -4.300112 1.132981	O -1.027049 -1.362260 1.030887
H -6.649957 -3.146780 1.400744	O -0.654878 -0.894209 -1.584498
C -5.591729 -2.882857 -0.456912	N 0.358416 -1.686166 -1.086736
H -4.592501 -3.114662 -0.857020	C 2.083777 -0.241755 -1.976221
H -5.749077 -1.803814 -0.578273	C 1.579308 -1.678830 -1.884961
C -2.129749 -3.858336 2.104315	H 2.326714 -2.293104 -1.364001
H -1.578067 -2.934955 2.314539	H 1.379883 -2.111030 -2.874646
H -2.423183 -3.803906 1.049960	C -0.122914 -2.947445 -0.522162
C -1.191826 -5.054625 2.287756	H -0.606703 -3.541532 -1.309277
H -1.638867 -5.949649 1.825070	H 0.727160 -3.517102 -0.121788
H -1.077641 -5.277776 3.363196	C -1.080805 -2.617235 0.617160
C 0.173721 -4.756879 1.682632	C 5.109714 -1.483626 -0.421956
H 0.857829 -5.609686 1.777747	H 4.541393 -1.044134 -1.249578
H 0.085611 -4.476507 0.625596	H 4.386078 -1.830246 0.325980
H 0.615391 -3.892022 2.195427	C 4.663925 0.584156 0.707471
C -0.536195 -0.815738 6.255769	H 3.878322 0.476310 -0.047795
H -0.446396 -0.133890 7.110740	H 4.288360 0.092143 1.610471
H -0.296001 -1.826323 6.620333	C 6.683298 0.441052 -0.775878
H 0.222774 -0.549966 5.501907	H 7.119435 1.292759 -0.242641
C -6.674006 -3.621650 -1.244240	H 7.503189 -0.227471 -1.053439
H -6.579971 -3.408248 -2.319336	N 5.828168 -0.300228 0.230124

H -6.591812 -4.711395 -1.103133
H -7.674954 -3.300665 -0.923657
C -6.393167 -1.953946 4.152220
H -6.922153 -1.942413 3.191754
H -7.130471 -2.150198 4.941588
H -5.981580 -0.953708 4.342852
C -4.743198 -3.063532 5.658919
H -5.547461 -3.359934 6.343616
H -3.938916 -3.800768 5.774813
H -4.383149 -2.083745 5.992448
C -5.988333 -4.411994 4.006890
H -6.391424 -4.531702 2.999705
H -5.307195 -5.243488 4.225119
H -6.825065 -4.484388 4.714234
V 0.000000 0.000000 0.000000
O 1.937080 0.177200 -0.703877
O 4.036815 0.831993 -0.222136
O 0.896774 -0.806483 1.577224
O -2.157172 1.409516 3.216191
O -1.571008 0.244286 1.374928
O -0.153522 2.078640 -0.179773
O -1.555075 3.727382 -0.795372
O -0.323306 0.059140 -1.951095
O -2.095220 -3.354662 -1.175018
O -0.578890 -1.975224 -0.239554
N 1.002228 0.575433 1.673057
N -1.542947 0.171009 -1.297624
C 2.839221 0.661701 0.076174
C 2.360820 1.070821 1.473194
H 3.045771 0.696305 2.248275
H 2.348377 2.168027 1.535043
C 0.178912 1.177561 2.720919
H 0.369321 2.258221 2.724290
H 0.440148 0.765462 3.707948
C -1.313266 0.940180 2.430969
C -1.229245 2.528624 -0.738694
C -2.154138 1.494145 -1.370629
H -3.097799 1.456261 -0.811182
H -2.363019 1.766157 -2.416653
C -2.390858 -1.001363 -1.461847
H -2.651960 -1.145687 -2.519860
H -3.320022 -0.839716 -0.896481
C -1.641587 -2.219135 -0.929383
N 2.980378 4.398756 -2.466504
C 3.526266 3.925164 -1.106112
H 4.104268 3.018067 -1.303102
H 2.640483 3.602147 -0.545592
C 4.094546 4.251857 -3.597330
C 1.776038 3.478826 -2.715653
H 2.136422 2.452686 -2.576452
H 1.089576 3.682400 -1.884554
C 2.507166 5.839848 -2.369580
H 3.409881 6.437753 -2.206095
C 6.728446 -0.764659 1.463223
C 5.917283 -2.653258 -0.954463
H 6.562912 -2.329898 -1.787819
H 6.566013 -3.093192 -0.191291
C 4.858608 2.075529 0.955397
H 4.919927 2.626315 0.006001
H 5.753862 2.334067 1.524939
C 5.970161 0.913136 -2.031240
H 5.701801 0.063715 -2.677228
H 5.023217 1.418186 -1.800954
C 5.959532 -1.808539 2.276016
H 4.977039 -1.450947 2.612384
H 5.829649 -2.753795 1.741381
C 3.617512 2.518557 1.742884
H 2.716016 2.298357 1.154143
H 3.540404 1.896018 2.648056
C 3.637315 3.980627 2.154285
H 3.658802 4.639195 1.277315
H 2.746530 4.217810 2.751529
H 4.530944 4.213048 2.745071
C 4.969380 -3.763427 -1.422189
H 4.437270 -4.166224 -0.545072
H 4.199897 -3.347198 -2.091066
C 5.727100 -4.889610 -2.119380
H 6.235815 -4.528522 -3.026898
H 6.488878 -5.318704 -1.453926
H 5.064747 -5.714553 -2.405654
C 6.887858 1.867889 -2.800280
H 7.113456 2.741757 -2.169676
H 7.863795 1.380019 -2.970811
C 6.268621 2.310830 -4.121680
H 6.038760 1.453023 -4.770570
H 5.319348 2.836594 -3.947059
H 6.939033 2.992138 -4.660596
C 8.082588 -1.302758 0.990928
H 8.559899 -1.805696 1.838705
H 8.043040 -2.034659 0.183841
H 8.749292 -0.495859 0.673514
H 6.560995 -2.030521 3.165228
C 7.043072 0.424261 2.368836
H 6.177165 0.810312 2.911716
H 7.763239 0.058431 3.112662
H 7.519056 1.250954 1.828501

H	2.101845	6.100648	-3.351552
C	4.327832	4.886184	-0.239152
H	3.803778	5.841167	-0.103676
H	5.314688	5.094678	-0.659389
C	4.533968	4.214119	1.127791
H	3.591175	4.256464	1.698700
H	4.761722	3.146425	0.981724
C	1.031732	3.605089	-4.033415
H	1.614382	3.185107	-4.862484
H	0.806151	4.657383	-4.265567
C	-0.279520	2.820062	-3.934355
H	-0.074723	1.826856	-3.507128
H	-0.946591	3.329757	-3.223727
C	1.452948	6.119806	-1.292166
H	1.713799	7.076464	-0.820834
H	1.479048	5.356950	-0.500538
C	0.021566	6.249026	-1.819334
H	0.008805	7.033089	-2.593678
H	-0.309617	5.306951	-2.277902
C	5.671814	4.851539	1.920683
H	5.500944	5.925798	2.087682
H	5.779303	4.360745	2.895895
H	6.613126	4.733522	1.366760
C	-0.953978	2.668602	-5.294135
H	-0.268649	2.197019	-6.013428
H	-1.857920	2.045501	-5.219684
H	-1.252262	3.644992	-5.707716
C	-0.939500	6.625293	-0.696628
H	-0.688689	7.617658	-0.308374
H	-1.976986	6.644586	-1.054987
H	-0.891219	5.877903	0.104786
C	4.261842	2.770019	-3.935676
H	3.436997	2.364936	-4.533723
H	5.177915	2.682940	-4.532842
H	4.398333	2.146449	-3.043925
C	3.722891	5.047547	-4.857169
H	4.456479	4.775927	-5.627827
H	2.731706	4.831814	-5.259002
H	3.819709	6.127714	-4.688466
C	5.437118	4.803071	-3.111041
H	5.912950	4.192819	-2.334533
H	6.108441	4.801563	-3.980998
H	5.363697	5.839946	-2.757518

Symmetric disubstituted [N44xx]

$[N_{44cc}]_2[VBH]$

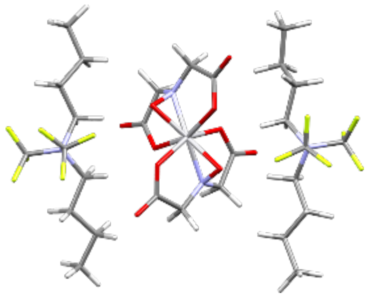

99

N -1.108514 -4.294314 2.440665
C -0.140177 -3.441302 3.335072
H 0.201259 -2.674833 2.627551
H -0.813255 -2.999106 4.078770
C -1.984984 -5.213724 3.311170
C -1.999646 -3.222487 1.705898
H -1.275907 -2.666706 1.098305
H -2.322586 -2.577031 2.530284
C -0.312001 -5.103350 1.410450
C 1.040086 -4.117084 4.010042
H 0.705613 -4.720762 4.860550
H 1.610356 -4.751115 3.318315
C 1.969134 -3.008895 4.531647
H 1.394000 -2.281124 5.124610
H 2.397642 -2.466753 3.672200
C -3.175162 -3.703438 0.867069
H -2.840884 -3.914776 -0.155754
H -3.643196 -4.612093 1.261867
C -4.244127 -2.603562 0.817632
H -3.781084 -1.634071 0.583172
H -4.690343 -2.496030 1.820900
C 3.072618 -3.581677 5.415048
H 3.746846 -2.791063 5.766738
H 2.629257 -4.049145 6.304811
H 3.667654 -4.334755 4.875571
C -5.309738 -2.950501 -0.218145
H -6.141287 -2.236861 -0.206254
H -4.862138 -2.960395 -1.221812
H -5.728633 -3.951072 -0.020091
F -1.245908 -5.848635 4.222309
F -2.898962 -4.458979 3.917326
F -2.584387 -6.126837 2.537906
F 0.664002 -4.330319 0.933365
F 0.224223 -6.191111 1.998165
F -1.094284 -5.498268 0.410245
N 2.106813 4.181834 -2.268767
C 2.459513 3.787737 -0.773171

$[N_{44cc}][VBH]$

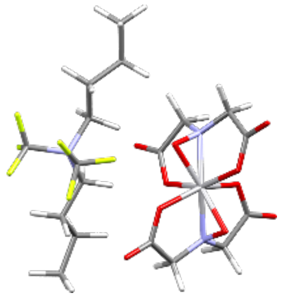

64

O -1.158085 1.497599 -0.569449
O -2.066944 3.492697 -0.068703
O 1.896336 -1.315354 3.383684
O 1.027394 -1.174181 1.312765
O 1.071228 1.416740 0.828039
N 0.004540 1.169811 1.666418
C -1.446888 2.481538 0.249814
C -0.970632 2.259377 1.688055
H -1.819343 1.954707 2.315187
H -0.525772 3.177105 2.098840
C 0.372541 0.487636 2.909591
H 0.946287 1.148759 3.575062
H -0.557588 0.183082 3.410308
C 1.177404 -0.765921 2.544496
V 0.000000 0.000000 0.000000
O 1.659787 -0.025625 -1.078155
O 2.942529 -1.086739 -2.580557
O -3.110113 -2.459965 0.872733
O -1.568831 -0.825247 0.935919
O -0.823248 -0.693557 -1.614116
N -0.185083 -1.756485 -1.008721
C 1.960163 -1.056412 -1.842099
C 0.999123 -2.227689 -1.724274
H 1.449924 -3.043489 -1.141527
H 0.700393 -2.628653 -2.703376
C -1.114034 -2.730597 -0.440533
H -1.671481 -3.218905 -1.251134
H -0.524517 -3.485369 0.098723
C -2.032219 -1.984688 0.519424
C 4.337355 -0.409957 0.675358
H 3.348741 -0.182761 0.260876
H 4.210702 -0.715438 1.720003
C 4.165914 1.698697 1.926305
H 3.137458 1.470246 1.624088
H 4.428211 1.110048 2.810194
N 5.005379 1.002152 0.800670
C 5.046999 -1.491940 -0.110352

H	2.476831	2.692396	-0.805375
H	1.572900	4.089446	-0.203864
C	2.801719	3.153402	-3.214295
C	0.552154	4.047334	-2.348928
H	0.378678	3.007397	-2.035464
H	0.189457	4.698923	-1.547372
C	2.546599	5.618474	-2.594624
C	3.725710	4.368582	-0.174731
H	3.678588	5.465631	-0.145529
H	4.618414	4.087776	-0.744965
C	3.890175	3.861472	1.265840
H	2.938560	3.962587	1.810403
H	4.137059	2.791482	1.234554
C	-0.157018	4.350264	-3.654975
H	0.315085	3.845468	-4.509030
H	-0.158738	5.422597	-3.873928
C	-1.608843	3.868573	-3.503908
H	-1.613129	2.777632	-3.367803
H	-2.028349	4.298981	-2.580495
C	4.998796	4.652681	1.956690
H	4.723082	5.715581	2.045637
H	5.204067	4.269859	2.962441
H	5.931096	4.586647	1.379514
C	-2.467978	4.259068	-4.704762
H	-1.939825	4.094281	-5.653821
H	-3.386087	3.661534	-4.732662
H	-2.740529	5.318873	-4.664699
F	4.063626	2.983580	-2.830666
F	2.139109	2.009652	-3.142615
F	2.776243	3.614083	-4.475576
F	2.072663	6.019195	-3.777589
F	3.882482	5.694431	-2.628478
F	2.055351	6.414125	-1.640997
V	0.000000	0.000000	0.000000
O	1.927089	0.311021	-0.687329
O	4.024822	0.861199	-0.090181
O	0.888463	-1.419774	1.082909
O	-1.897929	-0.435894	3.660376
O	-1.486114	-0.215988	1.452912
O	0.030500	2.018390	0.592606
O	-1.030009	3.986906	0.341840
O	-0.365597	0.857277	-1.757011
O	-2.380922	-2.559233	-2.198363
O	-0.740556	-1.692860	-0.919943
N	1.098444	-0.195306	1.704818
N	-1.561302	0.739873	-1.051735
C	2.871550	0.476078	0.175283
C	2.488644	0.247492	1.640300
H	3.164517	-0.481911	2.111950
H	2.574054	1.206015	2.170140
C	0.362261	-0.025816	2.954598
H	0.498536	1.014616	3.261997
H	0.737940	-0.681520	3.753279
H	4.965586	-1.307737	-1.186773
H	6.104691	-1.587014	0.161386
C	4.320399	3.183318	2.197965
H	4.391469	3.790204	1.289529
H	5.217501	3.375417	2.798924
C	3.068322	3.613860	2.984212
H	2.202616	3.562786	2.303495
H	2.875579	2.899977	3.798073
C	3.206265	5.013662	3.575327
H	3.407516	5.756993	2.793013
H	2.297852	5.291024	4.127545
H	4.043472	5.041022	4.286287
C	4.356249	-2.823027	0.223082
H	4.556947	-3.070867	1.277557
H	3.263539	-2.723340	0.133852
C	4.872042	-3.924773	-0.694897
H	4.603973	-3.709797	-1.738869
H	5.967626	-3.982873	-0.628960
H	4.459959	-4.906673	-0.430030
C	6.488211	0.861444	1.214543
F	6.925254	2.011239	1.741261
F	6.571268	-0.105191	2.117920
F	7.220989	0.568159	0.137742
C	4.880923	1.779952	-0.526418
F	5.675816	2.858274	-0.489161
F	5.236259	1.013020	-1.557578
F	3.611915	2.151099	-0.664411

C -1.134945 -0.246207 2.696109
C -0.956593 2.751946 0.186469
C -2.083174 2.006328 -0.533046
H -2.880682 1.757406 0.180634
H -2.510420 2.616718 -1.338311
C -2.479280 -0.220721 -1.667017
H -2.694239 0.080059 -2.705389
H -3.422715 -0.211565 -1.103519
C -1.838615 -1.605988 -1.611975

$[N_{44ss}]_2[VBH]$

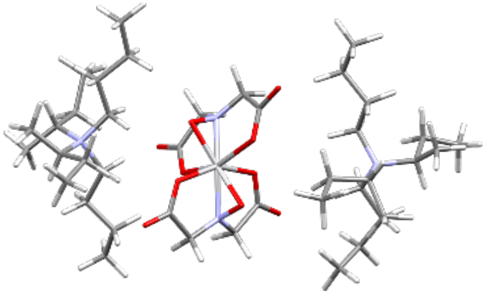

135

N -5.238933 -3.230709 1.372185
C -5.164588 -2.180070 2.503078
H -4.128709 -1.828066 2.517191
H -5.778142 -1.334618 2.167772
C -6.533513 -4.077848 1.548494
C -5.267298 -2.420070 0.074774
H -4.373315 -1.785842 0.112898
H -6.136223 -1.754842 0.139325
C -3.975453 -4.139973 1.371957
C -5.628667 -2.602451 3.898842
H -6.722237 -2.533307 3.968089
H -5.365693 -3.643447 4.121845
C -4.990700 -1.723652 4.983953
H -5.575582 -1.838728 5.902514
H -5.080299 -0.663057 4.711277
C -5.307806 -3.231932 -1.209403
H -4.557352 -4.035328 -1.200034
H -6.294272 -3.701883 -1.321120
C -5.066412 -2.362188 -2.441766
H -4.083569 -1.878017 -2.369190
H -5.835157 -1.578126 -2.489795
C -3.529115 -2.052938 5.265866
H -3.163877 -1.421654 6.087857
H -3.407732 -3.105173 5.571855
H -2.890486 -1.871808 4.392819
C -5.110915 -3.211704 -3.706702
H -4.967627 -2.621644 -4.621030
H -4.322410 -3.976378 -3.683928
H -6.081419 -3.724640 -3.786497

$[N_{44ss}][VBH]$

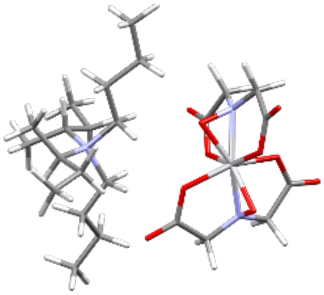

82

O -1.415351 1.304394 -0.525041
O -2.336606 3.325667 -0.147642
O 2.491448 -0.697752 3.200955
O 1.362118 -0.900034 1.262655
O 1.003094 1.597790 0.472552
N 0.119919 1.358625 1.502628
C -1.637293 2.375475 0.199430
C -0.960111 2.337380 1.572119
H -1.683139 1.998239 2.328116
H -0.573881 3.327728 1.850507
C 0.730113 0.869970 2.743143
H 1.306320 1.657741 3.247626
H -0.087403 0.528995 3.395254
C 1.617336 -0.330451 2.404509
V 0.000000 0.000000 0.000000
O 1.501622 -0.028327 -1.293926
O 2.672499 -1.153610 -2.837562
O -2.326374 -2.715324 1.942677
O -1.253905 -0.851956 1.301064
O -0.996436 -0.948046 -1.353701
N -0.156883 -1.877967 -0.776488
C 1.774802 -1.104441 -1.994775
C 0.928050 -2.321039 -1.650539
H 1.526516 -3.067734 -1.110206
H 0.505240 -2.796658 -2.545882
C -0.856620 -2.861157 0.047957
H -1.576621 -3.416682 -0.569200
H -0.116714 -3.570755 0.443624
C -1.556335 -2.128810 1.189030

C	-2.705708	-3.385401	0.951793
H	-2.593728	-2.432382	1.485058
H	-2.760324	-3.135034	-0.115664
C	-3.802600	-4.890280	2.684100
H	-4.734916	-5.356544	3.023825
H	-3.091592	-5.701638	2.492327
H	-3.410881	-4.246219	3.481209
H	-4.176636	-4.887235	0.600445
C	-1.446672	-4.224264	1.172444
H	-0.605738	-3.728018	0.676106
H	-1.204770	-4.308917	2.236678
H	-1.581438	-5.235134	0.770536
H	-6.550619	-4.290112	2.620428
C	-6.529440	-5.427802	0.836186
H	-5.758408	-6.113903	1.204813
H	-7.505384	-5.884610	1.047883
H	-6.424485	-5.348138	-0.249438
C	-7.778277	-3.250573	1.218446
H	-7.929773	-3.233544	0.128232
H	-7.621506	-2.209831	1.523727
C	-9.035268	-3.761207	1.924243
H	-9.195694	-4.837837	1.773670
H	-8.978063	-3.567551	3.002265
H	-9.915199	-3.229138	1.540558
N	3.459036	3.680472	-1.718559
C	2.956229	3.290455	-0.326003
H	3.183614	2.225478	-0.231019
H	1.866409	3.360351	-0.360496
C	4.948094	3.239377	-1.850771
C	2.545537	2.867677	-2.644348
H	2.616268	1.829363	-2.302864
H	1.531067	3.192785	-2.393713
C	3.292899	5.193451	-2.024304
C	3.437847	4.037051	0.905140
H	3.006319	5.043007	0.938250
H	4.532729	4.137951	0.944509
C	2.936458	3.252277	2.129144
H	1.934722	2.858036	1.897636
H	3.598118	2.386709	2.284674
C	2.766761	2.944364	-4.144332
H	3.672485	2.400998	-4.436550
H	2.852722	3.983000	-4.496461
C	1.576823	2.273037	-4.841518
H	1.442350	1.266776	-4.419645
H	0.652918	2.827784	-4.607112
C	2.856064	4.114240	3.384566
H	2.140187	4.935665	3.243027
H	2.524463	3.524416	4.252404
H	3.825157	4.563022	3.635933
C	1.766969	2.167254	-6.350618
H	2.658526	1.562131	-6.570121
H	0.899080	1.685071	-6.819332
H	1.905303	3.161601	-6.802085
C	4.845763	-1.110665	-0.243932
H	3.976698	-0.823739	-0.835461
H	4.461223	-1.564557	0.674089
C	4.367773	1.007224	0.816411
H	3.540142	0.976094	0.098762
H	4.050628	0.425026	1.687170
C	6.087155	0.915746	-1.050822
H	6.711639	0.141078	-1.510722
N	5.505774	0.197353	0.192911
C	6.593557	-0.064258	1.292674
H	6.672483	0.901031	1.801426
C	5.717566	-2.091315	-1.029483
H	5.529754	-1.958740	-2.106863
H	6.783447	-1.893753	-0.871082
C	4.646710	2.444911	1.227209
H	4.689680	3.097421	0.346660
H	5.595869	2.555514	1.764626
C	4.987618	1.298808	-2.040665
H	4.276316	0.482081	-2.210911
H	4.414795	2.143625	-1.634701
C	6.123266	-1.091778	2.328089
H	5.109475	-0.864837	2.684015
H	6.081437	-2.086334	1.864917
C	7.080917	-1.157977	3.519631
H	8.033010	-1.622352	3.236091
H	7.276555	-0.149437	3.905514
C	3.510664	2.918392	2.136901
H	2.566377	2.854371	1.578044
H	3.418315	2.225278	2.986281
C	3.718879	4.330669	2.659525
H	3.815088	5.043619	1.829902
H	2.882030	4.642223	3.299091
H	4.641952	4.408648	3.245074
C	5.439826	-3.547612	-0.642347
H	5.610125	-3.665727	0.440136
H	4.381264	-3.786740	-0.822660
C	6.329940	-4.520099	-1.410687
H	6.176139	-4.448986	-2.496848
H	7.391926	-4.308874	-1.204403
H	6.123866	-5.557703	-1.117909
C	5.588068	1.693793	-3.387470
H	6.282298	2.539259	-3.312599
H	6.124958	0.848192	-3.835714
H	6.634769	-1.761423	4.320615
C	7.974746	-0.453862	0.769828
H	8.697551	-0.234112	1.563473
H	8.056184	-1.531868	0.590357
H	8.311656	0.091984	-0.115293
H	4.777872	1.968444	-4.068206
C	6.972973	2.127259	-0.728975
H	7.428652	2.100957	0.263433
H	7.794698	2.178212	-1.452294
H	6.403090	3.058168	-0.815341

C 1.836814 5.680656 -1.926415<br>H 1.278690 5.114275 -1.169741<br>H 1.870551 6.716358 -1.560156<br>C 4.215777 6.103676 -1.211421<br>H 3.781446 6.376605 -0.248022<br>H 5.218569 5.698418 -1.038408<br>H 4.318593 7.036859 -1.779391<br>H 3.599595 5.256134 -3.074873<br>C 1.079283 5.696574 -3.246257<br>H 0.135500 6.240523 -3.127313<br>H 1.655208 6.244165 -4.001263<br>H 0.866498 4.695939 -3.637332<br>H 5.396262 3.584556 -0.909030<br>C 5.690624 3.896521 -3.013048<br>H 6.737925 3.574679 -2.955529<br>H 5.300285 3.579615 -3.985172<br>H 5.696956 4.989360 -2.977057<br>C 5.093108 1.713653 -1.911423<br>H 4.760445 1.335839 -2.890115<br>H 4.481121 1.221439 -1.144444<br>C 6.544988 1.301170 -1.667031<br>H 6.944049 1.769283 -0.756959<br>H 6.601779 0.215710 -1.528056<br>H 7.198651 1.562780 -2.509906<br>V 0.000000 0.000000 0.000000<br>O 2.081324 -0.011196 -0.185904<br>O 4.054256 0.000722 0.903886<br>O 0.331911 -1.486545 1.248007<br>O -2.756143 0.112371 3.071675<br>O -1.828515 0.026657 1.019063<br>O 0.073473 2.006445 0.622014<br>O -0.699414 4.084657 0.212601<br>O 0.218065 0.846077 -1.773929<br>O -1.845474 -2.219716 -2.965019<br>O -0.661636 -1.612764 -1.139382<br>N 0.615318 -0.301946 1.911969<br>N -1.120734 0.921405 -1.418528<br>C 2.807228 -0.061290 0.878672<br>C 2.040120 -0.154323 2.203527<br>H 2.398634 -0.991620 2.823848<br>H 2.189389 0.784773 2.752598<br>C -0.368688 0.064435 2.930364<br>H -0.138739 1.088219 3.260070<br>H -0.327612 -0.610296 3.796241<br>C -1.774485 0.063666 2.309425<br>C -0.665571 2.859256 -0.005982<br>C -1.563169 2.278189 -1.107542<br>H -2.595685 2.225735 -0.733518<br>H -1.531137 2.918806 -2.002369<br>C -1.961745 0.062534 -2.242814<br>H -1.954309 0.398322 -3.290037<br>H -2.989014 0.102717 -1.856891<br>C -1.451534 -1.380956 -2.134564	

[N$_{44tt}$]$_2$[VBH]

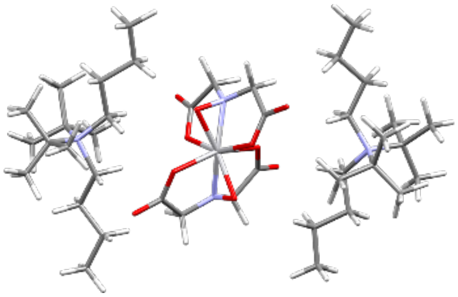

135

N	-4.017025	-3.207451	3.009311
C	-2.992250	-2.178473	3.557298
H	-2.335258	-1.968149	2.706096
H	-3.552272	-1.254215	3.721525
C	-5.299784	-3.252102	3.977909
C	-4.385969	-2.568954	1.631032
H	-3.485248	-2.718526	1.027079
H	-4.453265	-1.492327	1.823713
C	-3.253760	-4.606274	2.712202
C	-2.133403	-2.465290	4.791377
H	-2.681843	-2.306670	5.724056
H	-1.756251	-3.491662	4.816403
C	-0.931577	-1.517959	4.776009
H	-1.307043	-0.488648	4.730305
H	-0.348149	-1.674659	3.852717
C	-5.609169	-3.001420	0.812516
H	-5.790073	-4.076655	0.853833
H	-6.523008	-2.503322	1.158309
C	-5.385255	-2.640061	-0.662307
H	-4.429807	-3.066741	-1.001683
H	-5.306697	-1.551946	-0.770442
C	-0.056479	-1.685790	6.011272
H	0.783409	-0.977110	5.990750
H	-0.619837	-1.494804	6.935875
H	0.361360	-2.703162	6.078227
C	-6.529210	-3.154323	-1.529497
H	-6.395613	-2.842789	-2.574273
H	-6.560762	-4.253393	-1.504034
H	-7.499789	-2.773571	-1.182879
C	-1.831016	-4.320228	2.195843
H	-1.173204	-3.849836	2.931599
H	-1.400269	-5.298739	1.948992
H	-1.807577	-3.730667	1.271367
C	-3.151229	-5.518083	3.939495
H	-4.126394	-5.843359	4.310596
H	-2.618135	-6.416129	3.601605
H	-2.582936	-5.096136	4.771573
C	-3.951356	-5.420558	1.613810
H	-3.903053	-4.925165	0.639309

[N$_{44tt}$][VBH]

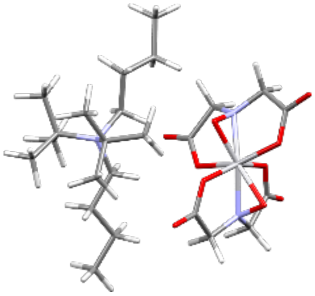

82

O	-1.314754	1.404538	-0.482176
O	-2.156189	3.440546	-0.018113
O	2.316257	-1.098747	3.190718
O	1.257788	-1.050243	1.212752
O	1.149767	1.486113	0.503615
N	0.250968	1.310767	1.528828
C	-1.465942	2.466410	0.274233
C	-0.741393	2.378869	1.617474
H	-1.469645	2.105454	2.390561
H	-0.271852	3.337293	1.880805
C	0.817486	0.733665	2.751847
H	1.487725	1.440403	3.259099
H	-0.020756	0.491108	3.419859
C	1.543941	-0.565952	2.388105
V	0.000000	0.000000	0.000000
O	1.445529	-0.073523	-1.333229
O	2.510175	-1.220652	-2.928995
O	-2.364433	-2.636143	2.029138
O	-1.335446	-0.773397	1.304303
O	-1.074253	-0.888486	-1.355006
N	-0.271052	-1.851760	-0.784314
C	1.651897	-1.154762	-2.047085
C	0.792971	-2.342026	-1.663933
H	1.392723	-3.074212	-1.104481
H	0.347781	-2.839642	-2.539384
C	-0.981281	-2.803501	0.072203
H	-1.742624	-3.333777	-0.516305
H	-0.250976	-3.533929	0.445026
C	-1.627620	-2.053902	1.235881
C	4.811393	-0.794532	-0.328531
H	3.939900	-0.506789	-0.922124
H	4.438534	-1.052674	0.668757
C	4.704436	1.242319	0.923200
H	3.683778	1.077469	0.558299
H	4.823501	0.649711	1.835209
C	5.560937	1.364213	-1.515110
N	5.602187	0.533437	-0.127529
C	7.052116	0.215532	0.506299
C	5.420218	-2.063069	-0.928445

H	-3.395262	-6.361051	1.517149
H	-4.983488	-5.685349	1.853128
C	-6.043743	-1.911558	3.884559
H	-6.509421	-1.723236	2.913989
H	-6.855352	-1.964227	4.621878
H	-5.414877	-1.061343	4.173600
C	-4.925049	-3.425098	5.459430
H	-5.832011	-3.750057	5.985867
H	-4.160954	-4.173466	5.664052
H	-4.621008	-2.479535	5.920766
C	-6.288899	-4.361483	3.591732
H	-6.674385	-4.263704	2.576099
H	-5.890105	-5.369808	3.735369
H	-7.141111	-4.254823	4.276522
V	0.000000	0.000000	0.000000
O	1.928152	0.171744	-0.723019
O	4.019789	0.848871	-0.249481
O	0.885890	-0.892375	1.537380
O	-2.146719	1.134002	3.313399
O	-1.561247	0.150741	1.376959
O	-0.117077	2.095932	-0.047102
O	-1.464994	3.823653	-0.562772
O	-0.365015	0.190044	-1.933498
O	-2.166699	-3.269696	-1.255096
O	-0.583095	-1.960041	-0.337211
N	1.011263	0.480954	1.686600
N	-1.563898	0.274075	-1.238699
C	2.831493	0.636609	0.065955
C	2.373647	0.968719	1.491152
H	3.063951	0.542988	2.232869
H	2.371296	2.059348	1.611150
C	0.185157	1.053784	2.750830
H	0.340271	2.140510	2.752762
H	0.467555	0.644258	3.731466
C	-1.302253	0.769975	2.475196
C	-1.189375	2.606948	-0.555948
C	-2.167109	1.610579	-1.187937
H	-3.070974	1.537447	-0.569733
H	-2.453902	1.946246	-2.194911
C	-2.427112	-0.888694	-1.427730
H	-2.735312	-0.977085	-2.477117
H	-3.327682	-0.746224	-0.816108
C	-1.678732	-2.149492	-0.988189
N	2.717014	4.603643	-2.099968
C	3.158885	4.050190	-0.716544
H	3.568054	3.058679	-0.925195
H	2.218485	3.883868	-0.177109
C	3.884728	4.308378	-3.166781
C	1.485211	3.714387	-2.424316
H	1.769516	2.697389	-2.130392
H	0.720878	4.029389	-1.708684
C	2.204020	6.127652	-1.919529
C	4.143077	4.779176	0.205457
H	5.807147	-1.915372	-1.941306
H	6.248097	-2.446997	-0.321665
C	4.827466	2.714360	1.319525
H	4.819265	3.400720	0.466837
H	5.731257	2.925546	1.894115
C	6.894095	-0.797221	1.656895
H	6.408346	-0.343800	2.528282
H	6.373781	-1.720029	1.398566
C	3.606615	3.030027	2.200027
H	2.705705	3.026178	1.567434
H	3.471397	2.216822	2.930471
C	3.746539	4.351512	2.945362
H	3.917513	5.181888	2.250578
H	2.850454	4.565197	3.545303
H	4.607737	4.320959	3.623489
C	4.335829	-3.147966	-0.985371
H	3.839465	-3.231826	-0.004009
H	3.570306	-2.843647	-1.707300
C	4.918829	-4.493946	-1.400816
H	5.451695	-4.421336	-2.360732
H	5.622225	-4.863129	-0.642757
H	4.136424	-5.254059	-1.512495
C	8.026232	-0.324491	-0.546182
H	8.947890	-0.577975	-0.013711
H	7.678952	-1.221312	-1.061783
H	8.310894	0.432228	-1.282188
H	7.909737	-1.081899	1.964007
C	7.721199	1.433784	1.161415
H	7.187070	1.755739	2.056186
H	8.703789	1.065779	1.489607
H	7.904222	2.276072	0.495514
C	4.152533	1.929941	-1.744665
H	4.134755	2.344667	-2.761624
H	3.362104	1.173651	-1.703284
H	3.896111	2.742071	-1.063501
C	5.839166	0.457642	-2.719119
H	5.005000	-0.227185	-2.910171
H	5.925580	1.119342	-3.591376
H	6.770526	-0.101887	-2.623607
C	6.555481	2.524545	-1.523791
H	6.424760	3.190945	-0.668446
H	7.602785	2.212388	-1.565578
H	6.367096	3.105048	-2.435757

H 3.746710 5.734948 0.569772<br>H 5.098415 4.993369 -0.279284<br>C 4.434863 3.883569 1.419421<br>H 3.524321 3.790547 2.035237<br>H 4.687406 2.867928 1.079948<br>C 0.880271 3.665375 -3.827224<br>H 1.493819 3.091094 -4.525731<br>H 0.747679 4.652196 -4.274560<br>C -0.495322 2.997297 -3.747357<br>H -0.382892 1.991523 -3.317679<br>H -1.133324 3.563715 -3.052558<br>C 5.580208 4.436392 2.267137<br>H 5.333826 5.432952 2.668838<br>H 5.799511 3.762375 3.106437<br>H 6.488346 4.521175 1.652933<br>C -1.154901 2.912894 -5.122015<br>H -0.491619 2.411903 -5.842404<br>H -2.107488 2.362454 -5.075942<br>H -1.366805 3.915935 -5.525550<br>C 3.970045 2.792261 -3.414884<br>H 3.149198 2.410743 -4.025690<br>H 4.896906 2.631860 -3.982093<br>H 4.051980 2.194535 -2.499416<br>C 5.276521 4.733765 -2.679156<br>H 5.674540 4.095034 -1.885512<br>H 5.948074 4.603995 -3.539212<br>H 5.346061 5.777091 -2.366885<br>C 3.632765 5.011756 -4.501762<br>H 3.709797 6.096251 -4.406364<br>H 4.439136 4.687619 -5.173981<br>H 2.690258 4.744195 -4.981222<br>C 1.270527 6.612174 -3.040171<br>H 1.691444 6.523866 -4.044698<br>H 0.297439 6.110647 -3.001029<br>H 1.096509 7.675530 -2.826570<br>C 3.359339 7.122868 -1.829702<br>H 4.103282 6.868017 -1.074414<br>H 3.869624 7.260258 -2.789301<br>H 2.914854 8.087867 -1.548825<br>C 1.353955 6.227278 -0.642910<br>H 0.463254 5.585971 -0.664666<br>H 1.910322 6.026571 0.275654<br>H 1.023978 7.272278 -0.600633	

Asymmetric disubstituted [N44xy]

[N$_{441c}$]$_2$[VBH]

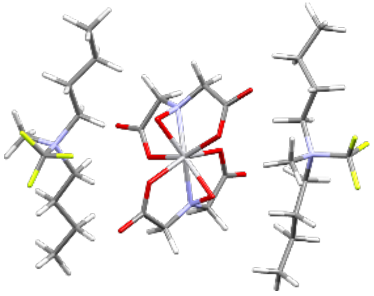

99

N -1.758798 -4.261183 2.463497
C -0.637015 -3.350618 3.022671
H -0.264864 -2.776795 2.164247
H -1.187066 -2.668328 3.680757
C -2.590775 -4.813277 3.589545
H -3.299324 -5.541953 3.185448
H -1.929792 -5.249181 4.339157
C -2.608511 -3.355842 1.529194
H -1.999063 -3.215676 0.629481
H -2.659744 -2.402119 2.066710
C -1.165318 -5.397396 1.674832
C 0.502017 -4.019653 3.769468
H 0.131371 -4.589543 4.633357
H 1.060258 -4.699509 3.109843
C 1.468121 -2.929554 4.259205
H 0.966674 -2.296753 5.007484
H 1.732835 -2.285012 3.406750
C -4.005881 -3.851669 1.228826
H -4.000128 -4.831254 0.738824
H -4.576042 -3.951950 2.164131
C -4.761871 -2.871865 0.327349
H -4.233645 -2.783191 -0.634606
H -4.782780 -1.871395 0.784872
C 2.729548 -3.544561 4.861299
H 3.433152 -2.768005 5.191671
H 2.487717 -4.167943 5.731182
H 3.239564 -4.180944 4.121001
C -6.182577 -3.384070 0.116819
H -6.790176 -2.705932 -0.489249
H -6.165968 -4.373536 -0.366295
H -6.695439 -3.486364 1.083411
H -3.111895 -3.966296 4.041306
F -0.204615 -4.929829 0.864659
F -2.091412 -6.020444 0.936083
F -0.635252 -6.313237 2.515763
N 1.820843 4.433610 -1.808732
C 2.090303 4.561825 -0.294093

[N$_{441c}$][VBH]

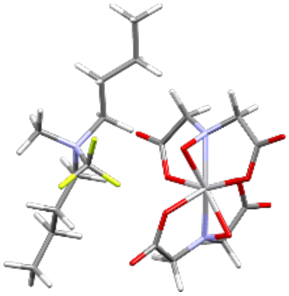

64

O -1.152588 1.581312 -0.342705
O -1.903896 3.568842 0.381074
O 1.997465 -1.723700 3.120251
O 1.046525 -1.316089 1.124484
O 1.233580 1.309771 0.745134
N 0.270157 1.077173 1.702136
C -1.279633 2.523845 0.556147
C -0.637023 2.205279 1.902553
H -1.418277 1.895205 2.610393
H -0.108568 3.080931 2.305667
C 0.734438 0.305464 2.856126
H 1.466622 0.870982 3.447550
H -0.140465 0.083377 3.480690
C 1.326074 -1.017023 2.363125
V 0.000000 0.000000 0.000000
O 1.536139 -0.098443 -1.246876
O 2.535150 -1.183358 -2.938999
O -3.157005 -2.293479 1.191737
O -1.531605 -0.746705 1.078127
O -1.034080 -0.530779 -1.546619
N -0.448972 -1.676769 -1.051530
C 1.660177 -1.112789 -2.075779
C 0.637633 -2.219860 -1.866854
H 1.088830 -3.042547 -1.293986
H 0.241928 -2.607866 -2.814684
C -1.421374 -2.586574 -0.449142
H -2.128902 -2.913445 -1.222373
H -0.874312 -3.453661 -0.056740
C -2.121702 -1.856250 0.688011
C 4.817399 -0.537582 0.149426
H 3.721314 -0.516317 0.102077
H 5.145442 -1.047308 1.064281
C 4.317534 1.464312 1.510555
H 3.349395 1.621786 1.021688
H 4.213299 0.607582 2.186691
N 5.252304 0.927392 0.400649
C 6.688911 0.975866 0.845445

H	1.636364	3.647216	0.110916
H	1.488606	5.416648	0.023148
C	2.216099	3.043510	-2.254291
H	3.208856	2.822248	-1.852661
H	2.195130	2.997891	-3.346876
C	0.305656	4.707859	-2.020935
H	-0.172584	4.406804	-1.081623
H	0.207483	5.797849	-2.109390
C	2.600096	5.467428	-2.580201
C	3.532184	4.745249	0.142161
H	3.887415	5.742486	-0.154879
H	4.208848	3.998053	-0.287950
C	3.581887	4.630150	1.672514
H	2.659556	5.043564	2.101300
H	3.622767	3.562020	1.929268
C	-0.337269	3.973910	-3.182501
H	-0.327933	2.893182	-2.977037
H	0.201568	4.145023	-4.125315
C	-1.792522	4.432641	-3.344483
H	-2.298843	4.328490	-2.372551
H	-1.811818	5.505848	-3.598330
C	4.780714	5.374187	2.251997
H	4.691519	6.446406	2.032997
H	4.839960	5.252656	3.341634
H	5.713169	4.999526	1.807390
C	-2.510995	3.619093	-4.420393
H	-2.493306	2.545384	-4.188436
H	-3.561711	3.917740	-4.512855
H	-2.048361	3.756909	-5.409206
H	1.493224	2.344727	-1.820099
F	2.426809	6.676462	-2.024238
F	2.154998	5.509967	-3.850108
F	3.905156	5.167687	-2.603829
V	0.000000	0.000000	0.000000
O	1.941644	0.561273	-0.442290
O	3.752722	1.626110	0.374946
O	0.934314	-1.105200	1.358842
O	-2.362846	-0.543039	3.349831
O	-1.640159	-0.360424	1.228304
O	-0.377553	2.046602	0.331448
O	-1.871929	3.696062	-0.017635
O	-0.229857	0.548174	-1.894487
O	-1.785650	-3.169428	-1.956399
O	-0.349241	-1.893713	-0.777521
N	0.812711	0.183377	1.857727
N	-1.486210	0.362349	-1.324517
C	2.666807	1.037495	0.513945
C	2.088423	0.892173	1.931122
H	2.803977	0.363978	2.581044
H	1.903413	1.897347	2.337679
C	-0.137457	0.297497	2.965032
H	-0.257529	1.364087	3.192071
H	0.211194	-0.223439	3.870052
H	7.321685	0.536460	0.074706
H	6.958012	2.023148	1.006398
C	5.405008	-1.217167	-1.072700
H	4.965280	-0.797724	-1.988588
H	6.495320	-1.080661	-1.102829
C	4.770724	2.682573	2.300643
H	5.129936	3.500039	1.665498
H	5.574868	2.399984	2.994725
C	3.560343	3.193482	3.096262
H	2.786203	3.521160	2.382557
H	3.132375	2.368742	3.684064
C	3.925223	4.339650	4.033408
H	4.309265	5.202324	3.473312
H	3.045470	4.642789	4.613182
H	4.690765	4.014603	4.752082
C	5.099468	-2.718536	-1.036746
H	5.552605	-3.149013	-0.131146
H	4.010837	-2.869918	-0.972307
C	5.642175	-3.407149	-2.287742
H	5.178903	-2.992626	-3.194958
H	6.732339	-3.283998	-2.367144
H	5.427619	-4.481997	-2.271692
H	6.753394	0.401911	1.774201
C	5.082065	1.759901	-0.855856
F	5.208044	3.063206	-0.569720
F	6.030349	1.440580	-1.757583
F	3.875543	1.537314	-1.382744

C -1.496278 -0.242386 2.503858
C -1.459256 2.532597 -0.195521
C -2.240591 1.596796 -1.113473
H -3.199607 1.355123 -0.629341
H -2.447318 2.086909 -2.076745
C -2.200460 -0.807794 -1.838214
H -2.382765 -0.688532 -2.917369
H -3.163845 -0.870931 -1.311850
C -1.389859 -2.067295 -1.524368

$[N_{44cs}]_2[VBH]$

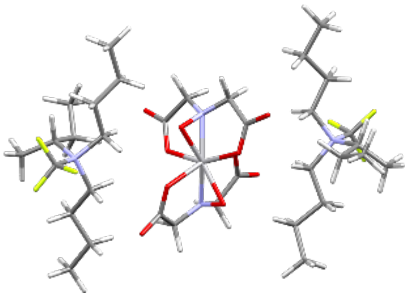

117

N -3.930686 -3.228622 2.442997
C -2.614553 -2.530993 2.880333
H -1.968003 -2.605748 1.997591
H -2.878448 -1.469896 2.988567
C -5.066806 -2.770651 3.337521
C -4.204423 -2.680290 1.003456
H -3.363984 -3.068544 0.424845
H -4.091723 -1.591858 1.077065
C -3.851682 -4.794094 2.381003
C -1.869602 -3.033381 4.112060
H -2.500384 -3.053256 5.008575
H -1.490266 -4.052370 3.954844
C -0.672610 -2.100524 4.356283
H -1.054935 -1.112239 4.650676
H -0.125874 -1.951189 3.412673
C -5.514465 -3.071769 0.338141
H -5.709509 -4.150203 0.442038
H -6.368163 -2.534980 0.772548
C -5.429198 -2.755159 -1.164707
H -4.474014 -3.129609 -1.563467
H -5.445608 -1.666078 -1.301436
C 0.270920 -2.648772 5.424892
H 1.131621 -1.980023 5.571682
H -0.230320 -2.762643 6.395975
H 0.662965 -3.635311 5.135243
C -6.604353 -3.388176 -1.906279
H -6.607720 -3.103771 -2.968497
H -6.540577 -4.486527 -1.853137
H -7.560657 -3.071977 -1.468265

$[N_{44cs}][VBH]$

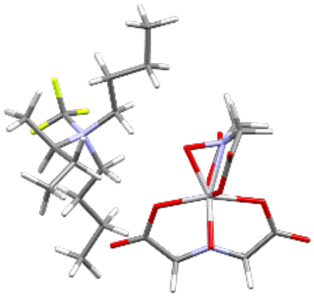

73

O -1.225109 1.482098 -0.483079
O -1.927839 3.562249 0.007042
O 2.081294 -1.327286 3.269530
O 1.140615 -1.159043 1.234764
O 1.113693 1.429826 0.721764
N 0.123696 1.182140 1.647504
C -1.385183 2.506609 0.322175
C -0.861698 2.258469 1.739001
H -1.690790 1.912538 2.373764
H -0.417844 3.168887 2.165878
C 0.588695 0.511260 2.862656
H 1.222148 1.173927 3.469125
H -0.299960 0.219161 3.440953
C 1.347959 -0.755014 2.454966
V 0.000000 0.000000 0.000000
O 1.577591 -0.011676 -1.205653
O 2.732751 -1.045660 -2.825485
O -2.886372 -2.592441 1.230126
O -1.463576 -0.854290 1.078105
O -0.941821 -0.717060 -1.531318
N -0.231397 -1.763908 -0.981369
C 1.816067 -1.030095 -2.006060
C 0.893277 -2.219156 -1.795535
H 1.412950 -3.013368 -1.238774
H 0.522657 -2.641509 -2.740057
C -1.075896 -2.756176 -0.325293
H -1.707260 -3.244984 -1.078260
H -0.422084 -3.507627 0.139133
C -1.900638 -2.050163 0.736873

F	-4.761361	-2.917396	4.641313
F	-5.318602	-1.475227	3.122335
F	-6.173975	-3.488923	3.079682
C	-2.592478	-5.247252	1.636523
H	-1.696902	-5.025727	2.231617
H	-2.480276	-4.709042	0.685740
C	-4.000404	-5.482447	3.730223
H	-4.940879	-5.248238	4.236027
H	-4.012516	-6.557541	3.526756
H	-3.166256	-5.269187	4.405056
H	-4.722317	-5.062550	1.773950
C	-2.656285	-6.748179	1.337104
H	-1.809063	-7.020479	0.701306
H	-2.587750	-7.356347	2.249508
H	-3.569724	-7.030940	0.794766
N	2.833459	3.587830	-2.766855
C	3.259312	3.311770	-1.302020
H	3.759322	2.336597	-1.331349
H	2.305120	3.161510	-0.781567
C	3.975958	3.235292	-3.689999
C	1.655549	2.601155	-3.048800
H	1.978939	1.624561	-2.672258
H	0.873306	2.952823	-2.373392
C	2.307771	5.048426	-3.024225
C	4.117304	4.347529	-0.587410
H	3.568807	5.293701	-0.479979
H	5.049379	4.545132	-1.128925
C	4.499825	3.836269	0.809894
H	3.595510	3.709841	1.423860
H	4.960399	2.841263	0.733731
C	1.150085	2.548895	-4.484473
H	1.738141	1.855537	-5.099742
H	1.220127	3.531247	-4.973220
C	-0.326052	2.140940	-4.511831
H	-0.432486	1.138945	-4.074808
H	-0.896863	2.828520	-3.865867
C	5.456408	4.806161	1.504757
H	5.008732	5.808154	1.600657
H	5.714008	4.444178	2.510809
H	6.381942	4.890986	0.917107
C	-0.867556	2.170937	-5.937140
H	-0.249465	1.538288	-6.589256
H	-1.908097	1.817676	-5.977969
H	-0.841897	3.192622	-6.350163
F	5.118610	3.823968	-3.309287
F	4.168225	1.903351	-3.691688
F	3.701878	3.626443	-4.949939
C	1.238192	5.422799	-1.992180
H	0.850646	4.559883	-1.441053
H	1.678174	6.115816	-1.267301
C	3.379802	6.118787	-3.154052
H	3.972188	6.249971	-2.248226
H	4.048980	5.958075	-4.003931
C	4.847154	-0.680881	-0.225364
H	3.842402	-0.432451	-0.571842
H	4.753507	-1.149034	0.760123
C	4.547787	1.367796	1.051756
H	3.561628	1.243359	0.591063
H	4.595202	0.715180	1.932541
C	5.674283	1.475098	-1.320118
H	6.064754	0.698984	-1.987960
N	5.511028	0.697456	0.039642
C	5.510418	-1.623000	-1.212532
H	5.371682	-1.252104	-2.238547
H	6.582419	-1.746440	-1.019189
C	4.725277	2.813019	1.487511
H	4.764044	3.509138	0.642675
H	5.636815	2.945492	2.079627
C	4.309412	1.930809	-1.830735
H	3.531986	1.182926	-1.639531
H	4.016663	2.847092	-1.301768
C	3.497800	3.147974	2.354199
H	2.612703	3.207293	1.702039
H	3.308876	2.317203	3.051882
C	3.666754	4.430124	3.162377
H	3.866919	5.292703	2.515894
H	2.776101	4.625338	3.774152
H	4.517050	4.324296	3.850621
C	4.848142	-3.001194	-1.106034
H	5.077149	-3.430321	-0.117138
H	3.755347	-2.895526	-1.164897
C	5.336355	-3.918733	-2.219914
H	5.019178	-3.561719	-3.208317
H	6.435689	-3.974776	-2.224403
H	4.949908	-4.937366	-2.097064
C	4.347649	2.178182	-3.340274
H	5.105095	2.919983	-3.629145
H	4.560090	1.241711	-3.874749
C	6.872080	0.449358	0.681669
F	7.356988	1.571371	1.230862
F	6.757834	-0.481147	1.631882
F	7.732382	0.028680	-0.259200
H	3.373056	2.543180	-3.686644
C	6.677121	2.617312	-1.258190
H	6.426724	3.338537	-0.476058
H	7.711884	2.293433	-1.114858
H	6.636875	3.142529	-2.220224

H 2.836982 7.061673 -3.307890
H 1.825283 4.941401 -4.000469
C 0.056478 6.098637 -2.683404
H 0.372543 7.011823 -3.202638
H -0.417129 5.408505 -3.398201
H -0.702384 6.377583 -1.944262
V 0.000000 0.000000 0.000000
O 1.975740 0.155994 -0.618856
O 4.086261 0.524976 0.069261
O 0.845571 -0.855338 1.576822
O -2.235317 0.743324 3.400339
O -1.607234 0.164804 1.314552
O -0.147153 2.096159 -0.172730
O -1.389045 3.763640 -1.043243
O -0.201993 0.103929 -1.952342
O -1.982324 -3.357883 -1.343203
O -0.561221 -1.970738 -0.285906
N 0.929736 0.523234 1.723800
N -1.465240 0.185911 -1.381801
C 2.853228 0.518727 0.251302
C 2.290394 1.026376 1.581493
H 2.928623 0.729570 2.419497
H 2.242973 2.124241 1.548401
C 0.066287 1.054750 2.777823
H 0.130687 2.152205 2.748401
H 0.383732 0.696670 3.769300
C -1.386496 0.629624 2.495994
C -1.147837 2.555211 -0.854821
C -2.080687 1.508756 -1.469089
H -3.016056 1.465135 -0.893710
H -2.298132 1.765874 -2.513979
C -2.279356 -0.996362 -1.641767
H -2.455327 -1.120292 -2.721637
H -3.247280 -0.863894 -1.136015
C -1.554815 -2.218140 -1.070757

$[N_{44ct}]_2[VBH]$

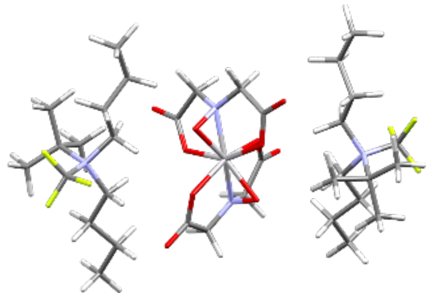

117

N -2.807524 -3.553007 2.990365
C -2.093880 -2.400620 3.768396
H -1.139233 -2.289930 3.243337

$[N_{44ct}][VBH]$

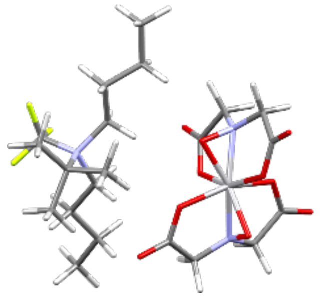

73

O -1.227923 1.471464 -0.525855
O -2.027278 3.517377 -0.022417
O 1.885905 -1.377672 3.378031

H -2.677536 -1.500391 3.553653	O 1.119030 -1.154246 1.273679
C -3.950968 -4.056421 3.840234	O 1.074784 1.434096 0.766944
C -3.421897 -2.827069 1.744703	N 0.054465 1.173531 1.652738
H -2.593756 -2.226303 1.350422	C -1.447020 2.479653 0.288027
H -4.134756 -2.121705 2.188778	C -0.948295 2.235419 1.711937
C -1.728922 -4.710129 2.577953	H -1.785753 1.879054 2.326712
C -1.897015 -2.505619 5.276587	H -0.529211 3.150946 2.152120
H -2.854324 -2.388435 5.801297	C 0.475276 0.501886 2.884114
H -1.470800 -3.462008 5.594314	H 1.094864 1.163489 3.506097
C -0.962736 -1.373360 5.720398	H -0.433138 0.226683 3.438094
H -1.352910 -0.418034 5.337842	C 1.233510 -0.777883 2.514987
H 0.026269 -1.522139 5.252926	V 0.000000 0.000000 0.000000
C -4.109879 -3.641001 0.655362	O 1.607488 0.011548 -1.161408
H -3.383511 -4.006455 -0.078234	O 2.805578 -0.992084 -2.768003
H -4.641183 -4.509825 1.050975	O -2.842298 -2.647709 1.209587
C -5.144630 -2.760072 -0.053929	O -1.472278 -0.871602 1.039427
H -4.649454 -1.934310 -0.579759	O -0.894743 -0.729109 -1.556855
H -5.813243 -2.301042 0.690620	N -0.181782 -1.764582 -0.989095
C -0.837308 -1.338667 7.238538	C 1.871548 -0.994930 -1.964744
H -0.231819 -0.490434 7.575676	C 0.970245 -2.200889 -1.775841
H -1.819128 -1.238160 7.717851	H 1.498842 -2.979264 -1.204944
H -0.362876 -2.253882 7.619889	H 0.631156 -2.634854 -2.727010
C -5.986976 -3.578818 -1.022397	C -1.022663 -2.771095 -0.350007
H -6.789785 -2.967753 -1.452486	H -1.639279 -3.265420 -1.111445
H -5.372771 -3.993985 -1.835729	H -0.360826 -3.515829 0.114195
H -6.452029 -4.422648 -0.489599	C -1.873257 -2.082878 0.710519
F -3.488733 -4.764486 4.885214	C 4.708608 -0.680083 -0.012114
F -4.660734 -3.008842 4.305930	H 3.983246 -0.424387 -0.785499
F -4.801344 -4.848007 3.167661	H 4.152700 -0.891890 0.907110
C -0.889874 -4.140878 1.438055	C 4.464148 1.388824 1.240047
H -0.372879 -3.209397 1.695489	H 3.488686 1.283755 0.752787
H -0.115420 -4.889996 1.223503	H 4.472351 0.738890 2.123297
H -1.444867 -3.971008 0.514931	C 5.631648 1.492679 -1.145955
C -2.438909 -5.997060 2.158975	N 5.435615 0.671965 0.251824
H -3.116418 -5.884465 1.310195	C 5.487361 -1.931018 -0.394450
H -1.648943 -6.691700 1.845636	H 6.084300 -1.791612 -1.301992
H -2.970931 -6.471388 2.992246	H 6.175220 -2.239911 0.403107
C -0.785166 -5.022632 3.743297	C 4.671254 2.819339 1.712369
H -1.291543 -5.430205 4.622896	H 4.743999 3.554538 0.904545
H -0.098826 -5.792918 3.366884	H 5.573268 2.907685 2.326322
H -0.176093 -4.158205 4.029635	C 4.285606 2.112303 -1.507498
N 2.641005 3.948122 -2.603378	H 3.471804 1.380747 -1.569945
C 3.075207 3.446732 -1.182947	H 3.999684 2.917742 -0.830450
H 3.409341 2.413368 -1.340156	C 3.444551 3.149781 2.582042
H 2.120946 3.390210 -0.644165	H 2.550781 3.183176 1.939588
C 3.752919 3.653981 -3.570885	H 3.269898 2.327005 3.292888
C 1.409635 3.050624 -2.940875	C 3.595046 4.449956 3.365470
H 1.604499 2.080784 -2.471088	H 3.721619 5.310684 2.697958
H 0.609124 3.528287 -2.368622	H 2.722370 4.611677 4.012894
C 2.191812 5.533456 -2.584084	H 4.481630 4.400266 4.012065
C 4.112173 4.223722 -0.376847	C 4.457545 -3.044243 -0.638132
H 3.863798 5.288092 -0.316585	H 3.787251 -3.126506 0.233248
H 5.119382 4.155446 -0.804609	H 3.833111 -2.770278 -1.498994

C 4.163100 3.690125 1.062942
H 3.149262 3.725547 1.499839
H 4.465261 2.633717 1.066251
C 1.000436 2.865842 -4.403056
H 1.574113 2.062348 -4.881513
H 1.172697 3.762736 -5.003062
C -0.497881 2.535789 -4.459946
H -0.668091 1.576554 -3.952014
H -1.049572 3.297390 -3.881856
C 5.132567 4.514520 1.910317
H 4.821426 5.570792 1.958589
H 5.190594 4.122666 2.935569
H 6.138404 4.483516 1.469402
C -1.027410 2.489929 -5.891605
H -0.437225 1.798969 -6.507542
H -2.081547 2.175535 -5.910740
H -0.963171 3.482276 -6.365378
F 4.931369 4.137418 -3.140701
F 3.886626 2.319857 -3.718515
F 3.509590 4.190821 -4.783001
C 1.407945 5.761529 -1.302646
H 0.503205 5.147079 -1.219580
H 2.000860 5.633316 -0.397663
H 1.114809 6.818726 -1.356152
C 1.258701 5.874651 -3.747864
H 1.013835 6.935830 -3.598552
H 1.729459 5.765003 -4.729284
H 0.320996 5.308306 -3.711749
C 3.413260 6.442233 -2.666653
H 4.166941 6.247984 -1.906056
H 3.893264 6.391540 -3.646899
H 3.030721 7.461039 -2.514878
V 0.000000 0.000000 0.000000
O 1.967954 0.126214 -0.562336
O 4.068824 0.587167 0.083193
O 0.729704 -1.124381 1.488369
O -2.243204 0.722470 3.394372
O -1.612196 0.101684 1.318008
O -0.011381 2.111122 0.150591
O -1.118752 3.969008 -0.492412
O -0.146847 0.433554 -1.918034
O -2.115795 -2.942636 -2.009833
O -0.663890 -1.859884 -0.660806
N 0.936966 0.215609 1.794297
N -1.416651 0.499937 -1.357167
C 2.860209 0.414799 0.319514
C 2.346868 0.593062 1.750401
H 2.941610 -0.003568 2.458085
H 2.442707 1.650027 2.031654
C 0.096461 0.729296 2.875550
H 0.285055 1.808064 2.966101
H 0.332117 0.238962 3.832654
C -1.383048 0.513768 2.517881
C 5.136145 -4.380348 -0.903604
H 5.781218 -4.319978 -1.792082
H 5.756807 -4.672970 -0.045788
H 4.406367 -5.179838 -1.075366
C 6.745800 0.390601 0.963813
F 7.276188 1.506712 1.489703
F 6.527328 -0.477832 1.962653
F 7.627475 -0.130768 0.101732
C 6.692984 2.573535 -0.987275
H 6.490222 3.235389 -0.142334
H 7.706276 2.170198 -0.892005
H 6.670850 3.174613 -1.906416
H 4.407716 2.551271 -2.506400
C 6.034014 0.552303 -2.283092
H 6.985730 0.043600 -2.103397
H 5.243469 -0.167229 -2.529465
H 6.166178 1.207123 -3.154200

C -0.959296 2.730875 -0.471627
C -1.950824 1.853950 -1.237172
H -2.888307 1.778475 -0.668830
H -2.150915 2.283825 -2.227969
C -2.294122 -0.561564 -1.833107
H -2.458045 -0.471900 -2.917469
H -3.257615 -0.460686 -1.311579
C -1.649871 -1.904028 -1.497788

$[N_{441s}]_2[VBH]$

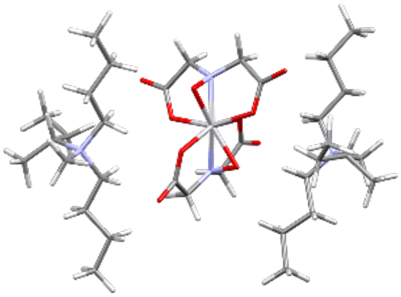

117

N -3.847421 -3.476751 1.745333
C -2.477167 -2.960609 2.202313
H -1.868650 -2.859333 1.294371
H -2.637031 -1.953245 2.597173
C -4.858449 -3.313794 2.902206
H -4.299178 -3.646173 3.787089
C -4.215149 -2.668656 0.499246
H -3.418185 -2.922609 -0.207623
H -4.114699 -1.610479 0.769434
C -3.693109 -4.909863 1.338944
H -3.479766 -5.513866 2.225233
H -4.586805 -5.288675 0.836362
C -1.771567 -3.842586 3.220271
H -2.447179 -4.120401 4.044174
H -1.434284 -4.779539 2.753010
C -0.563488 -3.135636 3.839658
H -0.901225 -2.208410 4.328068
H 0.138644 -2.834296 3.047097
C -5.559036 -2.951758 -0.159646
H -5.766537 -4.030661 -0.202369
H -6.384536 -2.468281 0.381441
C -5.535943 -2.411559 -1.596048
H -4.693939 -2.874013 -2.138304
H -5.346907 -1.329569 -1.564430
C 0.108722 -4.057780 4.853747
H 0.962015 -3.577532 5.352271
H -0.597101 -4.350575 5.643766
H 0.461518 -4.975692 4.362943
C -6.859764 -2.667229 -2.308985
H -6.834110 -2.298231 -3.344509

$[N_{441s}][VBH]$

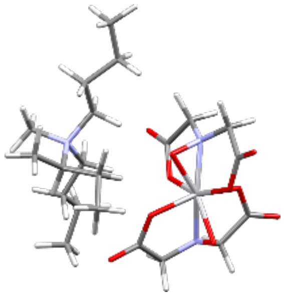

73

O -1.159097 1.571188 -0.304952
O -1.754491 3.624511 0.398342
O 2.228251 -1.647800 3.021066
O 1.105403 -1.311683 1.098090
O 1.215768 1.312283 0.774238
N 0.264062 1.036565 1.733111
C -1.235576 2.530267 0.596246
C -0.662448 2.142598 1.959073
H -1.477276 1.785831 2.608791
H -0.152544 2.996699 2.426366
C 0.784722 0.269506 2.864739
H 1.500730 0.866310 3.450420
H -0.064907 -0.016139 3.499378
C 1.448800 -0.997642 2.319621
V 0.000000 0.000000 0.000000
O 1.522059 0.024117 -1.272016
O 2.637689 -0.987287 -2.931328
O -3.153037 -2.356105 0.945341
O -1.504731 -0.829001 1.020469
O -1.006613 -0.560223 -1.581935
N -0.348460 -1.674155 -1.099728
C 1.730375 -0.968060 -2.101514
C 0.758715 -2.133712 -1.938667
H 1.258927 -2.965868 -1.422837
H 0.386194 -2.496667 -2.907855
C -1.252862 -2.657236 -0.503107
H -1.894933 -3.087661 -1.285561
H -0.646714 -3.450279 -0.040807
C -2.065772 -1.928904 0.561511
C 4.397276 -0.079668 0.350107

H	-7.086569	-3.743792	-2.334309
H	-7.682441	-2.161383	-1.791460
H	-2.846873	-4.948532	0.645483
C	-5.246470	-1.847891	3.087424
H	-5.949656	-1.583297	2.287648
H	-4.367761	-1.193528	2.997497
C	-5.900032	-1.592773	4.446804
H	-6.884253	-2.073813	4.523252
H	-5.277382	-1.946946	5.282384
H	-6.054159	-0.514622	4.587786
C	-6.083392	-4.202478	2.746409
H	-5.835910	-5.268214	2.682440
H	-6.714213	-4.055357	3.630313
H	-6.680757	-3.928081	1.874193
N	1.998081	3.631047	-3.107306
C	2.264447	3.588533	-1.596098
H	2.402637	2.529858	-1.354204
H	1.327586	3.911002	-1.122255
C	3.111226	2.816786	-3.809782
H	4.009576	3.085174	-3.238738
C	0.606167	3.049885	-3.305340
H	0.563811	2.112711	-2.738066
H	-0.062194	3.763276	-2.802303
C	1.982144	5.057728	-3.555002
H	2.977163	5.496023	-3.447776
H	1.646005	5.100998	-4.592744
C	3.430983	4.445102	-1.124769
H	3.182394	5.513259	-1.217210
H	4.332218	4.280165	-1.736625
C	3.802946	4.124328	0.328091
H	2.898149	4.147048	0.957433
H	4.191264	3.096967	0.392763
C	0.144331	2.808362	-4.732212
H	0.724048	2.013471	-5.218118
H	0.226036	3.715232	-5.350272
C	-1.316212	2.347260	-4.690875
H	-1.368076	1.441621	-4.068163
H	-1.928116	3.118272	-4.193103
C	4.834738	5.122974	0.849267
H	4.481649	6.159360	0.733501
H	5.059681	4.950525	1.910819
H	5.776517	5.019715	0.291455
C	-1.864967	2.047824	-6.079814
H	-1.264761	1.269796	-6.573638
H	-2.903652	1.694877	-6.019903
H	-1.855316	2.942529	-6.719340
H	1.297563	5.610927	-2.907095
C	2.897126	1.305386	-3.679527
H	2.262180	0.933338	-4.495958
H	2.388928	1.049287	-2.741075
C	4.243008	0.586598	-3.673708
H	4.794930	0.843762	-2.761204
H	4.098721	-0.500947	-3.674747
H	3.345065	0.156684	0.167268
H	4.484251	-0.476078	1.370820
C	4.438454	2.049630	1.466539
H	3.398667	2.131146	1.133549
H	4.443116	1.390481	2.345330
C	5.117171	1.917588	-1.027304
H	5.270089	1.067851	-1.703948
N	5.151413	1.248817	0.377639
C	6.567008	0.974741	0.771034
H	7.098860	0.525967	-0.069747
H	7.039682	1.919177	1.042014
C	4.883761	-1.118824	-0.637620
H	4.606614	-0.826080	-1.658773
H	5.971918	-1.253085	-0.580254
C	4.966583	3.409227	1.890156
H	5.090114	4.102815	1.051138
H	5.939508	3.309621	2.393190
C	3.755111	2.539376	-1.315764
H	2.950595	1.889944	-0.951545
H	3.683440	3.492837	-0.775356
C	3.932162	4.005914	2.865943
H	3.074769	4.373526	2.278334
H	3.550128	3.212092	3.524337
C	4.493287	5.125773	3.735570
H	4.892857	5.943306	3.122739
H	3.717319	5.518110	4.406728
H	5.299434	4.734673	4.371953
C	4.207608	-2.457002	-0.320418
H	4.643140	-2.858665	0.607687
H	3.139570	-2.293004	-0.107949
C	4.377993	-3.447696	-1.464533
H	3.905664	-3.071963	-2.383934
H	5.443699	-3.618481	-1.676476
H	3.929061	-4.420083	-1.227104
C	3.540154	2.755968	-2.814215
H	4.345572	3.338489	-3.280870
H	3.464543	1.790012	-3.330130
H	6.578871	0.291624	1.622031
H	2.600400	3.292788	-2.987437
C	6.263103	2.898431	-1.239794
H	6.274661	3.685566	-0.481141
H	7.247723	2.419157	-1.241188
H	6.123606	3.385199	-2.212982

H 4.862681 0.847024 -4.545083
C 3.349226 3.248029 -5.251508
H 4.198187 2.663040 -5.629050
H 2.482277 3.056623 -5.893577
H 3.631320 4.303230 -5.333409
V 0.000000 0.000000 0.000000
O 1.964307 0.234193 -0.656706
O 4.086454 0.663772 -0.028264
O 0.929090 -1.086972 1.383843
O -2.112363 0.271924 3.541791
O -1.557995 -0.031866 1.375095
O -0.143003 2.103375 0.169830
O -1.284896 3.918045 -0.536070
O -0.252601 0.419490 -1.902545
O -1.962072 -3.050580 -1.969632
O -0.596942 -1.892452 -0.600248
N 1.000342 0.254787 1.738277
N -1.504379 0.424640 -1.297023
C 2.869797 0.547462 0.208366
C 2.331213 0.849291 1.611407
H 3.018297 0.503480 2.391608
H 2.216552 1.939952 1.698574
C 0.181157 0.581486 2.901503
H 0.265850 1.659177 3.082575
H 0.530525 0.039250 3.791840
C -1.290698 0.249233 2.606159
C -1.111780 2.684507 -0.459097
C -2.104613 1.753489 -1.163028
H -3.013193 1.646923 -0.551736
H -2.374894 2.163446 -2.146884
C -2.318168 -0.693214 -1.755131
H -2.514902 -0.614886 -2.833615
H -3.276545 -0.672754 -1.216656
C -1.568190 -1.989783 -1.445774

$[N_{441t}]_2[VBH]$

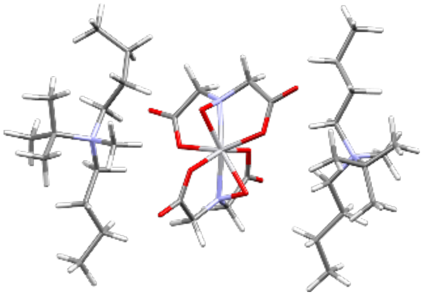

117

N -3.017763 -3.703792 2.681272
C -2.241660 -2.620353 3.445297
H -1.252734 -2.570229 2.977733
H -2.731997 -1.666742 3.222394

$[N_{441t}][VBH]$

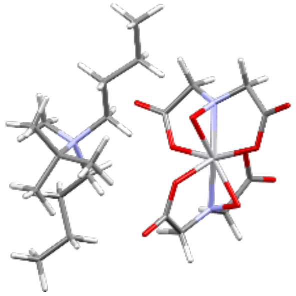

73

O -1.177566 1.533195 -0.391143
O -1.890325 3.553543 0.279404
O 2.084142 -1.562636 3.151654
O 1.062088 -1.281222 1.161932

C -4.143170 -4.196067 3.532873	O 1.055903 1.335215 0.936848
H -4.800444 -4.836126 2.941579	N 0.022159 0.987486 1.779077
H -3.744109 -4.755268 4.382268	C -1.383597 2.460393 0.514214
C -3.619373 -2.954330 1.480220	C -0.971789 2.051546 1.928728
H -2.778749 -2.453805 0.987630	H -1.843799 1.648270 2.464995
H -4.227046 -2.169705 1.951864	H -0.563403 2.909095 2.481347
C -2.045489 -4.893281 2.260511	C 0.444295 0.190335 2.934729
C -2.131142 -2.764976 4.956940	H 1.021256 0.799701 3.643540
H -3.060795 -2.442500 5.448359	H -0.458548 -0.197933 3.427582
H -1.943826 -3.795423 5.279534	C 1.280684 -0.982975 2.412858
C -0.982464 -1.881993 5.446233	V 0.000000 0.000000 0.000000
H -1.201598 -0.845250 5.151379	O 1.636279 0.126493 -1.126945
H -0.059142 -2.168143 4.912262	O 2.815697 -0.694960 -2.851193
C -4.486099 -3.718146 0.493688	O -3.087324 -2.577467 0.620741
H -3.869322 -4.214199 -0.266726	O -1.574171 -0.933392 0.863951
H -5.086317 -4.492797 0.989075	O -0.866905 -0.524354 -1.652390
C -5.446490 -2.730304 -0.178478	N -0.192612 -1.632687 -1.186329
H -4.876735 -1.958065 -0.712023	C 1.889878 -0.779835 -2.040115
H -6.033808 -2.204981 0.590562	C 0.969741 -1.991913 -1.993078
C -0.794042 -2.007353 6.949443	H 1.477727 -2.833195 -1.496163
H -0.087006 -1.267580 7.339187	H 0.649485 -2.292705 -2.998895
H -1.736687 -1.855992 7.488500	C -1.082566 -2.682985 -0.695870
H -0.410996 -3.002705 7.215213	H -1.643013 -3.124872 -1.533932
C -6.409019 -3.423828 -1.130077	H -0.454415 -3.442259 -0.210010
H -7.099501 -2.703878 -1.585170	C -2.009683 -2.048868 0.331428
H -5.871080 -3.967168 -1.922169	C 4.504600 -1.142685 -0.152745
H -7.019190 -4.149429 -0.573176	H 4.246645 -0.726939 -1.128732
H -4.696538 -3.324219 3.897308	H 3.568141 -1.276963 0.401994
C -1.144774 -4.407111 1.132326	C 4.156889 0.866149 1.169073
H -0.628449 -3.465277 1.349644	H 3.464563 1.070587 0.346643
H -0.370283 -5.166685 0.977977	H 3.636345 0.196384 1.866794
H -1.683039 -4.275214 0.186851	C 6.187493 0.779635 -0.440066
C -1.183160 -5.303354 3.456436	N 5.264221 -0.019291 0.572666
H -1.778587 -5.666603 4.305149	C 6.065443 -0.612970 1.690930
H -0.554593 -6.136574 3.122957	H 6.924522 -1.161745 1.293521
H -0.514945 -4.503818 3.795999	H 6.408993 0.177284 2.363751
C -2.842465 -6.115631 1.806486	C 5.216908 -2.489849 -0.306182
H -3.465390 -5.951633 0.924906	H 6.305860 -2.388717 -0.277199
H -2.119933 -6.891543 1.521236	H 4.957443 -3.148260 0.534967
H -3.460829 -6.539697 2.607683	C 4.543296 2.144959 1.895706
N 2.652649 3.786750 -2.628851	H 4.993203 2.882563 1.223633
C 2.777190 4.070824 -1.123150	H 5.272964 1.942766 2.689047
H 2.202981 3.254272 -0.663021	C 5.322856 1.712627 -1.290765
H 2.227336 5.002935 -0.947895	H 4.499030 1.181556 -1.787242
C 3.116193 2.378938 -2.854562	H 4.908292 2.549163 -0.720452
H 4.128614 2.268241 -2.464136	C 3.294965 2.796635 2.505865
H 3.086569 2.156070 -3.923542	H 2.616108 3.085627 1.691869
C 1.137647 3.886011 -2.918904	H 2.760591 2.057678 3.119691
H 0.636688 3.775323 -1.953267	C 3.644905 4.010587 3.362957
H 0.955295 4.913282 -3.249565	H 4.165575 4.768987 2.764148
C 3.449453 4.802886 -3.546829	H 2.740438 4.458752 3.799308
C 4.171061 4.148845 -0.516566	H 4.292115 3.721773 4.200115
H 4.620239 5.128753 -0.719530	C 4.833818 -3.172575 -1.626812

H	4.846639	3.406621	-0.949564
C	4.137184	3.905767	1.005626
H	3.141131	4.155458	1.406387
H	4.283380	2.832011	1.184664
C	0.569594	2.857014	-3.888904
H	0.526488	1.885198	-3.382348
H	1.177288	2.741489	-4.793394
C	-0.852882	3.262389	-4.299787
H	-1.381637	2.357207	-4.637132
H	-1.397597	3.637847	-3.420048
C	5.209786	4.714430	1.735044
H	5.012123	5.793899	1.637794
H	5.239916	4.463768	2.804503
H	6.196107	4.509982	1.294661
C	-0.878832	4.300005	-5.420608
H	-0.327410	3.930555	-6.297081
H	-1.908269	4.525025	-5.731402
H	-0.420299	5.250257	-5.105754
H	2.455987	1.701823	-2.303541
C	3.225613	6.222000	-3.035116
H	2.172084	6.519544	-2.988617
H	3.661877	6.392397	-2.047933
H	3.733233	6.909651	-3.725339
C	2.940353	4.691735	-4.988484
H	3.590914	5.331340	-5.599341
H	2.992909	3.674598	-5.397333
H	1.914031	5.056634	-5.106975
C	4.946070	4.474360	-3.565496
H	5.431963	4.507474	-2.587133
H	5.146114	3.503209	-4.034292
H	5.428369	5.237111	-4.190808
V	0.000000	0.000000	0.000000
O	1.952733	0.222589	-0.608496
O	4.057462	0.772862	-0.029765
O	0.834830	-1.035807	1.480688
O	-2.165121	0.639157	3.479640
O	-1.586626	0.073347	1.370701
O	-0.143494	2.117814	0.042990
O	-1.368319	3.871088	-0.697486
O	-0.231731	0.315536	-1.935434
O	-2.066032	-3.159475	-1.718431
O	-0.572742	-1.920835	-0.552780
N	0.977785	0.317829	1.762337
N	-1.482265	0.347981	-1.333570
C	2.861748	0.569637	0.239515
C	2.363356	0.773732	1.677620
H	3.006316	0.236039	2.391901
H	2.397435	1.846250	1.915934
C	0.146501	0.776695	2.872263
H	0.276698	1.863952	2.971955
H	0.440943	0.286475	3.814747
C	-1.331180	0.480289	2.564581
C	-1.137532	2.649865	-0.596258

H	3.787475	-3.513074	-1.587842
H	4.874196	-2.430771	-2.442165
C	5.756108	-4.346491	-1.941435
H	6.803440	-4.011741	-1.990203
H	5.688824	-5.123597	-1.168938
H	5.498139	-4.814536	-2.899996
H	5.409051	-1.310240	2.223672
C	7.266172	1.545472	0.324986
H	6.879372	2.223448	1.088385
H	7.986494	0.856550	0.783439
H	7.819299	2.133931	-0.414658
H	5.958033	2.140220	-2.078160
C	6.915870	-0.188456	-1.374215
H	7.554211	-0.893087	-0.831598
H	6.226410	-0.736569	-2.026122
H	7.572373	0.424815	-2.004733

C -2.090933 1.671577 -1.280490
H -3.013657 1.578715 -0.689338
H -2.335412 2.027410 -2.293897
C -2.323172 -0.780734 -1.704363
H -2.539240 -0.766546 -2.782448
H -3.269098 -0.689769 -1.151724
C -1.606450 -2.072778 -1.318881

[N_{44st}]$_2$[VBH]

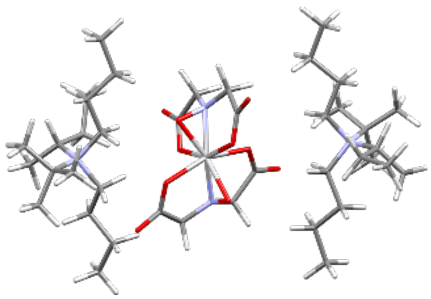

135

N -3.578121 -3.526986 2.983317
C -2.198823 -2.928930 3.290412
H -1.582959 -3.098282 2.400222
H -2.380669 -1.850285 3.331618
C -4.713348 -2.756231 3.849072
C -3.760875 -3.183828 1.487560
H -2.893357 -3.605393 0.970866
H -3.642955 -2.098168 1.421840
C -3.645321 -5.067100 3.145891
C -1.404542 -3.368091 4.519580
H -2.027556 -3.495938 5.408271
H -0.904960 -4.331556 4.334899
C -0.356221 -2.286000 4.805327
H -0.875252 -1.319007 4.868492
H 0.323622 -2.207903 3.940214
C -5.024471 -3.642429 0.781883
H -5.043650 -4.741493 0.697677
H -5.936017 -3.336369 1.306312
C -5.067219 -3.018373 -0.619082
H -4.127999 -3.226211 -1.152675
H -5.147059 -1.924890 -0.517779
C 0.415949 -2.517708 6.099002
H 1.178143 -1.736840 6.228193
H -0.246729 -2.458387 6.974010
H 0.928613 -3.490778 6.115259
C -6.257101 -3.541663 -1.412961
H -6.264695 -3.131670 -2.431079
H -6.209784 -4.638265 -1.490815
H -7.208485 -3.263896 -0.937571
C -2.632652 -5.765010 2.225712
H -1.627427 -5.352541 2.403517

[N_{44st}][VBH]

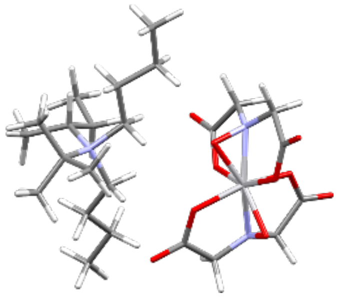

82

O -1.394298 1.321079 -0.531714
O -2.274508 3.367707 -0.177113
O 2.745636 -0.506678 3.007443
O 1.409226 -0.869522 1.232738
O 1.065111 1.596248 0.341278
N 0.229711 1.428271 1.422226
C -1.567331 2.421079 0.164223
C -0.829607 2.426657 1.501400
H -1.521138 2.126558 2.301427
H -0.418532 3.419754 1.726954
C 0.897745 0.987601 2.647825
H 1.482658 1.799880 3.099875
H 0.113384 0.655911 3.344864
C 1.779705 -0.211733 2.294502
V 0.000000 0.000000 0.000000
O 1.423421 -0.129062 -1.365492
O 2.564932 -1.380458 -2.830368
O -2.338266 -2.520977 2.174825
O -1.212110 -0.735893 1.407871
O -1.084526 -1.019596 -1.239051
N -0.222920 -1.919639 -0.646715
C 1.677700 -1.257880 -1.984357
C 0.814013 -2.434490 -1.544237
H 1.416096 -3.170503 -0.993681
H 0.348798 -2.949570 -2.397922
C -0.880434 -2.832545 0.289271
H -1.615271 -3.453189 -0.240140
H -0.116992 -3.497432 0.716526
C -1.554862 -2.009190 1.384431
C 4.715030 -0.569820 -0.575860
H 4.006763 -0.249543 -1.342465

H	-2.889193	-5.567789	1.180771
C	-3.523976	-5.583556	4.580082
H	-4.060177	-4.999284	5.328434
H	-3.961385	-6.589962	4.587549
H	-2.480305	-5.675353	4.896499
H	-4.649121	-5.314251	2.781896
C	-2.607869	-7.283289	2.410067
H	-1.982236	-7.741658	1.634675
H	-2.208766	-7.569613	3.390097
H	-3.608492	-7.717159	2.283313
C	-4.217011	-2.372242	5.245391
H	-5.037384	-1.818362	5.714112
H	-4.000599	-3.210372	5.909635
H	-3.363719	-1.686745	5.219150
C	-5.980041	-3.607596	3.990762
H	-6.359975	-3.991869	3.040327
H	-5.861020	-4.437062	4.695097
H	-6.749664	-2.947029	4.405048
C	-5.060826	-1.433790	3.155884
H	-5.609223	-1.554723	2.217297
H	-5.728486	-0.900225	3.844258
H	-4.177050	-0.796035	3.009676
N	2.386353	4.426020	-2.079987
C	2.714725	3.964966	-0.646210
H	3.294315	3.044747	-0.765763
H	1.758303	3.668872	-0.195769
C	3.603962	4.059028	-3.043414
C	1.136083	3.590945	-2.442423
H	1.384448	2.544333	-2.234323
H	0.390584	3.881969	-1.697603
C	1.951215	5.923729	-2.164302
C	3.454762	4.910783	0.294083
H	2.812308	5.756880	0.577384
H	4.350509	5.325434	-0.176818
C	3.903237	4.161283	1.556506
H	3.027516	3.921670	2.180363
H	4.360721	3.201565	1.269190
C	0.528701	3.749110	-3.828700
H	1.115776	3.251936	-4.607545
H	0.429702	4.809392	-4.104020
C	-0.872946	3.131185	-3.838654
H	-0.788072	2.048721	-3.672312
H	-1.440697	3.528533	-2.983514
C	4.911548	4.983374	2.361433
H	4.497902	5.966802	2.635096
H	5.198202	4.464579	3.287265
H	5.821829	5.141928	1.763561
C	-1.602546	3.424324	-5.147274
H	-1.020447	3.052828	-6.002236
H	-2.594099	2.945264	-5.173475
H	-1.751512	4.507348	-5.282719
C	0.774102	6.223242	-1.204653
H	0.649290	5.432916	-0.453963
H	4.118949	-0.861221	0.296706
C	4.348004	1.499516	0.575531
H	3.488004	1.490103	-0.101118
H	4.068060	0.890679	1.439894
C	5.976484	1.537469	-1.382239
N	5.456051	0.698467	-0.131948
C	6.543172	0.382248	0.963399
H	6.600374	1.311250	1.538988
C	5.505246	-1.787842	-1.058546
H	5.580328	-1.784668	-2.153390
H	6.528661	-1.790797	-0.670704
C	4.630557	2.914961	1.052039
H	4.770048	3.607138	0.212844
H	5.535035	2.979385	1.668137
C	6.052005	-0.701672	1.933504
H	5.034976	-0.485241	2.282991
H	6.002711	-1.672219	1.428294
C	6.972573	-0.821760	3.150792
H	7.916747	-1.320026	2.902010
H	7.195751	0.174860	3.552279
C	3.441992	3.385887	1.894278
H	2.519555	3.273036	1.305857
H	3.346237	2.730692	2.772828
C	3.605448	4.825333	2.357432
H	3.726373	5.493371	1.492927
H	2.738113	5.162007	2.940604
H	4.500177	4.946406	2.978302
C	4.826501	-3.094945	-0.642092
H	4.778133	-3.145955	0.458053
H	3.788742	-3.098244	-1.005359
C	5.584016	-4.296404	-1.196717
H	5.639136	-4.269289	-2.294379
H	6.611633	-4.297387	-0.801943
H	5.115218	-5.247847	-0.917589
H	6.476791	-1.408724	3.934124
C	7.952484	0.082616	0.467726
H	8.601099	0.075904	1.351918
H	8.060093	-0.906180	0.007280
H	8.355533	0.834139	-0.211935
C	4.778355	2.118487	-2.149831
H	5.195477	2.586416	-3.051260
H	4.058454	1.360796	-2.482339
H	4.238759	2.894413	-1.601409
C	6.702519	0.641384	-2.392608
H	5.986033	0.027819	-2.947782
H	7.184342	1.321101	-3.108503
H	7.472945	0.001520	-1.955597
C	6.907585	2.680176	-0.915996
H	7.036768	2.737153	0.165873
H	7.905349	2.588285	-1.355362
H	6.503922	3.639527	-1.255353

H 1.033543 7.143863 -0.665615<br>C 3.029797 6.984027 -2.010557<br>H 3.473001 7.013258 -1.015316<br>H 3.832051 6.928517 -2.748897<br>H 2.506335 7.942156 -2.144385<br>H 1.582784 6.002077 -3.191861<br>C -0.560324 6.442518 -1.915173<br>H -0.476594 7.274534 -2.626680<br>H -0.895895 5.537277 -2.434739<br>H -1.338938 6.697154 -1.185427<br>C 4.950861 4.430043 -2.412957<br>H 5.185523 3.847973 -1.516818<br>H 5.714636 4.188901 -3.161635<br>H 5.054334 5.491097 -2.176239<br>C 3.489089 4.775612 -4.392213<br>H 3.553241 5.862592 -4.305373<br>H 4.351309 4.446614 -4.987117<br>H 2.587297 4.512814 -4.949512<br>C 3.614272 2.544790 -3.298747<br>H 2.788170 2.223002 -3.942521<br>H 4.549371 2.328628 -3.834372<br>H 3.622698 1.940201 -2.382198<br>V 0.000000 0.000000 0.000000<br>O 1.905258 0.276664 -0.774020<br>O 4.019626 0.829384 -0.236639<br>O 1.014255 -1.048737 1.371371<br>O -2.003214 0.357665 3.594725<br>O -1.506849 -0.125770 1.454345<br>O -0.189722 2.106816 0.253193<br>O -1.532541 3.873477 -0.166914<br>O -0.426708 0.489709 -1.856300<br>O -1.917391 -2.995182 -2.066215<br>O -0.514038 -1.900674 -0.672106<br>N 1.053586 0.301280 1.689564<br>N -1.608841 0.416086 -1.129946<br>C 2.819931 0.648365 0.051268<br>C 2.344694 0.950011 1.476413<br>H 3.093269 0.656539 2.221016<br>H 2.176430 2.033069 1.552580<br>C 0.260568 0.674914 2.858418<br>H 0.314597 1.766157 2.967336<br>H 0.647283 0.198545 3.771682<br>C -1.205950 0.278297 2.636073<br>C -1.262606 2.657405 -0.221546<br>C -2.249059 1.706646 -0.894796<br>H -3.115763 1.539321 -0.242499<br>H -2.608176 2.129532 -1.842676<br>C -2.436814 -0.731023 -1.481157<br>H -2.880133 -0.615818 -2.477533<br>H -3.256470 -0.790377 -0.752574<br>C -1.565083 -1.987131 -1.420054	

References

1 D. F. C. Morris and E. L. Short, *Nature*, 1969, **224**, 950–952.

2 D. S. Palmer, A. Llinàs, I. Morao, G. M. Day, J. M. Goodman, R. C. Glen and J. B. O. Mitchell, *Mol. Pharm.*, 2008, **5**, 266–279.

3 D. S. Palmer, J. L. McDonagh, J. B. O. Mitchell, T. van Mourik and M. V. Fedorov, *J. Chem. Theory Comput.*, 2012, **8**, 3322–3337.

4 L. Cheng, P. Redfern, K. C. Lau, R. S. Assary, B. Narayanan and L. A. Curtiss, *J. Electrochem. Soc.*, 2017, **164**, E3696–E3701.

5 L. Chen and V. S. Bryantsev, *Phys. Chem. Chem. Phys.*, 2017, **19**, 4114–4124.

6 A. Gavezzotti and S. L. Price, in *Encyclopedia of Computational Chemistry*, ed. P. von R. Schleyer, John Wiley & Sons, Chichester, 1998, vol. 1, pp. 641–644.

7 D. A. McQuarrie and J. D. Simon, *Molecular Thermodynamics*, University Science Books, Sausalito, CA, 1999.

8 H. D. B. Jenkins, *J. Chem. Educ.*, 2005, **82**, 950.

9 V. S. Bryantsev, M. S. Diallo and W. A. Goddard III, *J. Phys. Chem. B*, 2008, **112**, 9709–9719.

10 V. S. Bryantsev, *Theor. Chem. Acc.*, 2012, **131**, 1–11.

11 A. V. Marenich, C. J. Cramer and D. G. Truhlar, *J. Phys. Chem. B*, 2009, **113**, 6378–6396.

12 C. J. Cramer and D. G. Truhlar, *J. Comput. Aided. Mol. Des.*, 1992, **6**, 629–666.
